# N6-methyladenosine RNA landscape in the aged mouse hearts

**DOI:** 10.3389/fcvm.2025.1563364

**Published:** 2025-06-18

**Authors:** Xia Jing, Yingming Li, Zhaofu Liao, Jing-Yu Wu, Zi-Yang Xu, Zhi-Peng Yang, Hai-Liang Mo, Shun Xu, Xinguang Liu, Zhuguo Wu, Jun Tao, Xing-Dong Xiong

**Affiliations:** ^1^ Dongguan Key Laboratory of Aging and Anti-Aging, The First Dongguan Affiliated Hospital, Guangdong Medical University, Dongguan, China; ^2^ Clinical Research Center, Affiliated Hospital of Guangdong Medical University, Zhanjiang, Guangdong, China; ^3^ Department of Cardiology, The First Dongguan Affiliated Hospital of Guangdong Medical University, Dongguan, Guangdong, China; ^4^ Department of Hypertension and Vascular Disease, The First Affiliated Hospital of Sun Yat-sen University, Guangzhou, Guangdong, China

**Keywords:** m6A RNA methylation, cardiac aging, METTL14, FTO, EFEMP1, cardiac diseases

## Abstract

**Objective:**

Cardiac aging is a major risk factor for the development of cardiovascular diseases. Although evidence suggests an association between N6-methyladenosine (m6A) modification and numerous cardiovascular diseases, its role in cardiac aging remains unclear. This study was conducted to elucidate the role of m6A modification in cardiac aging and the molecular mechanisms involved.

**Methods:**

Global methylation levels and the expression of major m6A regulators were compared between young and aged hearts. Transcriptome-wide m6A landscape analysis was conducted using methylated RNA immunoprecipitation sequencing (MeRIP-seq) and RNA sequencing (RNA-seq) to identify aberrant m6A peaks. Furthermore, gene set enrichment analysis (GSEA) was performed to identify gene sets associated with cardiac aging. Functional validation of key molecules was carried out through *in vitro* experiments.

**Results:**

The overall m6A level remained constant; however, the expression of the methyltransferase METTL14 and the demethyltransferase FTO were significantly upregulated in aged hearts. Knockdown of METTL14 alleviated H_2_O_2_-induced senescence phenotypes, as reflected by a reduction in the number of SA-β-gal positive cells and a decrease in p21 expression. Compared with young hearts, the dysregulated m6A peaks were significantly enriched in genes associated with dilated cardiomyopathy, hypertrophic cardiomyopathy, and the PI3K-Akt signaling pathway. GSEA showed that these genes were enriched in the aging of heart and aorta cardiomyocytes. Additionally, 255 genes with siginificantly changed of both m6A peaks and RNA expression were identified by combining MeRIP-seq and RNA-seq data. Among these genes, EFEMP1 was significantly upregulated in aged hearts, accompanied by enhanced m6A modification. Treatment with the methyltransferase inhibitor cycloleucine significantly suppressed the expression level of EFEMP1. In AC16 cells, silencing EFEMP1 suppressed H_2_O_2_-induced cell senescence. Furthermore, we found a positive correlation between METTL14 and EFEMP1 in multiple datasets related to cardiac aging.

**Conclusion:**

Our findings indicate that m6A modification plays an essential role in the process of cardiac aging. EFEMP1 may serve as a potential new therapeutic target for age-related cardiac diseases.

## 1 Introduction

Aging is a complex and universal biological process characterized by progressive functional decline of biological macromolecules and is regarded as an independent risk factor for cardiac diseases (1, 2). The process of cardiac aging involves a series of pathophysiological changes, including inflammation, increased oxidative stress, cardiac myocyte enlargement, increased interstitial fibrosis, and cardiac diastolic dysfunction (3, 4).

N6-methyladenosine (m6A) is the most prevalent post-transcriptional modification in mammalian RNA. Similar to DNA methylation and histone modification, the m6A RNA methylation is dynamically and reversibly regulated by methyl-transferases (“writers”), demethylases (“erasers”) and binding proteins (“readers”) (5). The writers responsible for m6A RNA methylation are METTL3 and METTL14 proteins, along with their cofactor WTAP. The removal of m6A RNA modification is carried out by two enzymes, FTO and ALKBH5, which act as “erasers”. Proteins containing YT521-Bhomology (YTH) domains, such as YTHDC1/2 and YTHDF1/2/3, act as “readers” that specifically recognize m6A modifications, thereby regulating processes including RNA splicing, localization, degradation, and translation. Furthermore, numerous studies have explored the functional roles of m6A in RNA regulation across various biological systems (6–8).

Recently, there has been growing interest in the role of m6A RNA modification as a new form of RNA epigenetic regulation, particularly in the context of human disorders, including cardiac ailments (3, 9). However, the transcriptome-wide m6A methylome of cardiac aging remains undetermined. Therefore, the aim of this study was to examine the level of m6A RNA modification in young and old hearts and to investigate the expression of m6A regulators. Moreover, we examined the differences in methylation peaks and gene expression by utilizing MeRIP-seq and RNA-seq. By analyzing MeRIP-seq and RNA-seq data, we were able to demonstrate specific m6A-modified RNA transcripts that exhibited higher differences in both m6A levels and expression patterns.

## 2 Materials and methods

### 2.1 Animals

Animal research was authorized by the Animal Care Committee of Guangdong Medical University and carried out in accordance with the Guide for the Care and Use of Laboratory Animals published by the US National Institutes of Health (NIH Publication, 8th Edition, 2011). The young group consisted of male C57BL/6 mice aged 6–7 weeks, which were purchased from Guangdong Yaokang Biotechnology Co., Ltd. The aged group was composed of male C57BL/6 mice that were also obtained from the same vendor. These aged mice were maintained in our animal center until they were 26–27 months old. The mice were raised under controlled environmental conditions with 12-h alternating dark/light cycles (22 ± 3 °C, 50%–60% relative humidity). All mice were provided *ad libitum* access to food and water. To eliminate gender-related effects, male mice were exclusively utilized in the current investigation.

### 2.2 Sample preparation

The hearts of the mice were quickly removed from the chest following euthanasia by cervical dislocation under 3% pentobarbital sodium anesthesia. Subsequently, the hearts were subjected to three washes with pre-chilled phosphate-buffered saline (PBS). The hearts were fixed in 4% paraformaldehyde at 4 °C overnight for histological staining. They were subsequently dehydrated using a sucrose gradient in PBS at 4 °C, embedded in OCT compound, sliced into 5-µm thick sections, and collected on siliconized slides.

### 2.3 Senescence-associated β-galactosidase (Sa-β-gal) staining

Senescence-associated β-gal (SA-β-gal) staining in heart tissues was performed with the SA-β-gal staining Kit (Beyotime) according to the manufacturer's instructions. Briefly, the sections at the level of the mid-papillary heart muscles were fixed using a fixative solution for 10 min at room temperature. After rinsing with PBS, the sections were subjected to incubation with a freshly prepared staining solution at 37 °C overnight, and subsequently examined via bright-field microscopy.

### 2.4 Masson's trichrome staining

The level of fibrosis in cardiac muscle and perivascular collagen volume area was assessed using a modified masson's trichrome stain kit (Solarbio; cat#G1343). The valuation was performed according to a well-established protocol, as delineated in a previous study (10). Cardiac slices isolated from the mid-papillary heart muscles were fixed in Bouin solution at 37 °C for 2 h. After fixation, the slices were stained with iron hematoxylin for 10 min and then rinsed with distilled water. The differentiation process involved treating the sample with a phosphomolybdic acid solution for 10 min, followed by staining with aniline blue for 5 min and 1% acetic acid for 1 min. Next, the sections were dehydrated, treated with xylene for cleaning, and finally mounted with resinene. Utilizing an optical microscope, the stained areas of the sections were visualized, with interstitial fibrosis appearing as a blue-stained region.

### 2.5 Isolation of mRNA

To extract total RNA from cardiac tissues, the TRIzol (Invitrogen, Canada) reagent was used, following manufacturer's instructions. Three heart tissues of aged mice were mixed as one aged sample and three heart tissues of young mice were mixed as one control sample, resulting in a total of three groups of young mice and three groups of aged mice. To disrupt secondary structures, the entire RNA was initially dissolved in a high-salt solution and briefly heated to 65 °C before being rapidly cooled on ice. At room temperature, the RNA is annealed to the oligo (dT) magnetic beads, and the poly(A)-oligo(dT) complexes are stabilized using high-salt binding buffer. Next, a high-salt washing buffer is used to eliminate unbound RNAs while keeping oligo(dT)-bound poly(A) + mRNAs intact. Finally, the mRNA is removed from the beads by introducing ribonuclease-free deionized water.

### 2.6 Quantification of m6A RNA methylation

The EpiQuik m6A RNA Methylation Quantification Kit (Epigentek) was utilized to measure the relative m6A content of mRNA. A standard curve was established by utilizing the positive control (included in the kit) at six varying concentrations, ranging from 0.01 to 0.5 ng/μl. In addition, 200 ng of mRNA was seeded in each well, followed by the addition of capture and detection antibody solutions according to the manufacturer's protocol. Following that, both the developer solution and stop solution were added. Furthermore, the m6A levels were measured colorimetrically by assessing the absorbance of each well at 450 nm, followed by calculating the percentage of m6A in the total mRNA.

### 2.7 RNA m6A dot blot assay

This method utilizes nylon membranes with positive charge to effectively bind mRNA molecules. The concentration of mRNA was quantified using Nanodrop 2000 (Denovix), and then further diluted to obtain the ladder concentration of 200, 100, and 50 ng/μl. One microliter of sample was applied onto the nylon membrane, and then subjected to UV cross linking. Unbound material was eliminated by washing with PBST, followed by blocking the membrane with a 5% blocking solution. The membrane was subjected to an overnight incubation with m6A antibody (1:1,000, Millipore) at 4 °C. The membrane underwent an overnight incubation at 4 °C with m6A antibody (1:1,000, Millipore). An HRP-conjugated anti-mouse IgG was incubated on the membrane at room temperature for 1 h, followed by detection using imaging equipment. In order to achieve uniform loading of the input, membranes were stained with 0.02% methylene blue (Sangon Biotech) for aduration of 2 min.

### 2.8 Western blot analysis

The protein portion of the heart tissue was lysed using RIPA lysis buffer (Beyotime) along with the addition of protease inhibitors. After quantification using the BCA protein kit (Beyotime), an equal amount of protein was separated on a 12.5% SDS-PAGE gel and subsequently transferred onto 0.22-μm PVDF membranes (Millipore, Billerica). The membranes were blocked with 5% skimmed milk for 1 h, at room temperature. Primary antibody incubation occurred at 4 °C overnight and secondary incubation occurred at room temperature for 1 h. Primary antibodies used were: anti p16 (1:500, Santa), anti p53 (1:1,000, CST), anti γH2A.X (1:1,000, Santa), anti METTL3 (1:1,000, Abcam), anti METTL14 (1:1,000, CST), anti WTAP (1:1,000, Proteintech), anti FTO (1:1,000, CST), anti ALKBH5 (1:1,000, Proteintech), anti YTHDC1 (1:1,000, CST), anti YTHDC2 (1:1,000, CST), anti YTHDF1 (1:1,000, CST), anti YTHDF2 (1:1,000, CST), anti YTHDF3 (1:1,000, CST) and anti GAPDH (1:10,000, Proteintech). Finally, SuperSignal™ ECL substrate (Thermo Scientific) was incubated with membranes for 2 min at room temperature and then exposed using the BioRad Chemidoc® imager. The quantification and normalization of positive immunoreactive bands were performed with respect to GAPDH. Images of Western blots were acquired and analyzed using Image J.

### 2.9 Cell culture and H_2_O_2_ treatment

Human cardiomyocyte AC16 cells were cultured in Dulbecco's Modified Eagle Medium(DMEM; Gibco) supplemented with 10% fetal bovine serum (FBS; Vazyme), 100 U/ml penicillin, and 100 μg/ml streptomycin (Gibco) at 37 °C in a humidified incubator with 5% CO2. Upon reaching 80% confluency, cells were dissociated using trypsin for passaging. To establish oxidative stress-induced senescence, cells were treated with 0, 50, 100, and 200 μM H_2_O_2_ (Sigma-Aldrich) and incubated for 24 h.

### 2.10 Cell transfection

AC16 cells were transfected with METTL14 siRNA (si-METTL14), EFEMP1 siRNA (si-EFEMP1), and scrambled siRNA (si-NC, all purchased from Tsingke) using Lipofectamine RNAiMAX Reagent (Invitrogen) according to the manufacturer's protocol. The sequences of si-METTL14 were sense 5′-UGGACUUGGGAUGAUAUUATT-3′ and antisense 5′-UAAUAUCAUCCCAAGUCCAGC-3′. The sequences of si-EFEMP1 were sense 5′-GCGUAGACAUAGAUGAAUGUA-3′ and antisense 5′-UACAUUCAUCUAUGUCUACGC-3′. After 24 h of transfe- ction, cells were then exposed to H_2_O_2_, with a final concentration of 100 µM for 24 h.

### 2.11 MeRIP-seq

Analysis of MeRIP-seq and RNA-seq data was performed by Guangzhou Epibiotek Co. Ltd. Three pairs of cardiac tissues (50–100 mg each) were used to extract total RNA using TRIzol reagent (Invitrogen) following the manufacturer's instructions. Using 10 × RNA Fragmentation Buffer, the total RNA was fragmented into 100–200 nt RNA fragments. The reaction was halted by incorporating 10 × EDTA (0.5M). Next, the RNA fragment was split into two portions, one for immunoprecipitation of methylated RNA and the other serving as an input sample for generating a standard transcriptome sequencing library.

The clean reads from all libraries were aligned to the mouse Ensemble genome GRCm38 using the Hisat2 aligner (v2.1.0) with the “rna_strandness RF” parameter to determine m6A peaks. The exomePeak R package (v2.13.2) was used to identify these peaks with parameters set to “PEAK_CUTOFF_PVALUE = 0.05, PEAK_CUTOFF_FDR = NA, FRAGMENT_LENGTH = 200”. Differential m6A peaks were also identified using the exomePeak R package with the same parameters. The Guitar R package (v1.16.0) was used to visualize genomic features related to m6A-RNA. For *de novo* motif analysis, identified m6A peaks with a *P* value <0.05 were selected and analyzed using homer (v4.10.4) with the “-len 6 -rna” parameters.

Gene expression levels were measured with featurecounts (v1.6.3) and differential expression analysis was conducted using the DESeq2 R package. Genes that exhibited differential expression between the two conditions were identified using a fold change cutoff of 1.5 and a significance threshold of *P* < 0.05.

### 2.12 MeRIP-qPCR

Total RNA was extracted from the cardiac tissue of mice using Trizol (Thermo Fisher Scientific). Subsequently, 100 μg of the extracted RNA was added to 500 μl of MeRIP buffer. The RNA sample was then incubated with an anti-N6-methyladenosine antibody (ab151230) along with 1 μl of rabbit IgG. This incubation was carried out to selectively pull down the m6A-modified EFEMP1. Finally, the m6A—bound RNA was detected and quantified using RT-qPCR.

### 2.13 Go and KEGG analyses

Genes and mRNAs that exhibited differential methylation, with a *P*-value <0.05 and a fold change >1.5, were chosen for subsequent analysis using Gene Ontology (GO) and Kyoto Encyclopedia of Genes and Genomes (KEGG) methods. All analyses were conducted using version 3.6.0 of the cluster profile R package. We acquired the enrichment analysis maps from the online data analysis website (http://www.bioinformatics.com.cn/).

### 2.14 Gene Set enrichment analysis

To identify biologically relevant pathways that were differentially expressed between two groups of samples, we performed gene set enrichment analysis (GSEA) using the M8 gene set, which contains cell type signature gene sets, as the reference. We considered a pathway to be significant if it had a *P* value <0.05 and a false discovery rate (FDR) <0.25, as described in the results section.

### 2.15 Statistical analyses

The mean ± standard deviation (SD) is used to represent the experimental data. Statistical analysis between two groups was performed using an unpaired two-tailed *t*-test. When comparing three groups of data, one-way analysis of variance (ANOVA) was utilized. The significance level for all these statistical analyses was set at *P* < 0.05.

## 3 Results

### 3.1 The m6A levels and related regulator expression patterns in young and aged hearts

**Figure 1:**
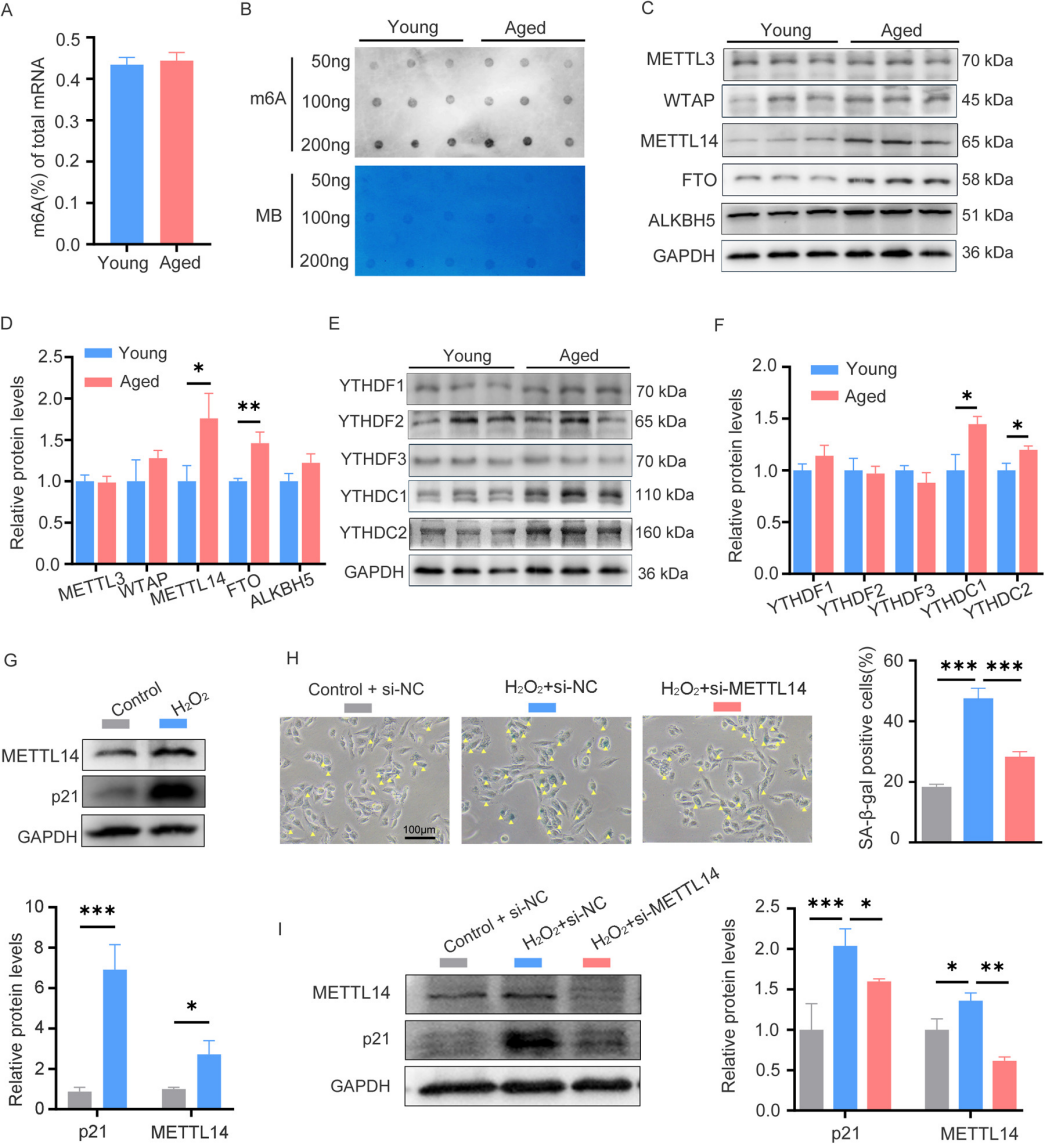
The m6A levels and related regulator expression patterns in young and aged hearts. **(A)** Quantification of m6A in mRNA in heart tissue from young and aged mice, *n* = 9 mice per group. **(B)** Global m6A level in mRNA of young and aged heart tissues was shown as three randomly selected samples by dot blot assay, Corresponding RNAs are loaded equally by a two-fold serial dilution with 50, 100, and 200 ng. MB, methyl blue, *n* = 9 mice per group. (**C,D**) Western blot analysis on young and aged heart protein lysates measuring expression of m6A methylase (METTL3, WTAP, and METTL14), m6A demethylases (ALKBH5 and FTO) and the corresponding densitometric analyses. *n* = 3 mice per group. (**E,F**) Western blot analysis on young and aged heart protein lysates measuring expression of m6A readers (YTHDC1/C2, YTHDF1/F2/F3) and the corresponding densitometric analyses, *n* = 3 mice per group. **(G)** The expression of p21 and METTL14 in AC16 cells treated with H_2_O_2_ was detected by western blotting. **(H)** AC16 cells were transfected with control or METTL14 siRNA for 24 h, followed by H_2_O_2_ treatment; then SA-β-gal staining was performed. Positive cells are shown in blue and marked by yellow arrows. **(I)** AC16 cells were treated as described in **(H)**, then protein levels of p21 and METTL14 were detected by western blotting. GAPDH was detected as the loading control. Data are presented as the mean ± SD of three independent experiments for **(G–I)**. *P < 0.05, **P < 0.01, ***P < 0.001 by one-way ANOVA or Student's *t*-test, as appropriate.

To explore the role of m6A RNA methylation in cardiac aging, we utilized C57BL/6 mice at young (6–7 weeks) and advanced (26–27 months) ages. The aged group exhibited hallmark characteristics of cardiac senescence, including higher SA-β-gal activity, upregulated expression of p16, p53, and γH2A.X, along with increased myocardial fibrosis (11, 12). We then compared m6A RNA methylation levels in young and aged hearts through colorimetry and m6A dot blot analysis; however, there were no differences in the global m6A content between the two groups (Figures 1A,B). Since the global m6A content did not show significant differences, we further investigated the expression levels of m6A regulatory proteins to explore potential dynamic changes in m6A modification. Notably, aged hearts demonstrated upregulated expression of the methyltransferase METTL14 and the demethyltransferase FTO, as well as increased expression of the readers YTHDC1 and YTHDC2 (Figures 1C–F). These findings suggest a potential role for m6A RNA methylation in cardiac aging and explain why global m6A RNA methylation levels remain balanced in the hearts of mice undergoing normal aging.

Cardiomyocytes constitute 30%–40% of the total cardiac cellular population (13). To study cardiac aging, the hydrogen peroxide (H_2_O_2_)-induced senescence model established using AC16 cells, an immortalized human cardiomyocyte cell line, is a well-established *in vitro* approach (14). When AC16 cells were treated with H_2_O_2_ at concentrations of 0, 50, 100, and 200 μM for 24 h, a dose-dependent senescence was induced. This was manifested by the progressive upregulation of p21 and an increased proportion of SA-β-gal positive cells (Supplementary Figures 2A,B). Consistent with the expression pattern of METTL14 in young and aged hearts, its expression was also upregulated in H_2_O_2_-induced senescent cells (Figure 1G). Functional studies showed that METTL14 knockdown alleviated H_2_O_2_-induced senescence phenotypes, as reflected by a reduction in the number of SA-β-gal positive cells and a decrease in p21 expression (Figures 1H,I). Collectively, these findings establish METTL14 as a key regulator of cardiomyocyte senescence, likely through m6A—mediated regulation of senescence—related pathways.

### 3.2 Overview of the m6A RNA methylation map in young and aged heart

**Figure 2:**
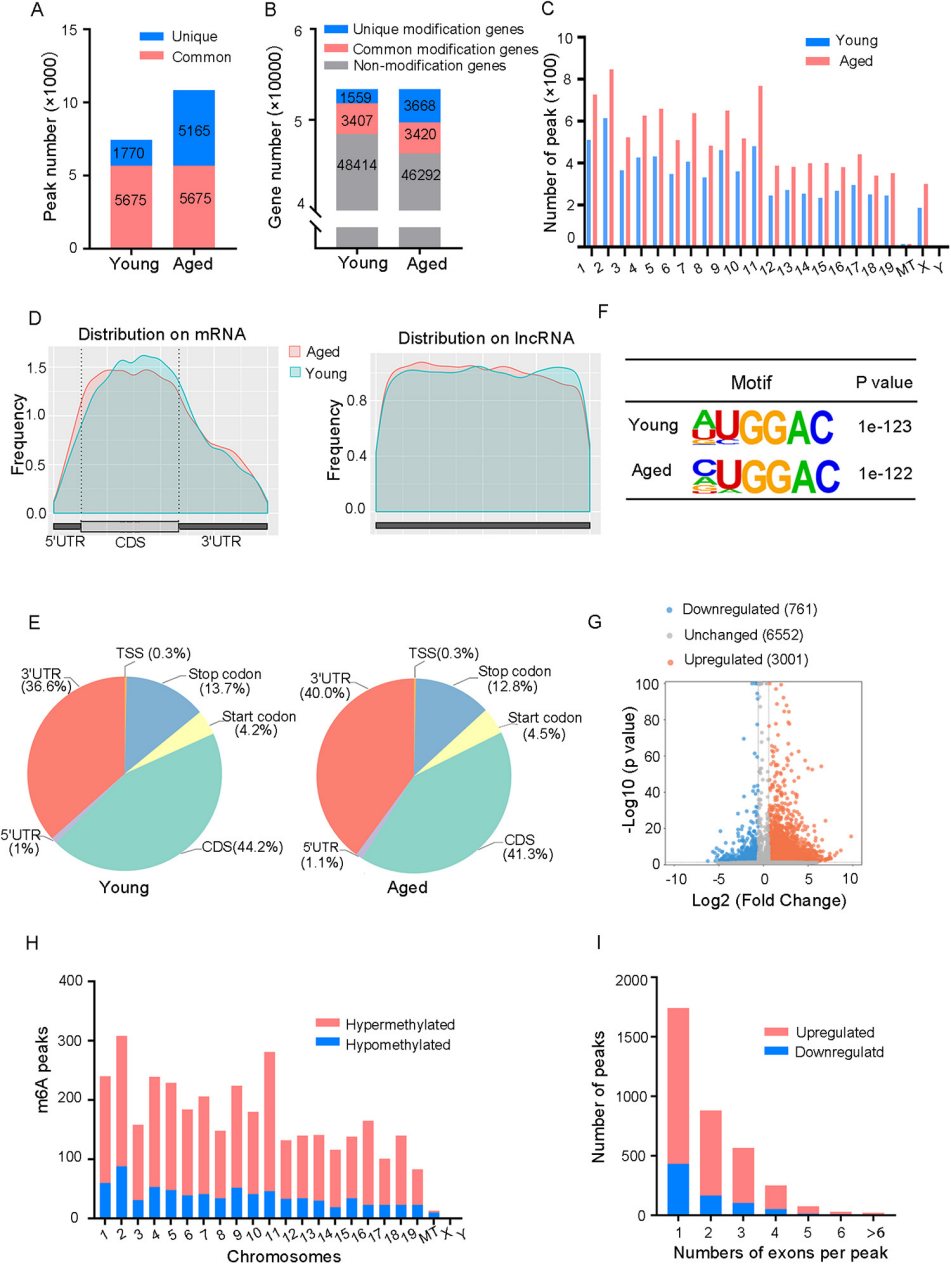
Overview of the m6A RNA methylation map in young and aged heart. **(A)** Number of m6A peaks identified in MeRIP-seq in young and aged mouse hearts. **(B)** Number of m6A-modified genes identified in MeRIP-seq. Common m6A genes contain at least one common m6A peak, while unique m6A genes contain no common m6A peaks. **(C)** The distribution patterns of m6A peaks in different chromosomes of young and aged mice. **(D)** Metagene plots showing the average distribution of m6A peaks identified across mRNA or lncRNA in the heart from young and aged mice. **(E)** Pie charts showing m6A peaks distribution in young and aged hearts. **(F)** Top consensus motif identified by HOMER with MeRIP-seq peaks in young and aged hearts. **(G)** Volcano plots showing significantly different m6A peaks. **(H)** The distribution of differentially methylated m6A peaks in mouse chromosomes. Fold change ≥1.5 and *P* < 0.05. **(I)** Distribution of the number of m6A peaks (*y* axis) was plotted based on the number of exons per peak (*x* axis) in young and aged hearts. *n* = 3 mice per group.

Methylated RNA immunoprecipitation sequencing (MeRIP-seq) was used to perform genome-wide profiling of methylation-modified mRNA and lncRNA in the hearts of young and aged mice. A total of 7,445 and 10,840 m6A peaks were detected in young and aged hearts, respectively, corresponding to 4,966 and 7,088 genes (Figures 2A,B). In both groups, chromosomes 1, 2, and 11 had the most m6A RNA methylation sites, and age did not significantly affect their methylation status (Figure 2C). Figure 2D indicated that methylation peaks were similarly distributed across coding sequences (CDS), 3′untranslated regions (3′UTRs), and long non-coding RNA (lncRNA) in both young and aged heart tissues. However, there were some differences in the pattern of methylation peaks between the two groups. The analysis of aged heart tissues revealed a higher proportion of methylation peaks in the 3′UTRs (40% vs. 36.6%), start codons (4.5% vs. 4.2%), and 5′UTRs (1.1% vs. 1%), while the proportion of peaks in the CDS was lower (41.3% vs. 44.2%) as well as in the stop codons (12.8% vs. 13.7%, Figure 2E). Moreover, we observed a significant enrichment of the GGAC motif within m6A sites in both young and aged heart tissues (Figure 2F).

In order to provide a clearer understanding of how m6A RNA methylation levels change with age, we conducted an analysis comparing the differences in m6A RNA methylation levels. A total of 3,762 m6A peaks showed significant alterations between two groups, with 3,001 peaks upregulated and 761 peaks downregulated in aged heart compared to the young heart (fold changes >1.5, *P* < 0.05, Figure 2G). The top 20 differently methylated m6A peaks are listed in Table 1. The altered m6A peaks were further found to be transcribed from all chromosomes, with the majority spanning one or two exons (Figures 2H,I).

**Table 1:** The top 20 differentially methylated m6A peaks (aged/young).

Gene name	Chromosome	Peak region	Peak start	Peak end	Regulation	Fold change	*P-*value
Snhg11	2	3′UTR	158383513	158383933	Up	897.64	2.51 × 10^−16^
Dennd4c	4	CDS	86836444	86837383	Up	404.50	1.29 × 10^−8^
Itpk1	12	3′UTR	102569747	102569898	Up	296.11	2.51 × 10^−7^
Gfpt1	6	5′UTR	87042905	87043056	Up	254.23	7.76 × 10^−6^
Ydjc	16	3′UTR	17160043	17160313	Up	187.40	3.63 × 10^−4^
Ube2l3	16	exon	17160055	17201488	Up	182.28	3.89 × 10^−4^
Slc2a3	6	exon	122739977	122742170	Up	171.25	8.13 × 10^−7^
Rpl9	5	CDS	65390725	65390906	Up	170.07	4.68 × 10^−5^
Dhx29	13	CDS	112940412	112941765	Up	170.07	4.68 × 10^−5^
Efemp1	11	5′UTR	28853233	28867662	Up	152.22	1.10 × 10^−3^
Ggps1	13	3′UTR	14053167	14053318	Down	76.11	1.02 × 10^−2^
Hnrnpd	5	3′UTR	99958265	99958446	Down	51.27	6.92 × 10^−8^
Ckb	12	exon	111671740	111671980	Down	43.71	3.24 × 10^−7^
Mfap1b	2	CDS	121466765	121469836	Down	41.93	7.76 × 10^−3^
Nsun2	13	CDS	69627630	69629658	Down	38.32	8.13 × 10^−10^
Rai2	X	CDS	161778427	161778606	Down	37.79	9.12 × 10^−5^
Ccdc50	16	3′UTR	27446398	27446639	Down	35.02	5.75 × 10^−6^
4930474H06Rik	12	exon	71318609	71318790	Down	31.12	5.89 × 10^−3^
Rps8	4	exon	117154643	117154792	Down	30.70	9.55 × 10^−9^
Mkrn2	6	3′UTR	115620288	115620469	Down	30.48	2.95 × 10^−3^

3′UTR, 3′ untranslated region; 5′UTR, 5′ untranslated region; CDS, coding sequence; exon, expressed region.

### 3.3 Differentially methylated mRNAs enriched in important signaling pathways

**Figure 3:**
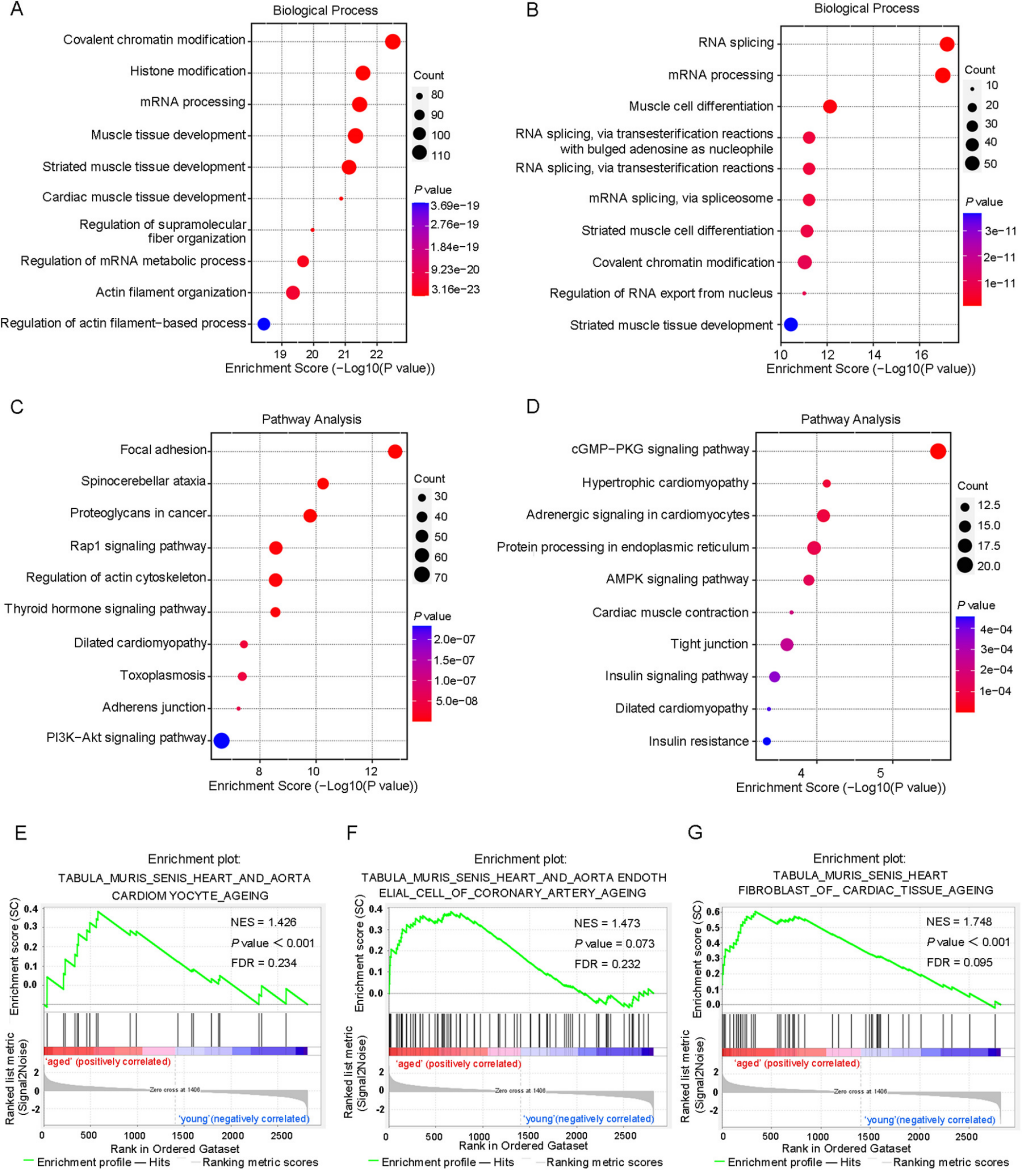
Differentially methylated mRNAs enriched in important signaling pathways. **(A)** The top 10 GO terms of genes with upregulated m6A peaks. **(B)** The top 10 GO terms of genes with downregulated m6A peaks. **(C)** The top 10 KEGG pathways of genes with upregulated m6A peaks. **(D)** The top 10 KEGG pathways of genes with downregulated m6A peaks. **(E–G)** GSEA enrichment of genes with significant changes in m6A RNA modification.

To study the biological significance of m6A RNA modification during cardiac aging, we performed GO, KEGG pathway, and GSEA function enrichment analyses on differentially methylated mRNAs. The GO enrichment analysis was divided into three classes: biological processes (BP), cellular components (CC), and molecular functions (MF). The GO analysis revealed that both up- and down-regulated methylation peaks were strongly linked with RNA fate and heart muscle tissue development (Figures 3A,B). More specifically, the BP analysis revealed a significant enrichment of hypermethylated peaks in mRNA processing, regulation of mRNA metabolic processes, and muscle tissue development (Figure 3A), whereas hypomethylated peaks were concentrated in RNA splicing, mRNA processing, regulation of RNA export from the nucleus, and muscle cell differentiation (Figure 3B). In the mRNA pathway analysis, the peaks with elevated methylation were strongly associated with dilated cardiomyopathy and the PI3K-Akt signaling pathway (Figure 3C). Furthermore, the most important biological pathways associated with the peaks with decreased methylation were hypertrophic cardiomyopathy, adrenergic signaling in cardiomyocytes, cardiac muscle contraction, and dilated cardiomyopathy signaling pathways (Figure 3D). Based on the above findings, we used GSEA to look into the underlying regulatory processes that contributed to cardiac aging. The analysis of our findings revealed that the genes exhibiting alterations in m6A RNA modification were enriched in various aspects of cardiac senescence. Particularly, these genes were found to be enriched in processes related to cardiac senescence, such as heart and aorta cardiomyocyte aging (Figure 3E), coronary artery aging (Figure 3F), and cardiac tissue aging (Figure 3G).

Interestingly, several of the enriched GO terms, such as chromatin remodeling, RNA splicing, and RNA export, also suggest the potential involvement of other epigenetic mechanisms beyond m6A methylation, including histone modifications and DNA methylation. These findings imply that m6A methylation may function in accordance with other epigenetic layers to coordinately regulate gene expression during cardiac aging.

### 3.4 Overview of the transcriptome profiles and a combined analysis of the MeRIP-Seq and RNA-Seq data

**Figure 4:**
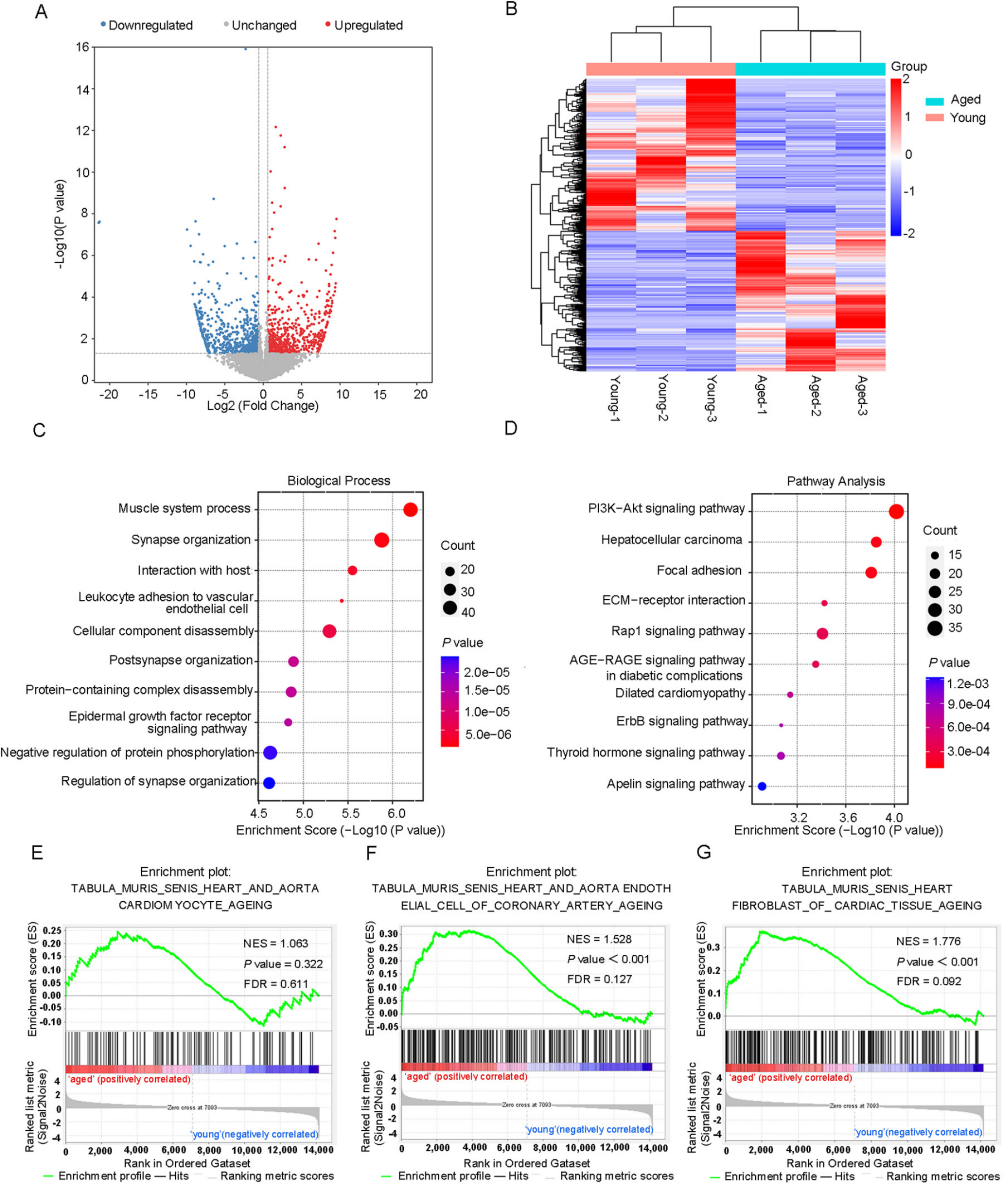
DEGs hierarchical clustering analysis and functional annotation. **(A)** Volcano plots and **(B)** heatmap plots showing differentially expressed genes in young and aged hearts. Fold change ≥1.5 and *P* < 0.05. **(C)** The top 10 GO terms of differentially expressed genes. **(D)** The top 10 KEGG pathways of differentially expressed genes. **(E)** GSEA enrichment of genes with significant changes in RNA levels.

An RNA-Seq study of cardiac tissues, with a sample size of three per group (*n* = 3 per group), identified 654 significantly upregulated genes and 709 downregulated genes (fold changes ≥1.5 and *P* < 0.05) in aged mice (Figures 4A,B). The top 20 differentially expressed genes were shown in Table 2. Furthermore, a comprehensive analysis of the DEGs was performed in both young and aged hearts using GO and KEGG pathways. GO analysis revealed DEGs were highly enriched in BP, particularly in the muscle system process and leukocyte adherence to vascular endothelial cells (Figure 4C). In terms of CC, the analysis demonstrated that the DEGs were significantly enriched in myofibril and sarcomere (Supplementary Figure 3A). Moreover, the analysis revealed that the DEGs were enriched in MF, which included the binding of cell adhesion molecules and protein tyrosine kinases (Supplementary Figure 3B). KEGG pathway analysis was used to identify the top ten pathways with the highest enrichment scores, which included the PI3K-Akt signaling pathway and dilated cardiomyopathy (Figure 4D). Lamc3, for instance, was involved in the PI3K-Akt signaling pathway, while Tpm3 and Itga10 were associated with dilated cardiomyopathy. We also conducted GSEA on all genes and discovered that certain gene expression patterns associated with cardiac aging were positively enriched in the old heart group, including aorta endothelial cell of coronary artery aging and heart fibroblast of cardiac tissue aging (Figures 4E–G).

**Table 2:** The top 20 differentially expressed genes (aged/young).

Gene name	Fold change	Regulation	*P*-value
Necab1	113.35	Up	6.29 × 10^−4^
Katnbl1	61.32	Up	1.54 × 10^−6^
Cd302	54.02	Up	2.50 × 10^−3^
Arc	53.63	Up	8.24 × 10^−3^
Tnfrsf23	53.00	Up	3.08 × 10^−3^
Rimbp2	51.51	Up	5.31 × 10^−5^
Tox2	50.97	Up	3.18 × 10^−2^
Stac2	47.65	Up	2.21 × 10^−2^
Zfp992	45.62	Up	6.76 × 10^−4^
Omg	38.47	Up	8.58 × 10^−3^
Paox	302.96	Down	2.09 × 10^−6^
Pycrl	92.68	Down	2.00 × 10^−6^
Fap	87.97	Down	1.92 × 10^−9^
Six5	85.97	Down	9.23 × 10^−5^
Btc	65.93	Down	5.42 × 10^−4^
Rem1	65.56	Down	1.67 × 10^−4^
Rpe	53.72	Down	3.75 × 10^−4^
Nrk	51.57	Down	5.79 × 10^−3^
Bax	51.06	Down	2.85 × 10^−5^
Lair1	49.47	Down	3.01 × 10^−3^

**Figure 5:**
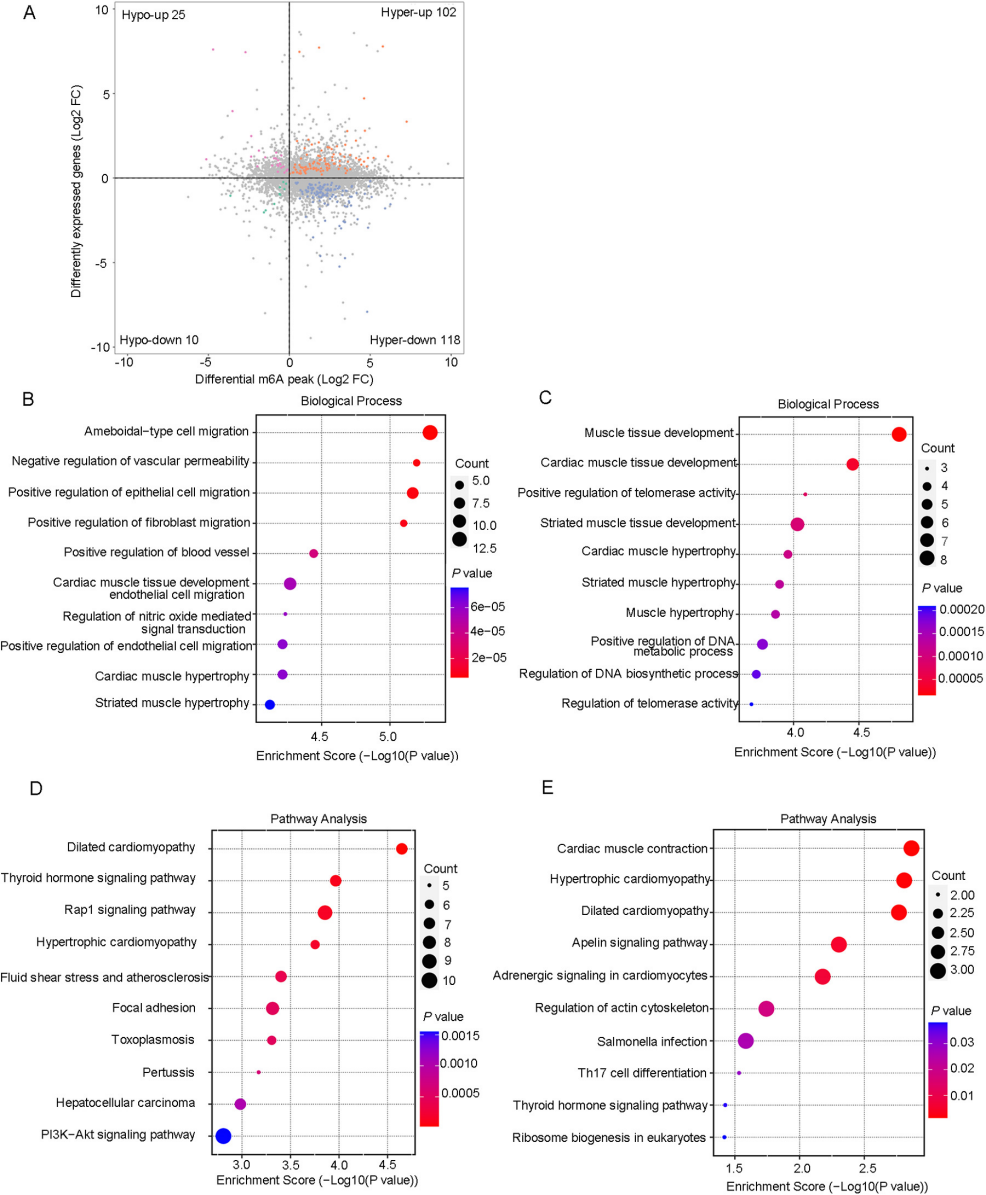
Conjoint analysis of the MeRIP-Seq and RNA-Seq data. **(A)** Four quadrant graphs of genes with differential m6A RNA methylation and differentially expressed mRNA levels. **(B)** The top 10 GO terms of genes with hypermethylated peaks and differentially expressed RNA levels. **(C)** The top 10 GO terms of genes with hypomethylated peaks and differentially expressed RNA levels. **(D)** The top 10 KEGG pathways of genes with hypermethylated peaks and differentially expressed RNA levels. **(E)** The top 10 KEGG pathways of genes with hypomethylated peaks and differentially expressed RNA levels.

After a comprehensive analysis of the MeRIP-seq and RNA-seq data, all differentially methylated m6A peaks with varying mRNA levels were organized into four distinct groups. We detected 220 DEGs displaying hypermethylated m6A peaks, of which 102 were significantly upregulated (hyper-up) and 118 were downregulated (hyper-down). Additionally, 35 DEGs exhibited hypomethylated m6A peaks, with 25 upregulated (hypo-up) genes and 10 downregulated (hypo-down) genes (Figure 5A). To determine whether cardiac aging-related process or pathways were involved, we conducted GO and KEGG pathway analyses of DEGs with m6A hypermethylation or hypomethylation. Consequently, we found that biological processes related to cardiac aging were highly enriched in the top 10 GO processes for DEGs with m6A hyper- or hypo-methylation (Figures 5B,C). Specifically, DEGs with both m6A hypermethylation and hypomethylation were enriched in cardiac muscle tissue development, cardiac muscle hypertrophy, and striated muscle hypertrophy simultaneously (Figures 5B,C). Additionally, the DEGs with m6A hypermethylation were specifically enriched in the negative regulation of vascular permeability and positive regulation of endothelial cell migration, while DEGs with m6A hypomethylation were specifically enriched in the regulation of telomerase activity (Figures 5B,C). Furthermore, KEGG pathway analysis revealed a significant enrichment of DEGs with both hyper- and hypomethylated states in the pathways of dilated cardiomyopathy and hypertrophic cardiomyopathy, which are two prevalent cardiac diseases commonly associated with aging (Figures 5D,E).

### 3.5 The candidate gene EFEMP1 containing m6A modification correlates with cardiac aging

**Figure 6:**
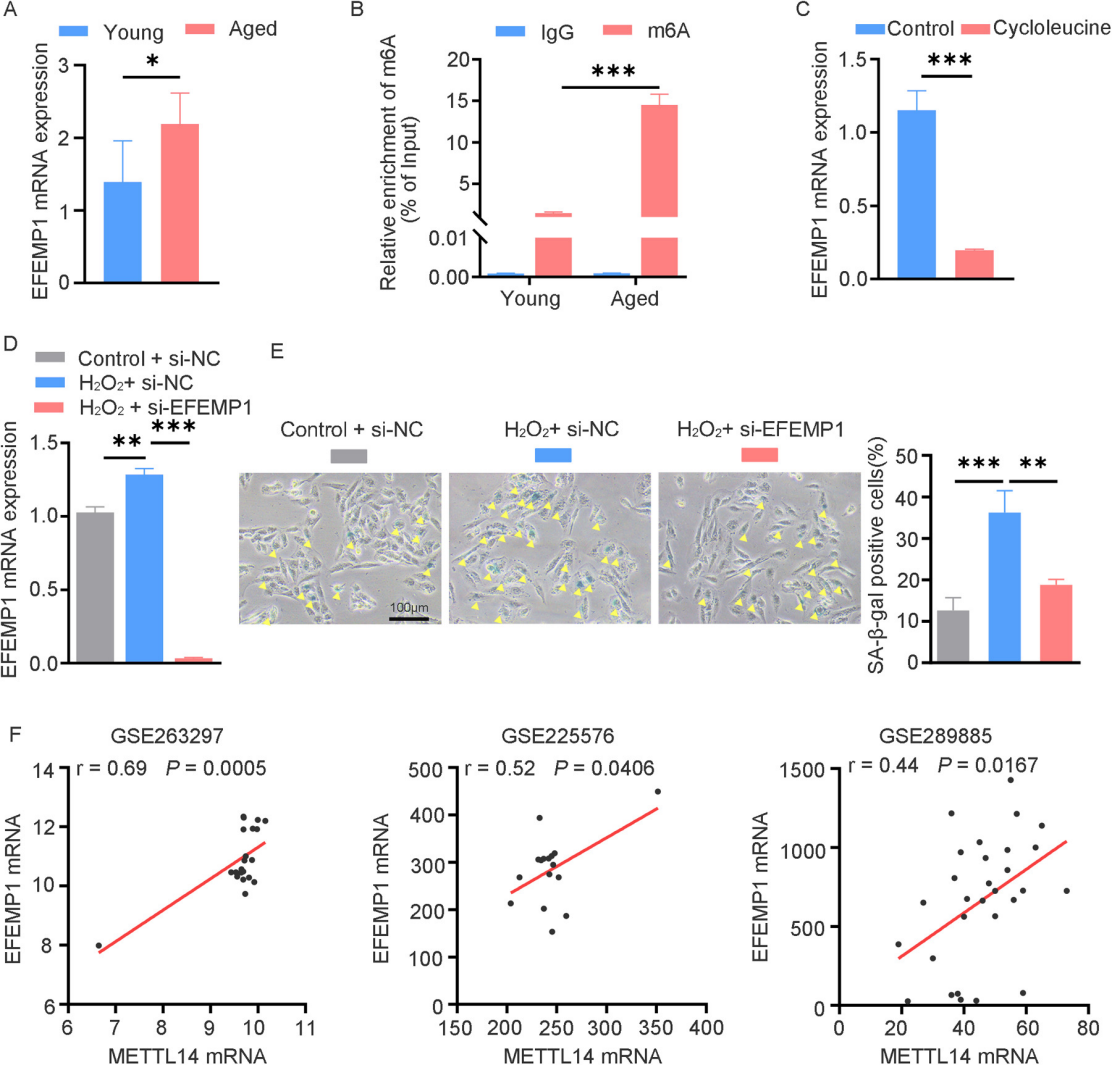
The candidate gene EFEMP1 containing m6A modification correlates with cardiac aging. **(A)** qRT-PCR analysis of EFEMP1 mRNA expression in young and aged mouse hearts. *n* = 5 mice per group. **(B)** MeRIP-qPCR analysis of m6A enrichment of EFEMP1 in young and aged mouse hearts. *n* = 3 mice per group. **(C)** EFEMP1 mRNA expression changes in AC16 cells treated with 100 μM cycloleucine for 24 h. **(D)** AC16 cells transfected with control or EFEMP1 siRNA for 24 h, followed by H_2_O_2_ treatment; EFEMP1 mRNA expression was measured by qRT-PCR. **(E)** SA-β-gal staining in AC16 cells treated as described in **(D)** Positive cells are shown in blue and marked by yellow arrows. **(F)** Positive correlation between METTL14 and EFEMP1 mRNA expression in multiple cardiac datasets (GSE263297, *n* = 21; GSE225576, *n* = 16; GSE289885, *n* = 29). Data are presented as the mean ± SD of three independent experiments for **(C–E)**. *P < 0.05, **P < 0.01, ***P < 0.001 by one-way ANOVA or Student's *t*-test, as appropriate.

To explore the functional significance of m6A-modified genes in cardiac aging, we identified EFEMP1 as a key candidate. Consistent with the mRNA-seq and MeRIP-seq results, subsequent RT-qPCR and MeRIP-qPCR analyses confirmed a significant upregulation of EFEMP1 expression, accompanied by enhanced m6A modification of EFEMP1 mRNA in aged mouse hearts (Figures 6A,B). To determine the m6A-dependent regulation of EFEMP1, we treated AC16 cells with the methyltransferase inhibitor cycloleucine at a concentration of 100 μM. This treatment led to a significant reduction in EFEMP1 mRNA levels, indicating that m6A modification positively regulates EFEMP1 expression (Figure 6C). Furthermore, our investigation demonstrated that H_2_O_2_-induced oxidative stress significantly increased EFEMP1 expression in AC16 cells (Figure 6D). Notably, knockdown of EFEMP1 effectively alleviated cellular senescence, as indicated by a decrease in the number of SA-β-gal positive cells following H_2_O_2_ treatment (Figure 6E). Moreover, to further explore the relationship between EFEMP1 and METTL14, we comprehensively analyzed multiple public datasets, including patients with ischemic cardiomyopathy (GSE263297) and young and aged mouse hearts (GSE225576 and GSE289885). The analysis clearly showed a positive correlation between the mRNA levels of METTL14 and EFEMP1 (Figure 6F). These findings strongly suggest that METTL14 likely plays a role in regulating the methylation modification level of EFEMP1. This potential regulation may have a significant impact on the complex molecular mechanisms underlying cardiac aging.

## 4 Discussion

m6A RNA methylation, a heritable and reversible chemical modification, is involved in epigenetic regulation, and has been associated with various pathological processes, including cardiac diseases (15, 16). Aging, which underlies the decline in cardiac physiological function, is the primary risk factor for the development of cardiac diseases. To prevent age-related cardiac diseases, further insights should be gained from investigating the processes involved in cardiac aging. This study provided the first insight into the m6A landscape of cardiac aging in young and aged mice. We compared the global methylation levels and the expression of major m6A regulators in young and aged mice. Using MeRIP-seq analysis, we identified a total of 3,762 aberrant m6A peaks, with 3,001 m6A peaks upregulated and 761 m6A peaks downregulated. Further investigation using GO, KEGG pathway, and GSEA uncovered potential roles of differentially methylated transcripts. Moreover, by integrating MeRIP-seq and RNA-seq data, we identified 255 genes with differentially methylated m6A peaks and simultaneously different expression, which were enriched in signaling pathways associated with cardiac aging. These findings suggest that m6A RNA modification may play a fundamental role in regulating the processes of cardiac aging.

In this study, we found no significant difference in global m6A levels between young and aged hearts, which is consistent with a previous report (17). However, the expression levels of METTL14 and FTO were simultaneously upregulated in aged hearts, which may be the reason why the global m6A level was unchanged. Previous research has indicated that both METTL14 and FTO are significant in the context of cardiovascular diseases. In their investigation, Pang et al. discovered that the protein METTL14 exhibited a significant increase in hearts exposed to ischemia-reperfusion (I/R) and in cardiomyocytes undergoing injury induced by oxidative stress (18). The downregulation of METTL14 contributed to alleviation of acute myocardial I/R injury and cardiac dysfunction during I/R remodeling (16). Jian et al. found that METTL14 was upregulated in atherosclerotic lesions, and knockout of METTL14 significantly inhibited endothelial inflammation as well as the development of atherosclerosis (19). Additionally, Guo et al. reported that METTL14 levels are significantly altered in patients with coronary heart disease and are associated with disease severity and clinical prognosis (20). Similar to these findings, our current study reveals that METTL14 is also upregulated in H_2_O_2_-induced senescent AC16 cardiomyocytes. This upregulation further emphasizes the crucial role of METTL14 in various types of cardiac stress-related responses, including oxidative stress-induced senescence. Moreover, Qian et al. demonstrated the role of METTL14 in ovarian aging, suggesting that METTL14 participates in regulating aging—related signaling pathways via m6A modification (21). This implies that the function of METTL14 in the aging process may be universal. It is noteworthy that FTO was discovered to be downregulated in failing mammalian hearts, and it exhibited a protective function during ischemia by selectively removing methylation from transcripts associated with cardiac contraction (22). A recent study reported that FTO overexpression attenuated the hypoxia/reoxygenation (H/R)-induced apoptosis and inflammation of cardiomyocytes (23). Additionally, Su et al. reported a significant decrease in FTO expression in aged mouse hearts subjected to I/R injury (17). The difference in FTO expression between the present study and the prior one may be attributed to differences in physiological and pathological conditions. The homozygous mutations in FTO lead to left ventricular hypertrophy, which supported that FTO played an important role in early development of cardiovascular systems (24). The role of FTO-dependent m6A demethylation in the process of cardiac aging has not been explored, and this is an area that we plan to investigate in greater detail in our future research.

To further map the m6A RNA modification status, we performed MeRIP-seq and RNA-seq on hearts from young and aged mice. During our MeRIP-seq analysis, we identified 7,445 distinct m6A peaks in the young heart and 10,840 m6A peaks in the aged heart. Further analysis identified 3,762 anomalously regulated m6A peaks, with 3,001 upregulated and 761 downregulated. In the RNA-seq analysis, a total of 1,363 genes exhibiting differential expression were identified. To investigate the biological functions of these genes with differentially methylated m6A peaks or differential RNA levels, we conducted GO and KEGG analyses. Our analysis revealed that genes with dysregulated m6A peaks were primarily associated with RNA metabolic processes, including mRNA processing, RNA splicing, and regulation of RNA export from the nucleus. Additionally, these genes were found to be involved in the development and differentiation of muscle tissue. Consistent with previous reports, m6A have been proposed to be involved in various key regulatory processes of RNA metabolism (25, 26). In addition, the differential m6A genes were closely related to the Rap1, AMPK, PI3K-Akt, cGMP-PKG, dilated cardiomyopathy, hypertrophic cardiomyopathy, and adrenergic signaling in cardiomyocytes signaling pathways. Cai et al. found that loss of Rap1 could accelerate cardiac aging via modulating the p53/PPAR*α* signaling pathway (27). Previous studies have shown that myocardial AMPK activity decreased in aged hearts, and AMPK inactivation led to myocardial autophagy dysfunction (28–31). AMPK signaling can activate PI3K-Akt signaling pathway, and then protect the myocardium from ischemia-reperfusion (I-R) injury (32). It has been reported that the activation of the cGMP—PKG signaling pathway can cause an increase in the antioxidant response and reductions in ER stress, and improved cardiac function in mice with age-induced cardiac dysfunction (33). Beyond m6A RNA methylation, other epigenetic modifications, including DNA methylation and histone modifications, also play critical roles in regulating gene expression during cardiac aging. Emerging evidence suggests a complex interplay among these epigenetic layers. For instance, DNA methylation patterns dynamically shift with age and have been associated with transcriptional changes and functional decline in cardiac tissues (34). Histone modifications, such as H3K27me3 and H3K9ac, are also altered in aged hearts and contribute to chromatin remodeling and senescence-associated gene expression (35, 36). These modifications may function in concert with m6A methylation to fine-tune the transcriptional landscape, influencing key processes such as inflammation, oxidative stress responses, and endothelial dysfunction. Therefore, integrating multi-omics analyses that encompass m6A methylation, DNA methylation, and histone modifications could provide a more comprehensive understanding of the epigenetic regulation underlying cardiac aging.

Through a comprehensive analysis of MeRIP-seq and RNA-seq data, we identified 255 genes with differentially methylated m6A peaks and concomitant differential mRNA expression during the process of cardiac aging. To evaluate the biological significance of these genes, enrichment analyses of GO and KEGG pathways were conducted. In the KEGG analysis, two pathways were found to be enriched and related to cardiac diseases, specifically dilated cardiomyopathy and hypertrophic cardiomyopathy. Of note, these two pathways were also notably enriched in the KEGG enrichment analysis of genes with dysregulated m6A peaks. Another significant pathway affected in this study was the fluid shear stress and atherosclerosis. The shear stress of arterial blood flow at the endothelium has long been known to influence atherosclerosis, which is a major pathogenic factor in cardiovascular diseases (37). During further investigations, four (Slc37a3, Gm20300, Hmox1, and Pank1) of the top nine hypermethylated genes were downregulated, five (Mical2, Efemp1, Mapk10, Bcorl1, and Gm43808) were upregulated, and just one gene (Ccdc50) was hypomethylated and upregulated in the aged heart (Table 3). Some of these genes are known to play important roles in the development of cardiac diseases (38, 39). For instance, Gabor et al. investigated the cardioprotection achieved through gene delivery of hypoxia inducible factor-1a depending on the downstream factor HMOX-1 activity (38). Deng et al. identified that specific knockdown of MAPK10 in the heart directly reverses hyperglycemia-induced myocardial dysfunction (39). EFEMP1, also known as fibulin -3, has emerged as a molecule of interest in the cardiac aging process based on our current findings. In aged hearts, EFEMP1 exhibits hyper-methylated m6A peaks and significant mRNA upregulation. To clarify the m6A-dependent regulation of EFEMP1, treating of AC16 cells with cycloleucine significantly reduced EFEMP1 mRNA levels, indicating that m6A modification positively regulates EFEMP1 expression. Moreover, H_2_O_2_-induced oxidative stress can markedly increase EFEMP1 expression, and knocking down EFEMP1 effectively alleviates cellular senescence. In other pathological conditions, EFEMP1 is known to regulate the extracellular matrix (ECM) (40). Alterations in the ECM can lead to cardiac remodeling in heart diseases (41). Our results align with previous human cohort studies reporting an association between EFEMP1 and brain aging and dementia (42), suggesting that EFEMP1 may play a more widespread role in the aging process across diverse organs. The positive correlation between METTL14 and EFEMP1 mRNA levels, as observed in multiple cardiac datasets, implies a potential regulatory relationship between these two molecules. Given that METTL14 is a methyltransferase, this correlation strongly suggests that METTL14 may modulate EFEMP1 expression through m6A-mediated mechanisms. Evidently, these m6A-modified genes and their influence on cardiac aging are likely of great significance. However, to fully understand their roles, further research is essential to elucidate the precise mechanisms underlying these genes in cardiac aging.

**Table 3:** The top 10 differently expressed genes containing differently methylated peaks (aged/young).

Gene name	Chromosome	Peak region	Peak start	Peak end	Fold change	Regulation	*P*-value
Efemp1	11	5′UTR	28853233	28867662	152.22	Hyper-up	1.10 × 10^−3^
Slc37a3	6	3′UTR	39336057	39336267	76.11	Hyper-down	1.45 × 10^−5^
Mical2	7	CDS	112318468	112318589	69.07	Hyper-up	4.79 × 10^−3^
Gm20300	10	exon	30604899	30605079	60.55	Hyper-down	9.12 × 10^−8^
Gm43808	5	exon	30870618	30870798	54.95	Hyper-up	5.25 × 10^−4^
Mapk10	5	5′UTR	103211033	103211184	37.01	Hyper-up	5.75 × 10^−3^
Pank1	19	3′UTR	34811637	34811817	34.06	Hyper-down	1.17 × 10^−3^
Hmox1	8	CDS	75097163	75098944	29.24	Hyper-down	4.57 × 10^−4^
Bcorl1	X	CDS	48370948	48375089	29.04	Hyper-up	3.09 × 10^−2^
Ccdc50	16	3′UTR	27446398	27446639	35.02	Hypo-up	5.75 × 10^−6^

3′UTR, 3′ untranslated region; 5′UTR, 5′ untranslated region; CDS, coding sequence; exon, expressed region.

To summarize, we constructed an m6A transcriptome map of young and aged hearts, and performed comprehensive bioinformatics analyses to investigate the potential connection between m6A RNA modification and mRNA expression during cardiac aging. Further research on m6A target genes in cardiac aging might contribute to the clinical application of the targeted therapy for age-related cardiac diseases.

## Data Availability

The original contributions presented in the study are publicly available. This data can be found here: raw sequencing reads were deposited to the GEO database, with the accession number GSE298965.
