# Supplementary figures and images for: N6-methyladenosine RNA landscape in the aged mouse hearts
Source: Front Cardiovasc Med. 2025 Jun 18;12:1563364. doi: 10.3389/fcvm.2025.1563364 (PMC12213823; doi:10.3389/fcvm.2025.1563364)

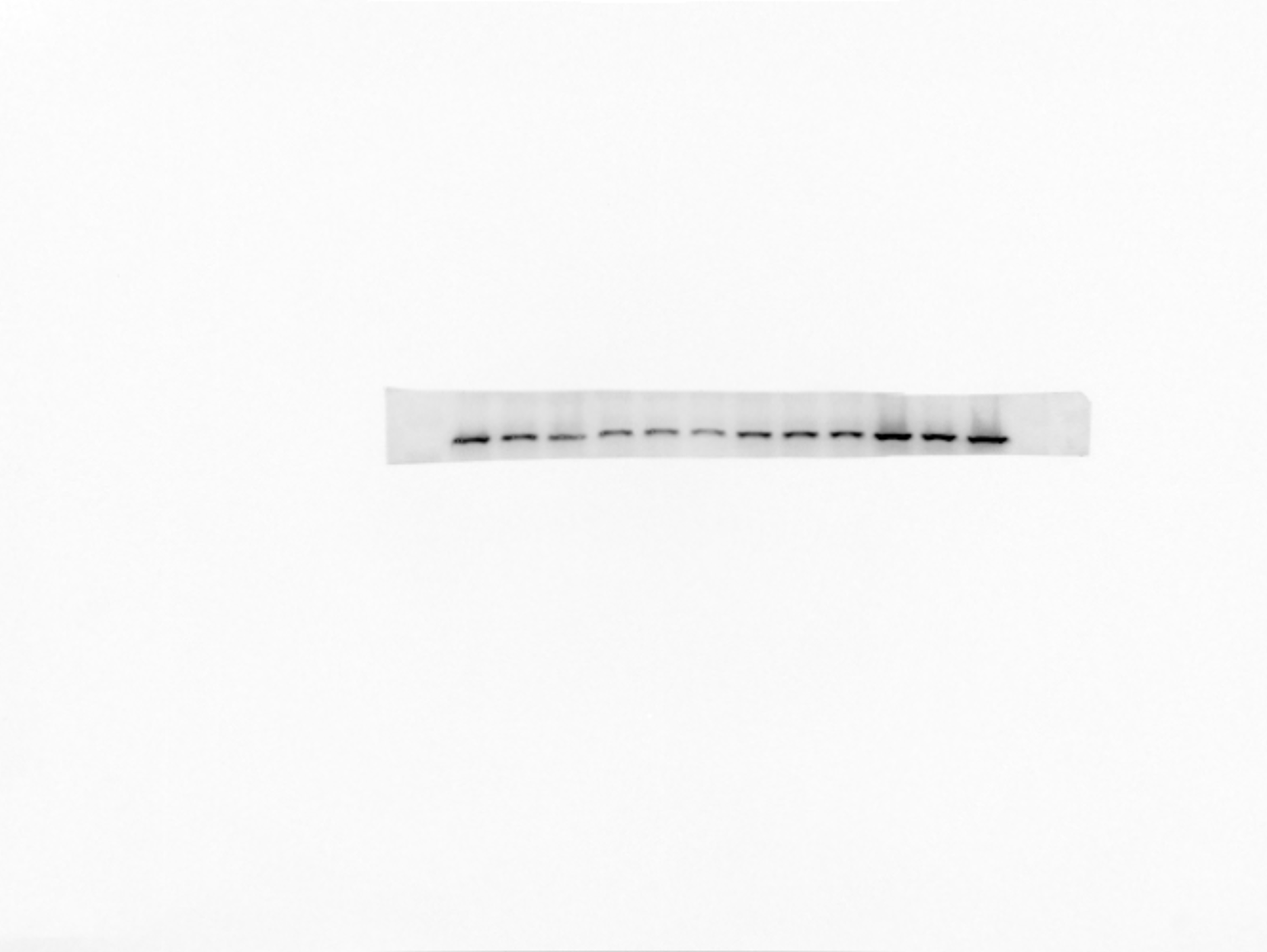

Supplement: Supplementary file 1 [file Datasheet1.zip › original raw files of Figure1/Figure 1C/membrane1-FTO.tif]

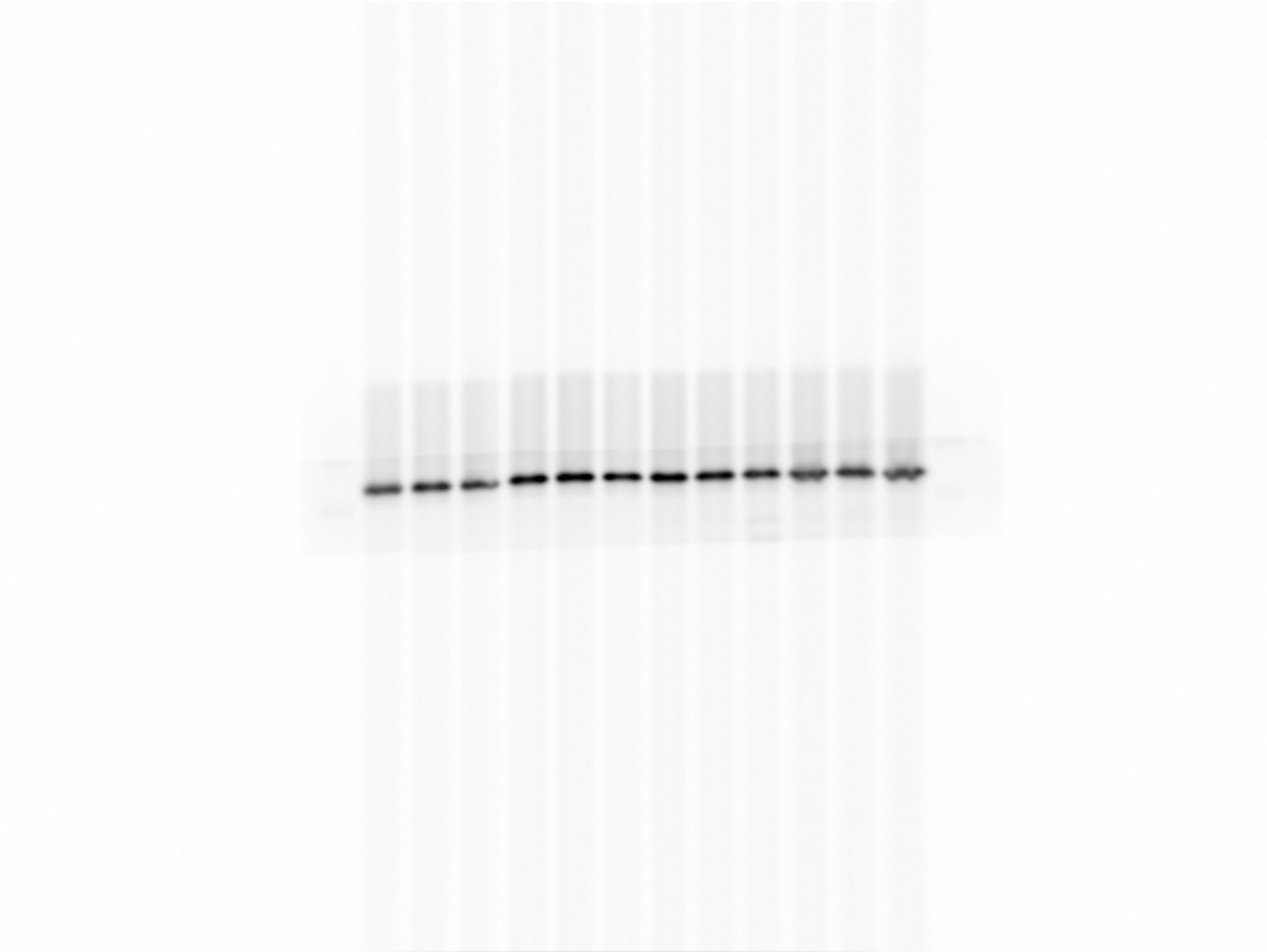

Supplement: Supplementary file 1 [file Datasheet1.zip › original raw files of Figure1/Figure 1C/membrane1-GAPDH.tif]

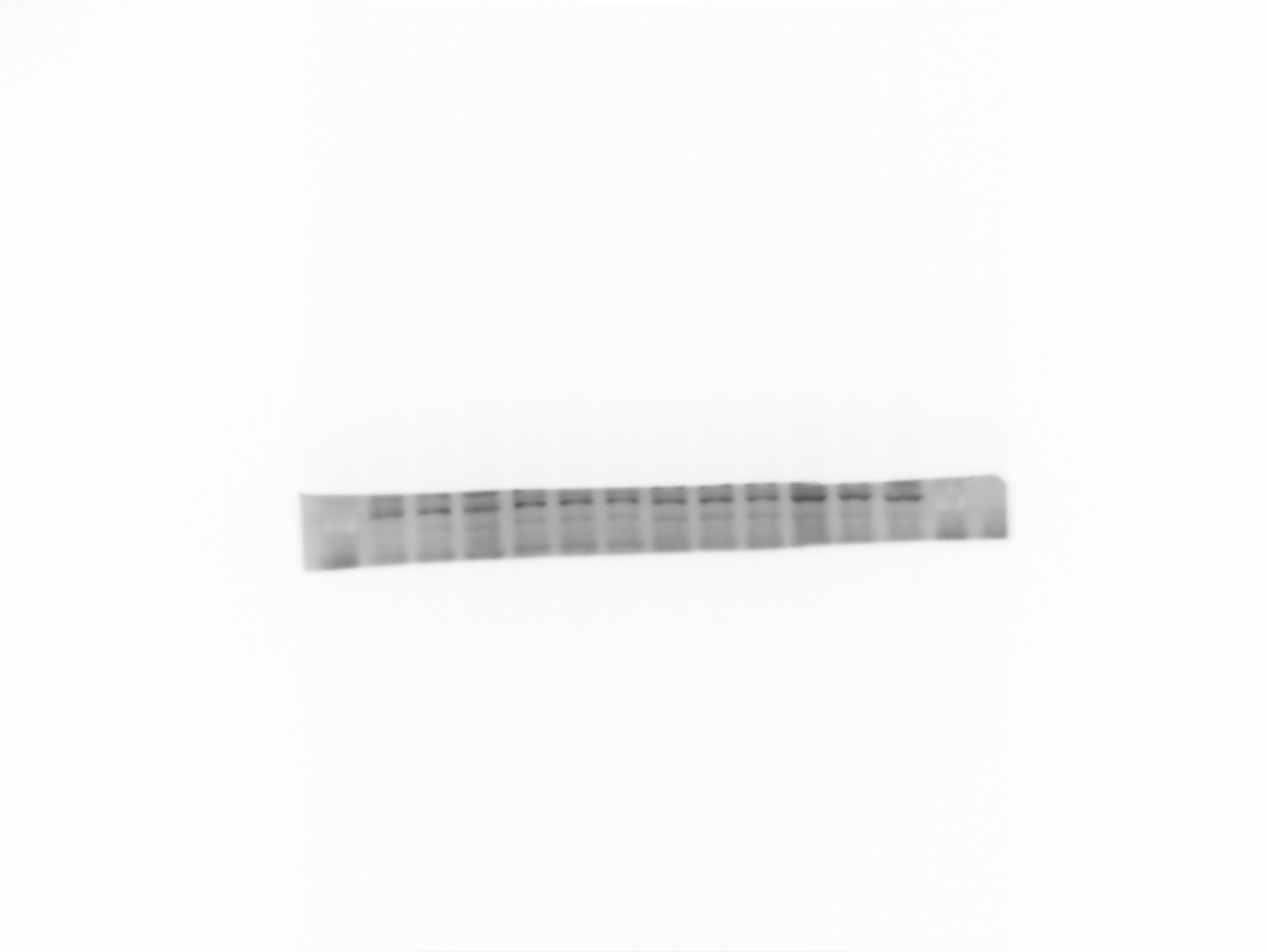

Supplement: Supplementary file 1 [file Datasheet1.zip › original raw files of Figure1/Figure 1C/membrane1-METTL3.tif]

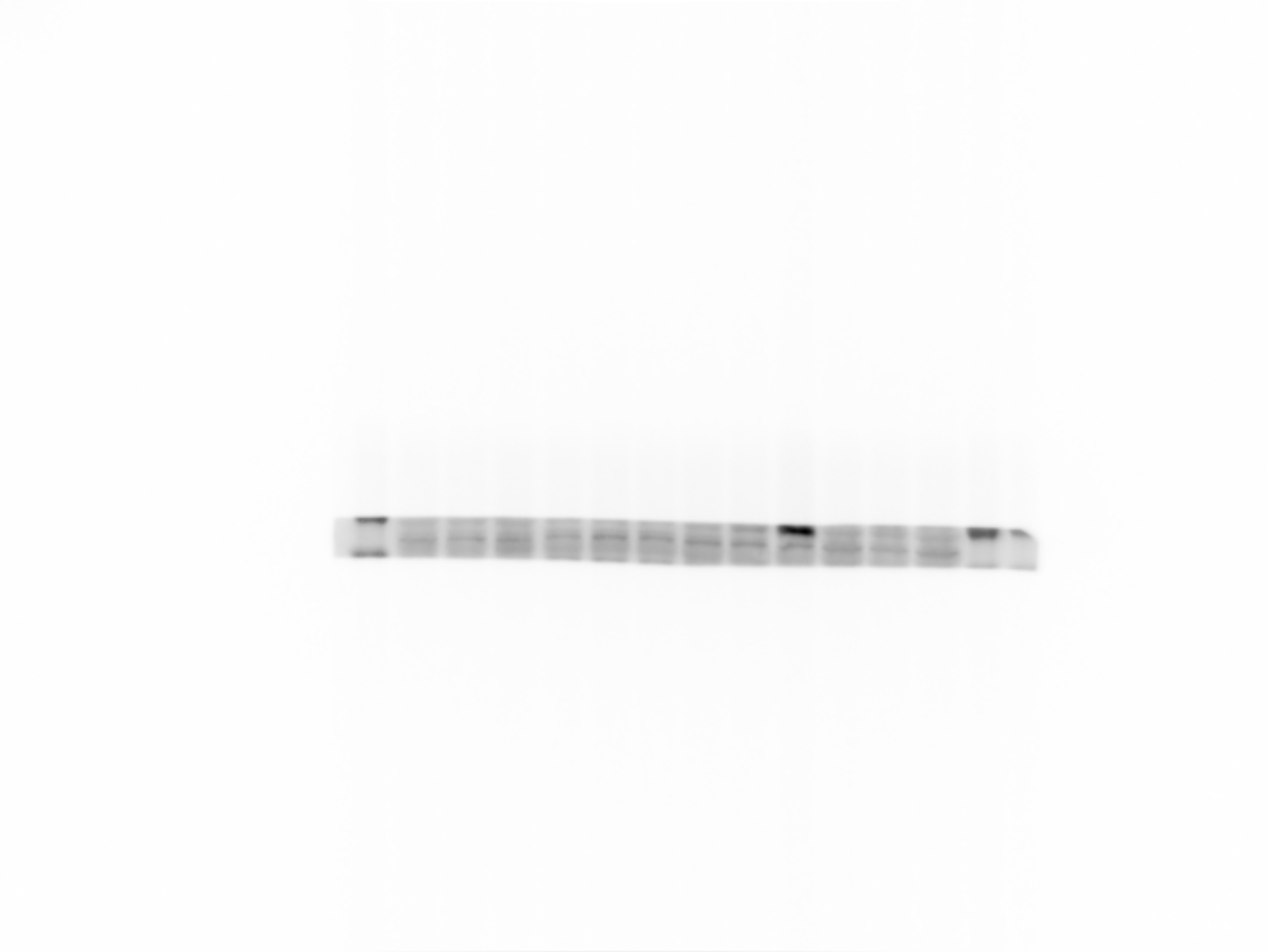

Supplement: Supplementary file 1 [file Datasheet1.zip › original raw files of Figure1/Figure 1C/membrane1-WTAP.tif]

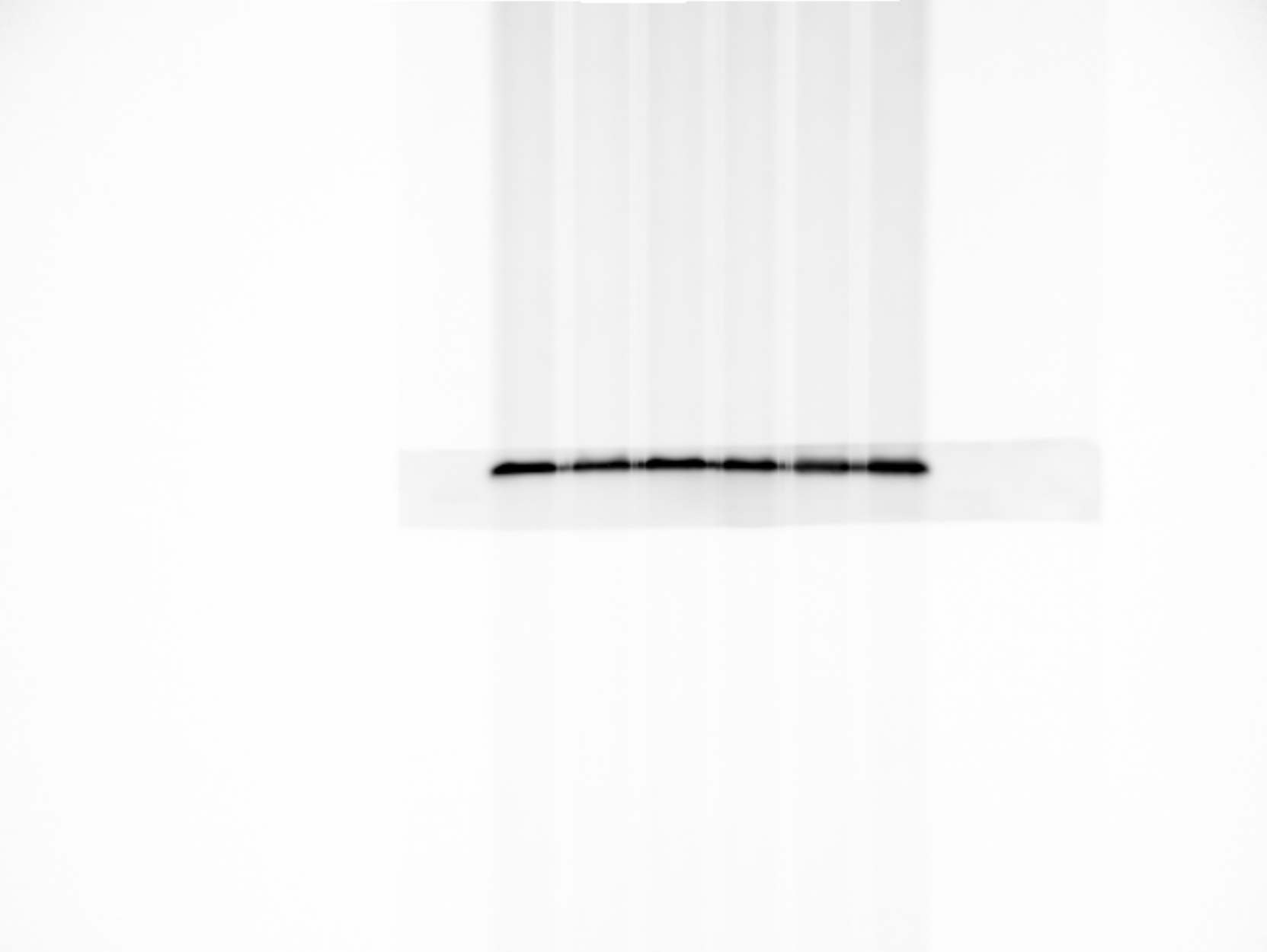

Supplement: Supplementary file 1 [file Datasheet1.zip › original raw files of Figure1/Figure 1C/membrane2-GAPDH.tif]

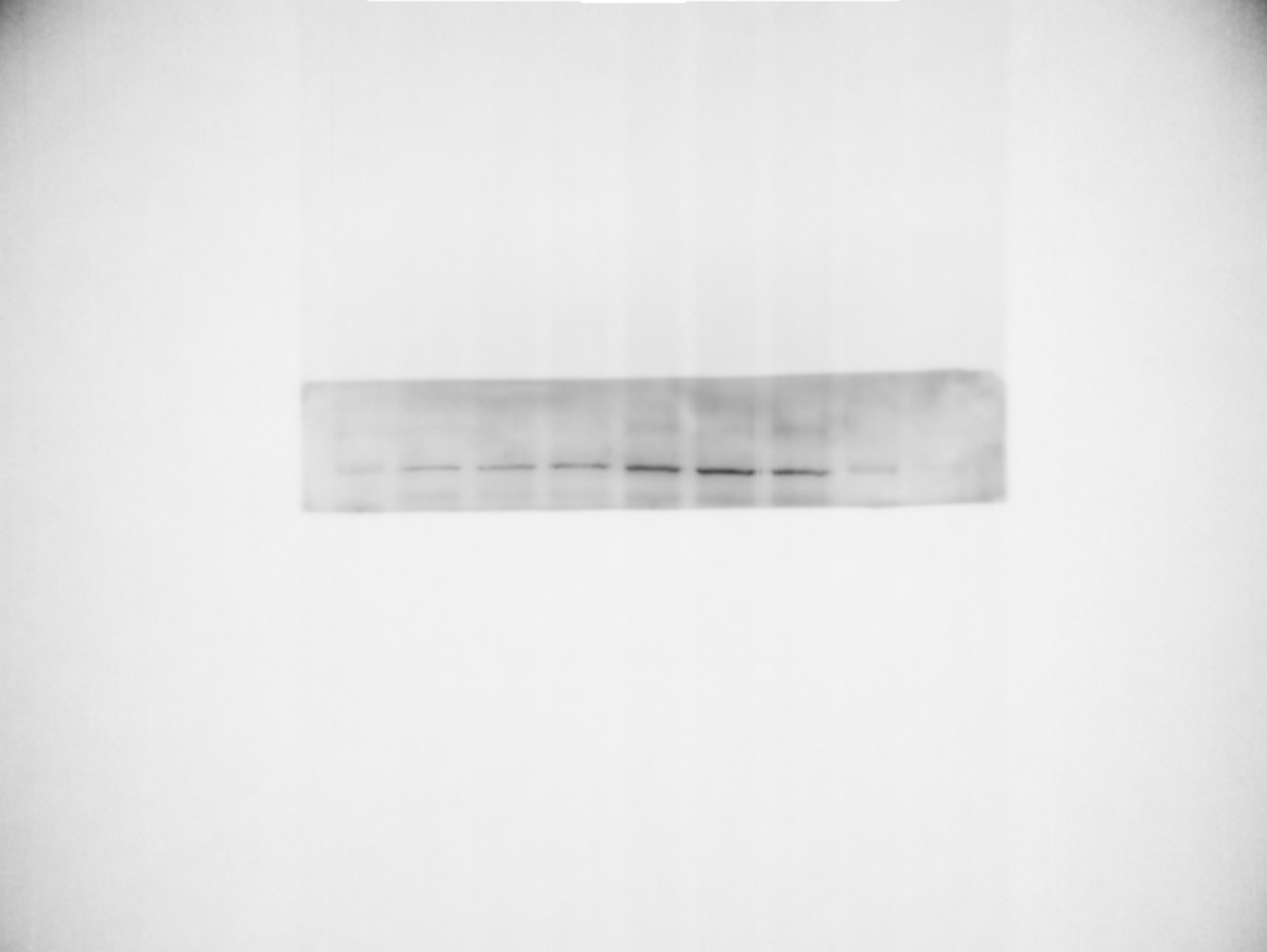

Supplement: Supplementary file 1 [file Datasheet1.zip › original raw files of Figure1/Figure 1C/membrane2-METTL14.tif]

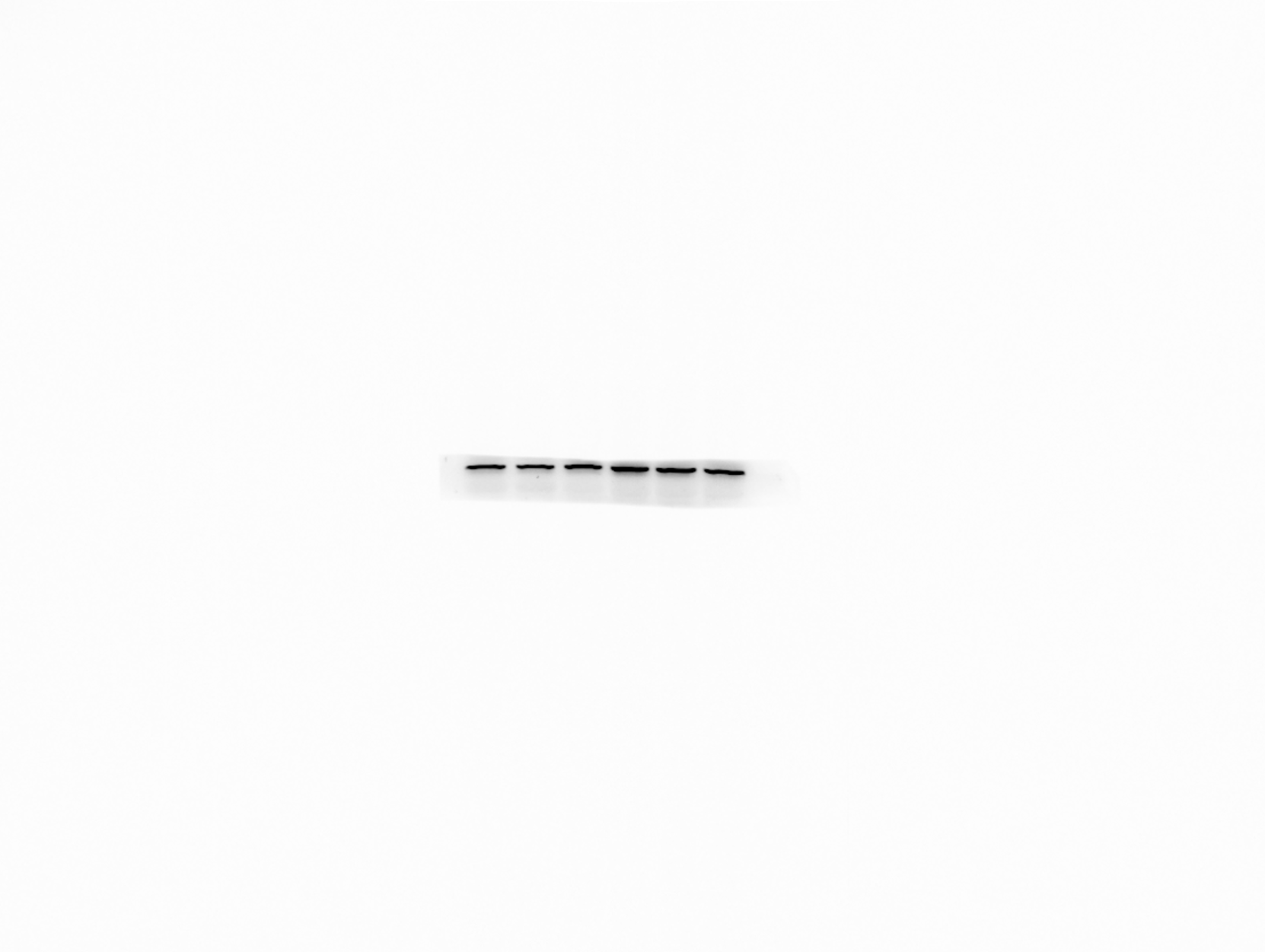

Supplement: Supplementary file 1 [file Datasheet1.zip › original raw files of Figure1/Figure 1C/membrane3-ALKBH5.tif]

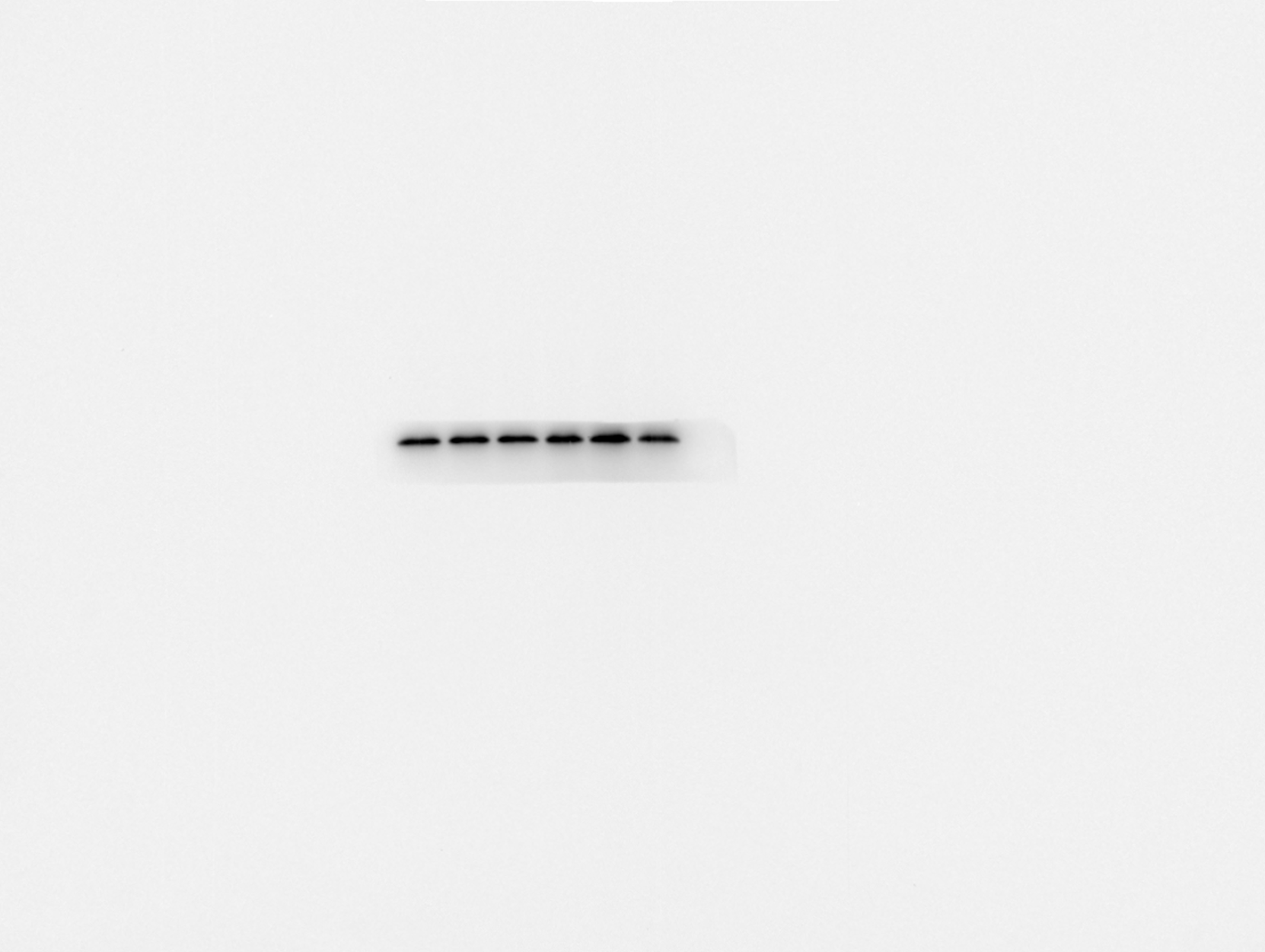

Supplement: Supplementary file 1 [file Datasheet1.zip › original raw files of Figure1/Figure 1C/membrane3-GAPDH.tif]

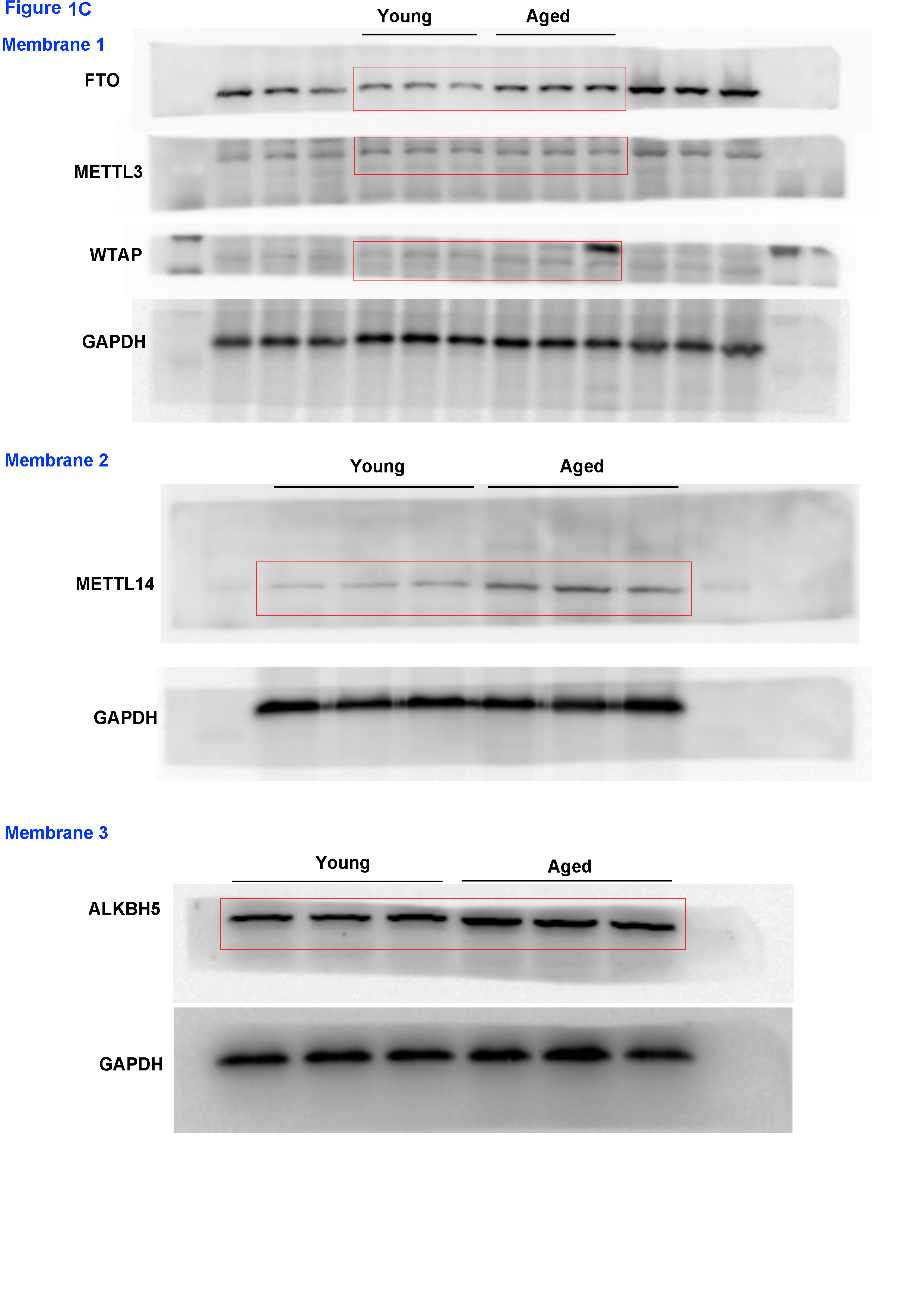

Supplement: Supplementary file 1 [file Datasheet1.zip › original raw files of Figure1/Figure 1C/Integration and annotation of the original images.tif]

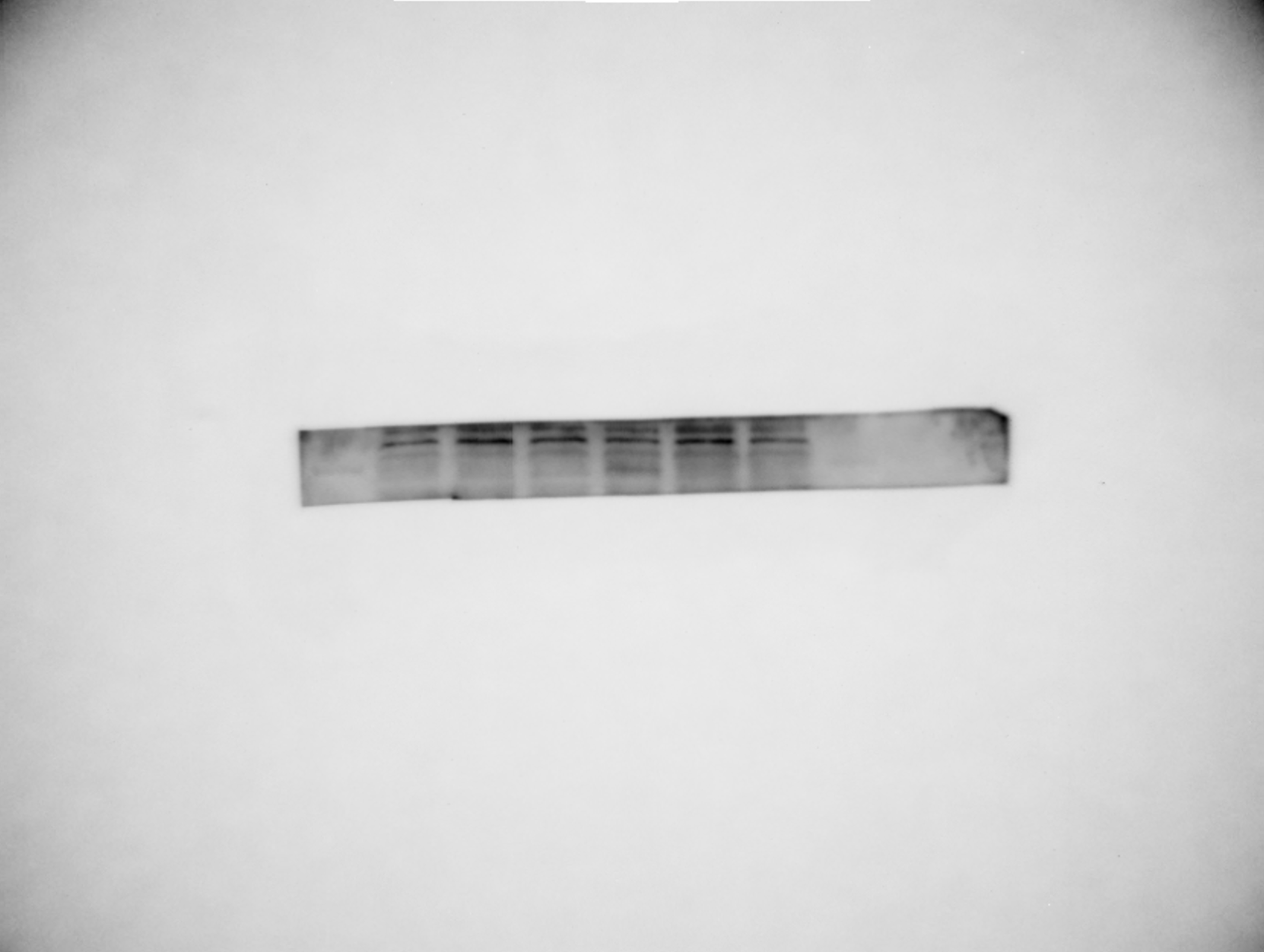

Supplement: Supplementary file 1 [file Datasheet1.zip › original raw files of Figure1/Figure 1E/membrane4-YTHDF2 .tif]

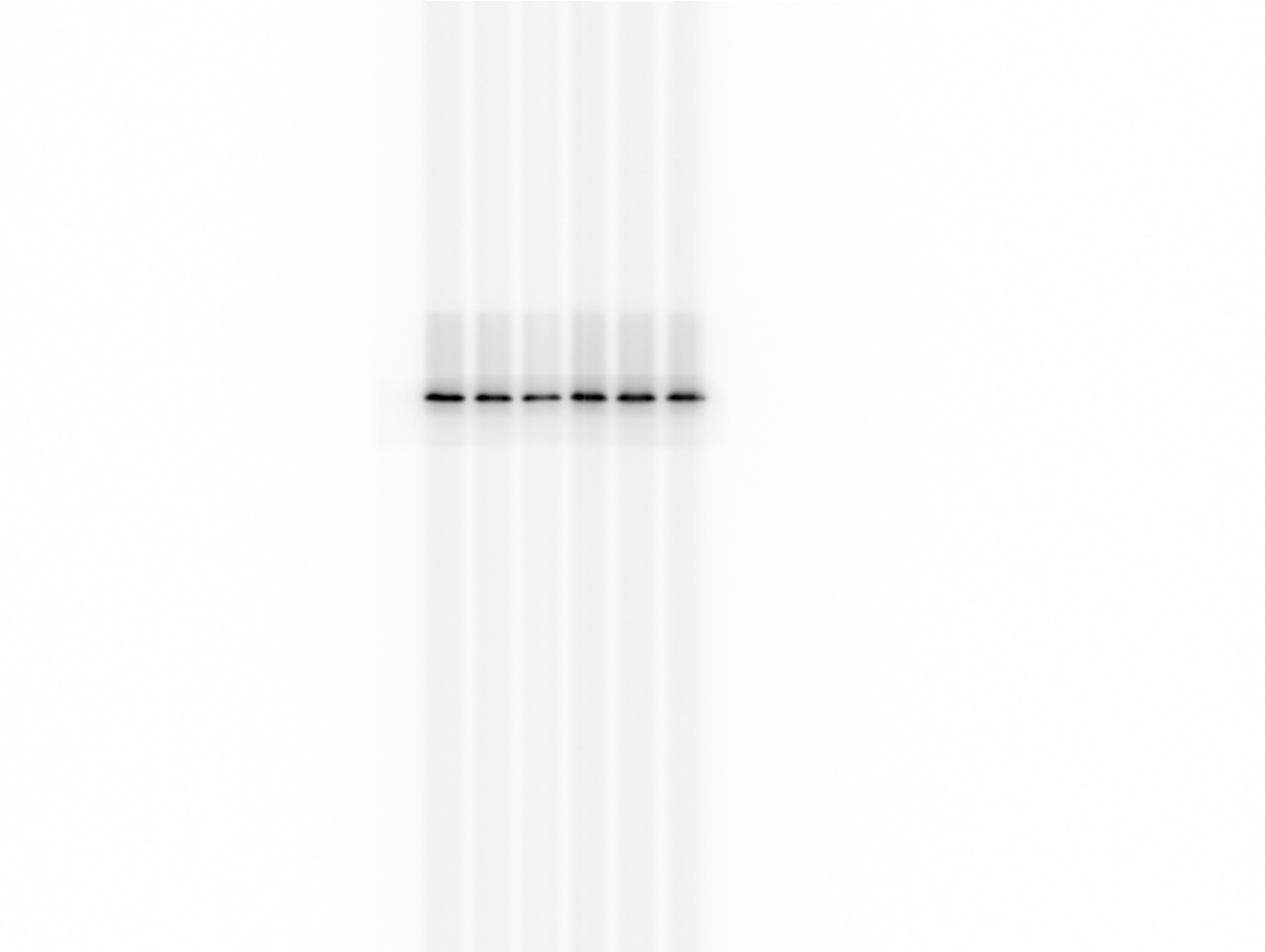

Supplement: Supplementary file 1 [file Datasheet1.zip › original raw files of Figure1/Figure 1E/membrane1-GAPDH.tif]

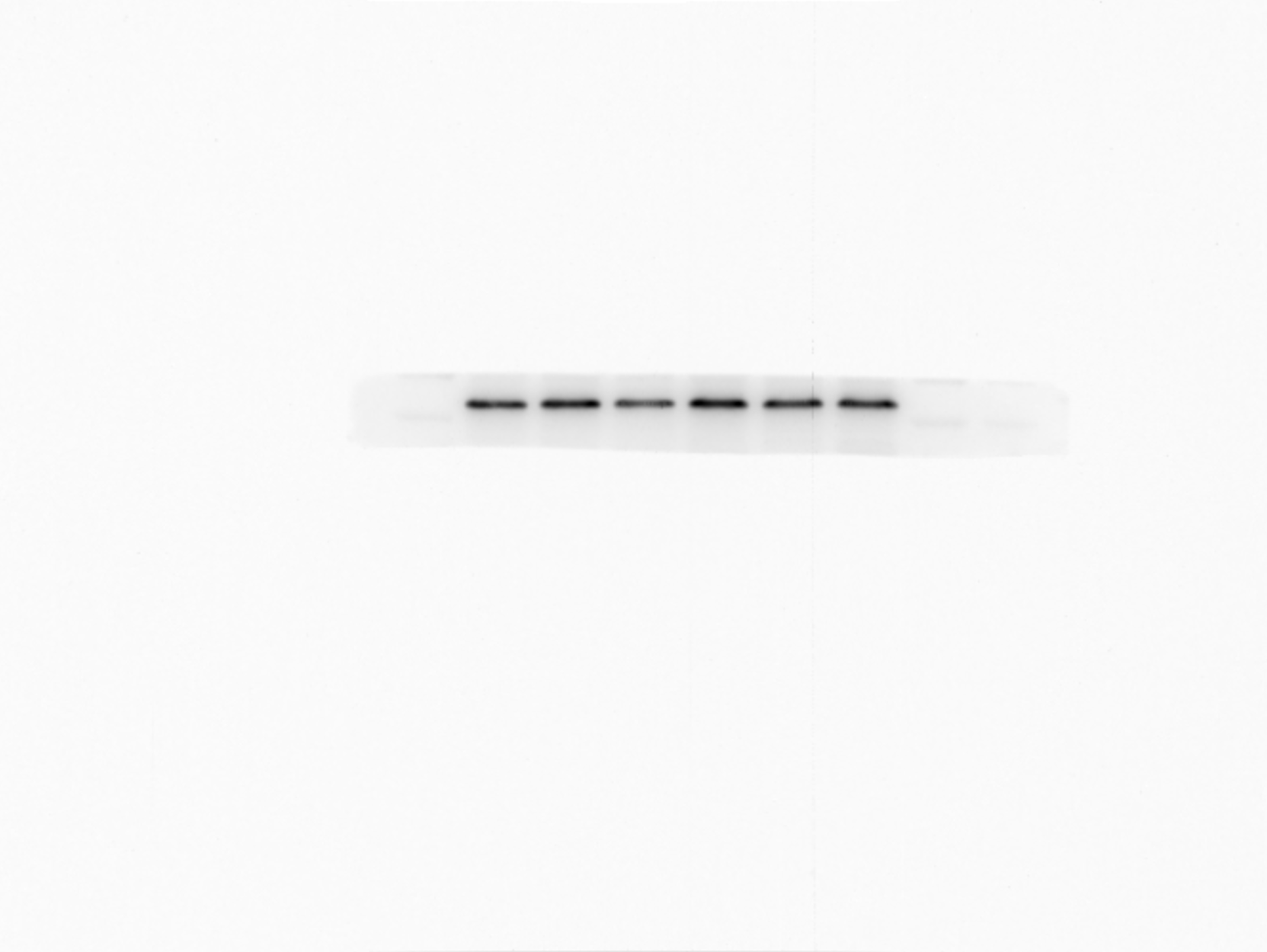

Supplement: Supplementary file 1 [file Datasheet1.zip › original raw files of Figure1/Figure 1E/membrane2-GAPDH.tif]

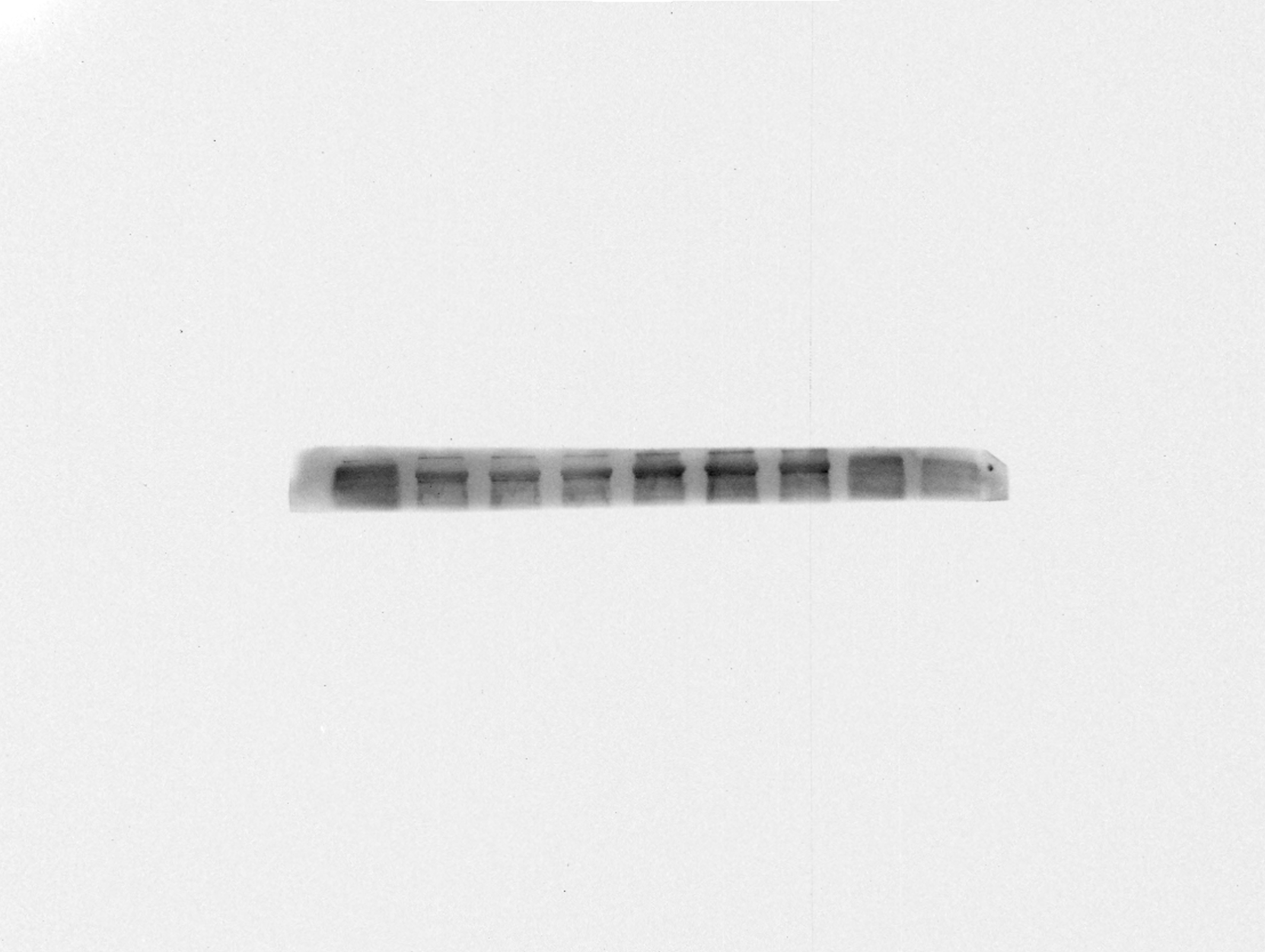

Supplement: Supplementary file 1 [file Datasheet1.zip › original raw files of Figure1/Figure 1E/membrane2-YTHDC2.tif]

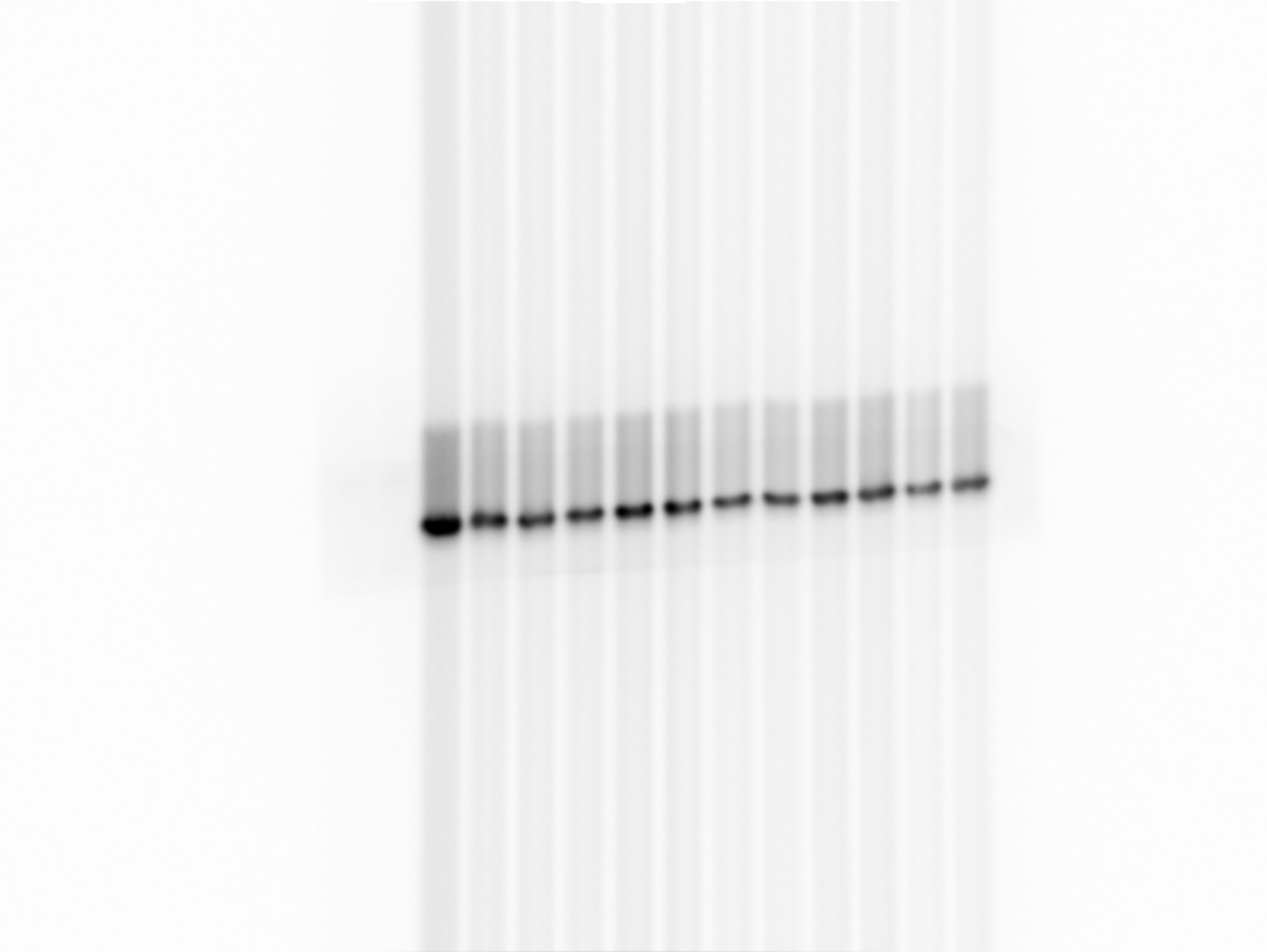

Supplement: Supplementary file 1 [file Datasheet1.zip › original raw files of Figure1/Figure 1E/membrane3-GAPDH.tif]

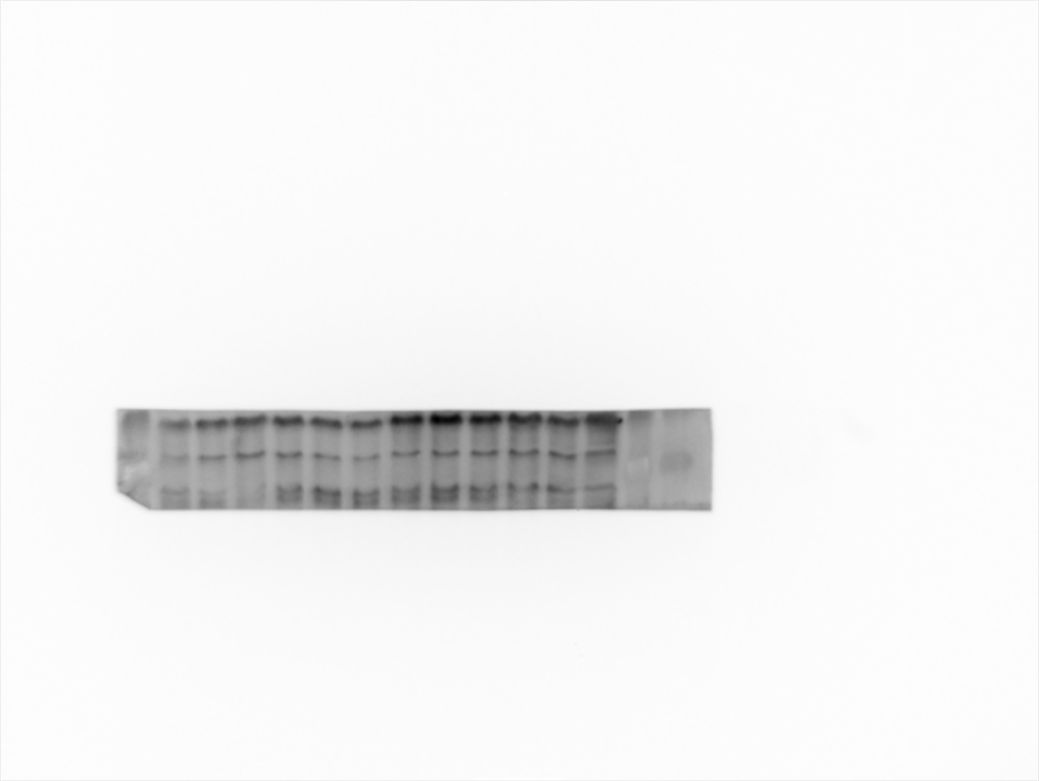

Supplement: Supplementary file 1 [file Datasheet1.zip › original raw files of Figure1/Figure 1E/membrane3-YTHDF1.tif]

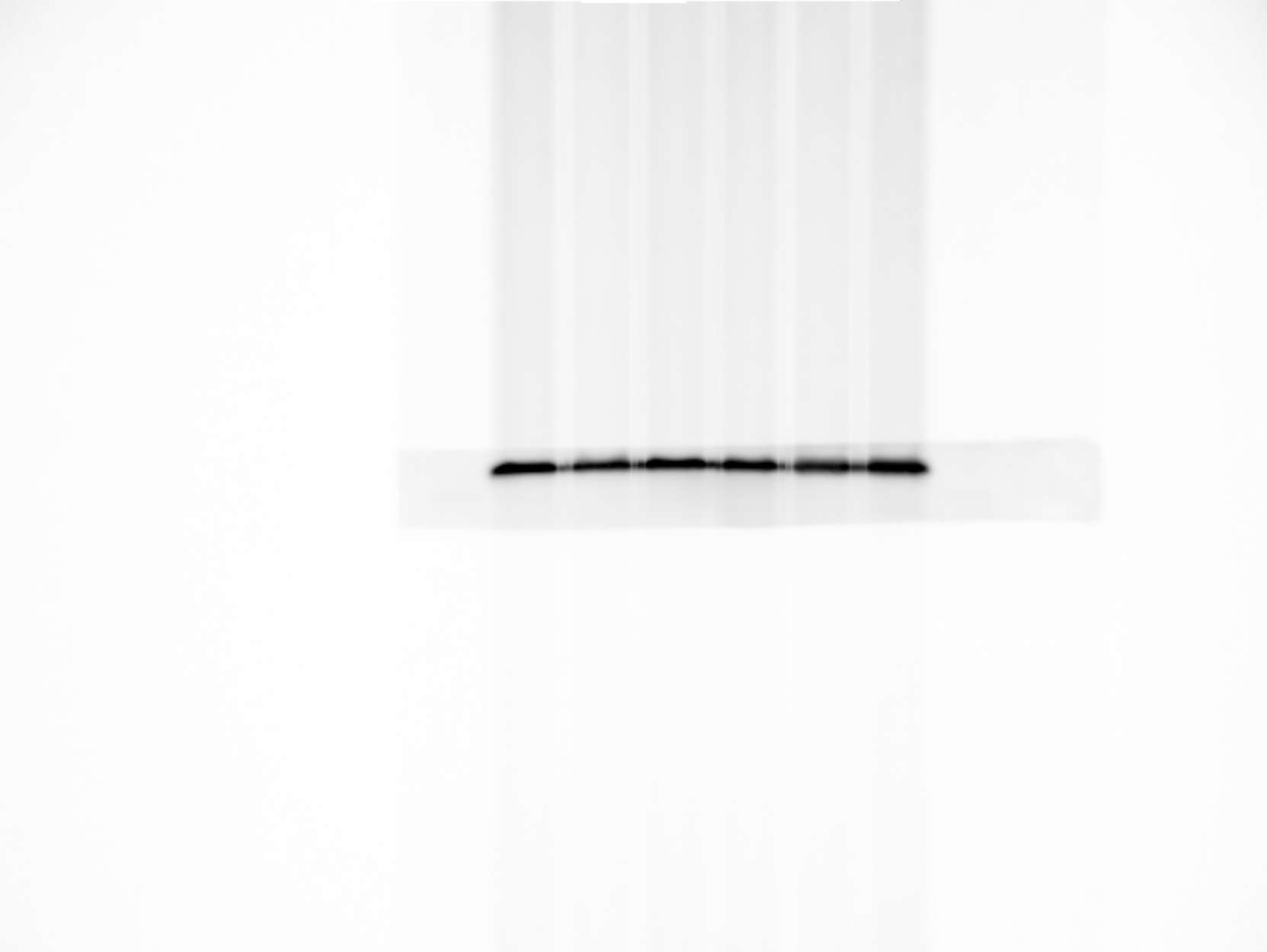

Supplement: Supplementary file 1 [file Datasheet1.zip › original raw files of Figure1/Figure 1E/membrane4-GAPDH.tif]

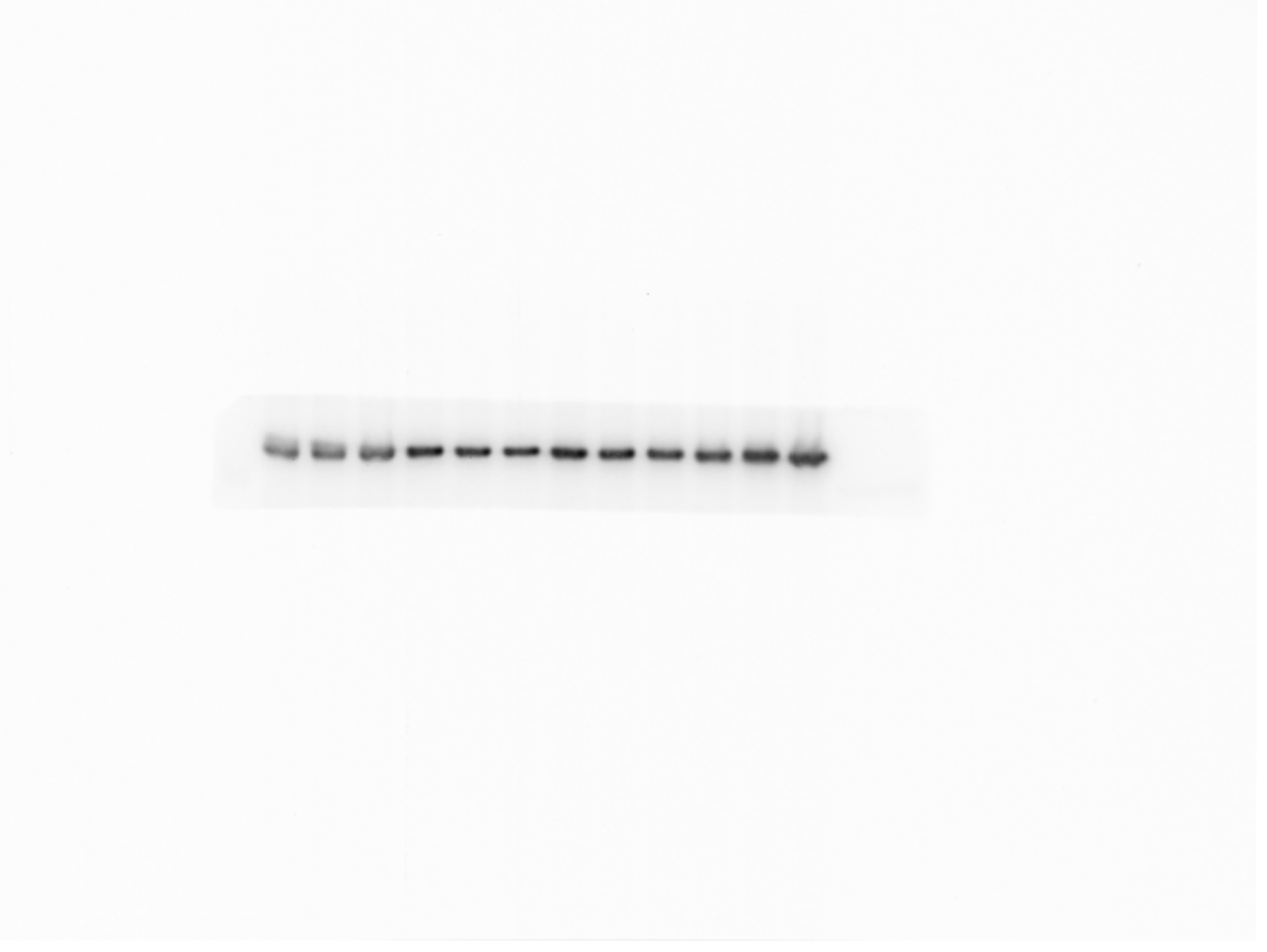

Supplement: Supplementary file 1 [file Datasheet1.zip › original raw files of Figure1/Figure 1E/membrane5-GAPDH.tif]

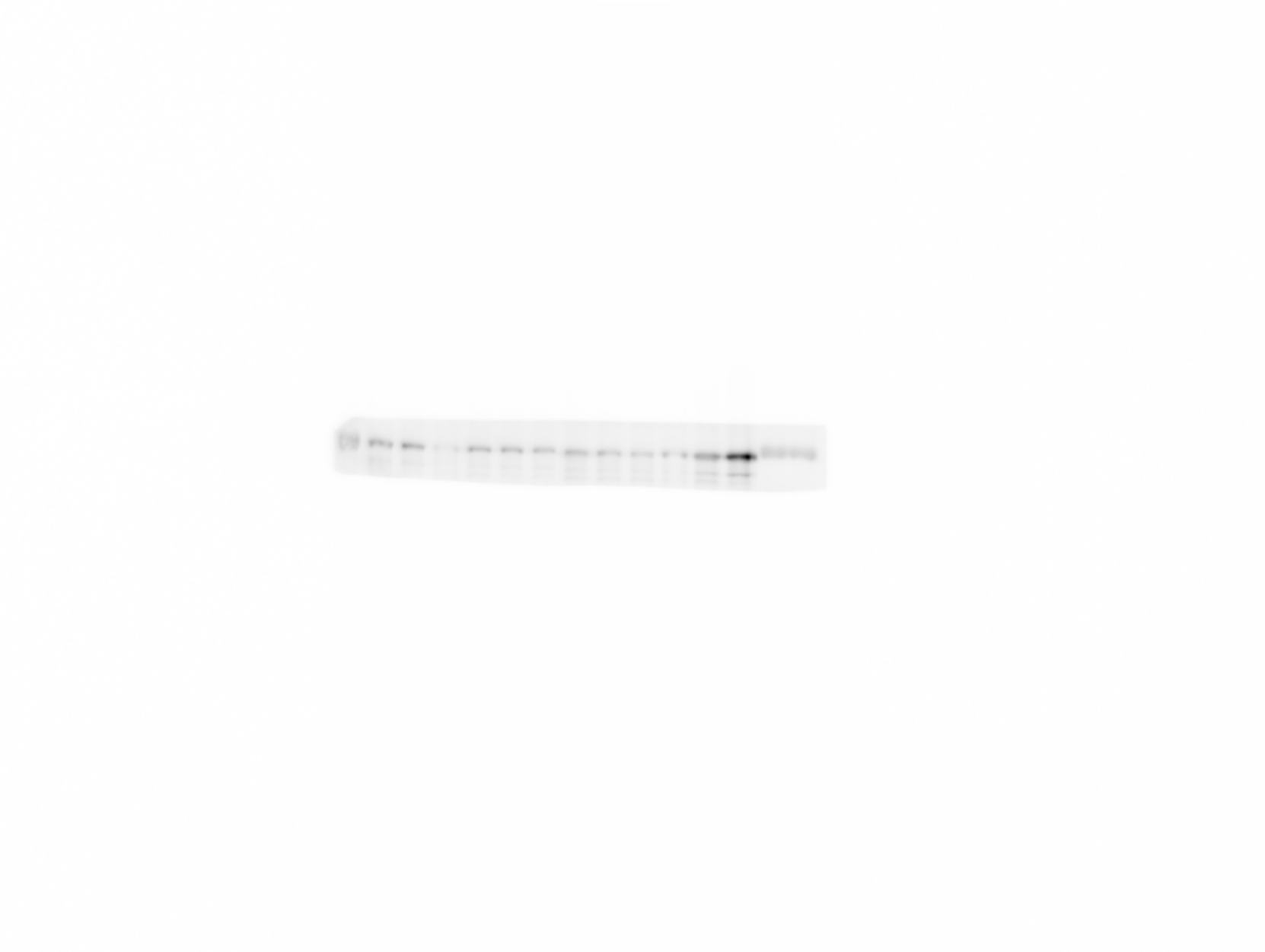

Supplement: Supplementary file 1 [file Datasheet1.zip › original raw files of Figure1/Figure 1E/membrane5-YTHDF3.tif]

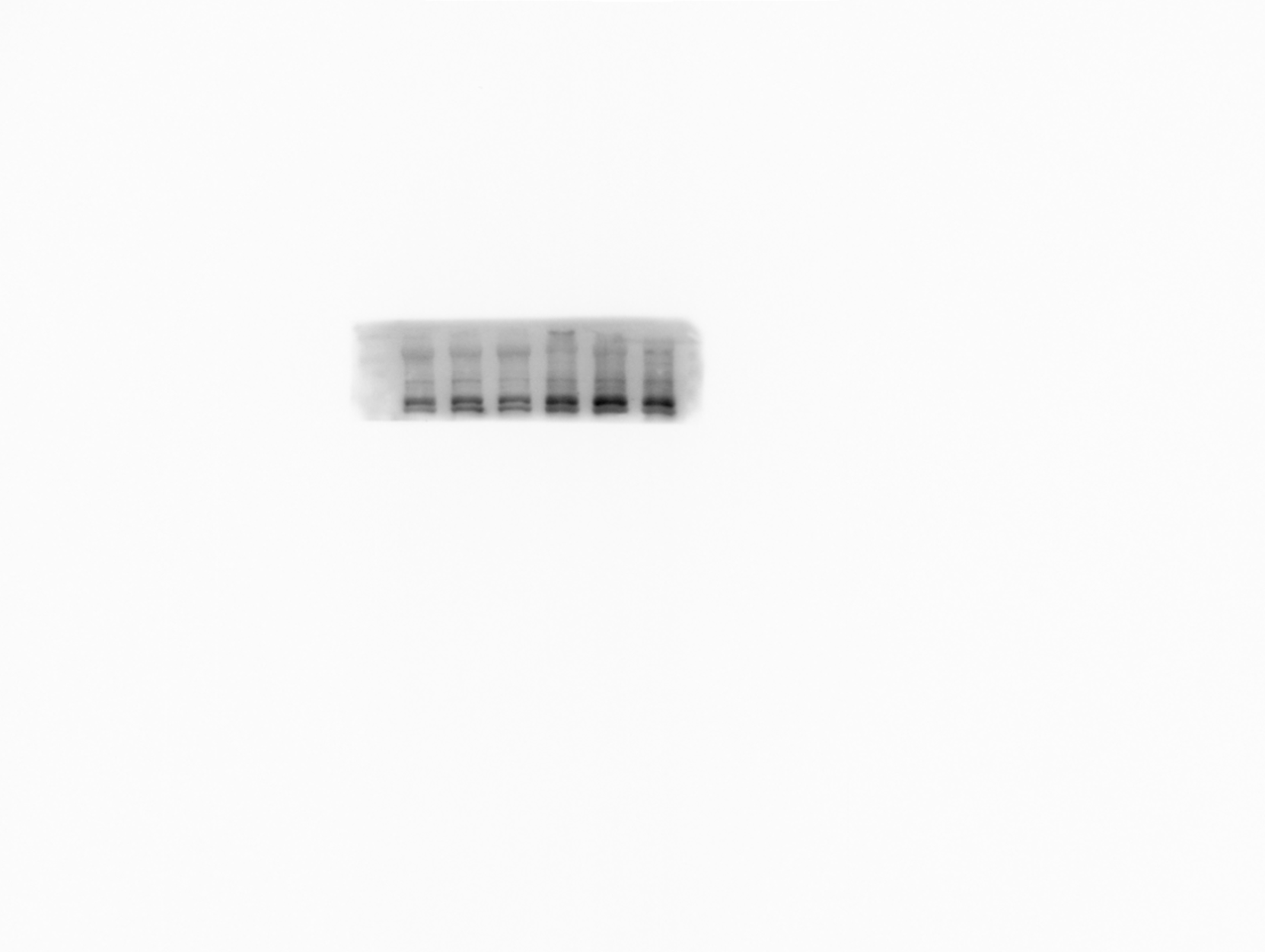

Supplement: Supplementary file 1 [file Datasheet1.zip › original raw files of Figure1/Figure 1E/membrane1-YTHDC1.tif]

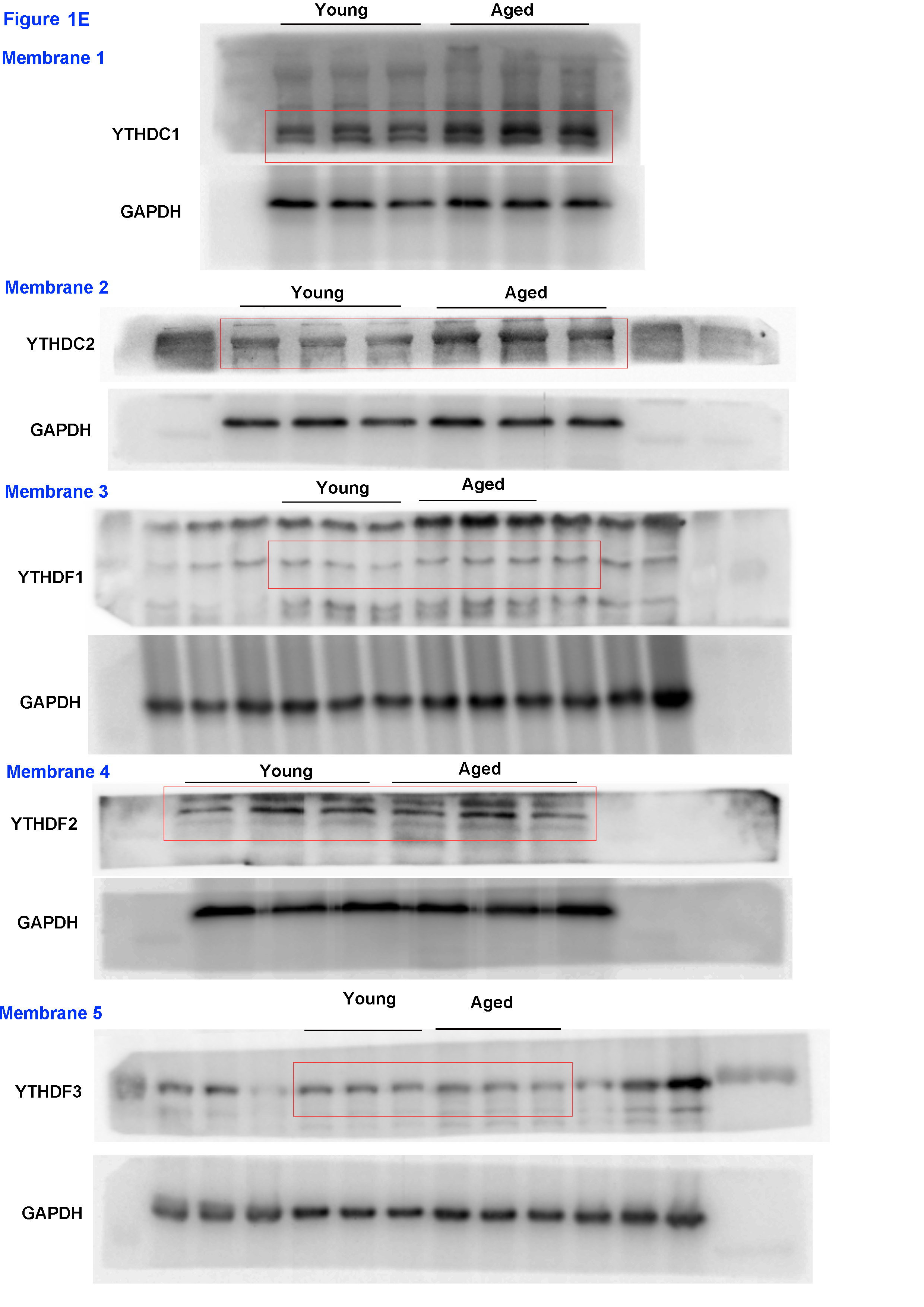

Supplement: Supplementary file 1 [file Datasheet1.zip › original raw files of Figure1/Figure 1E/Integration and annotation of the original images.tif]

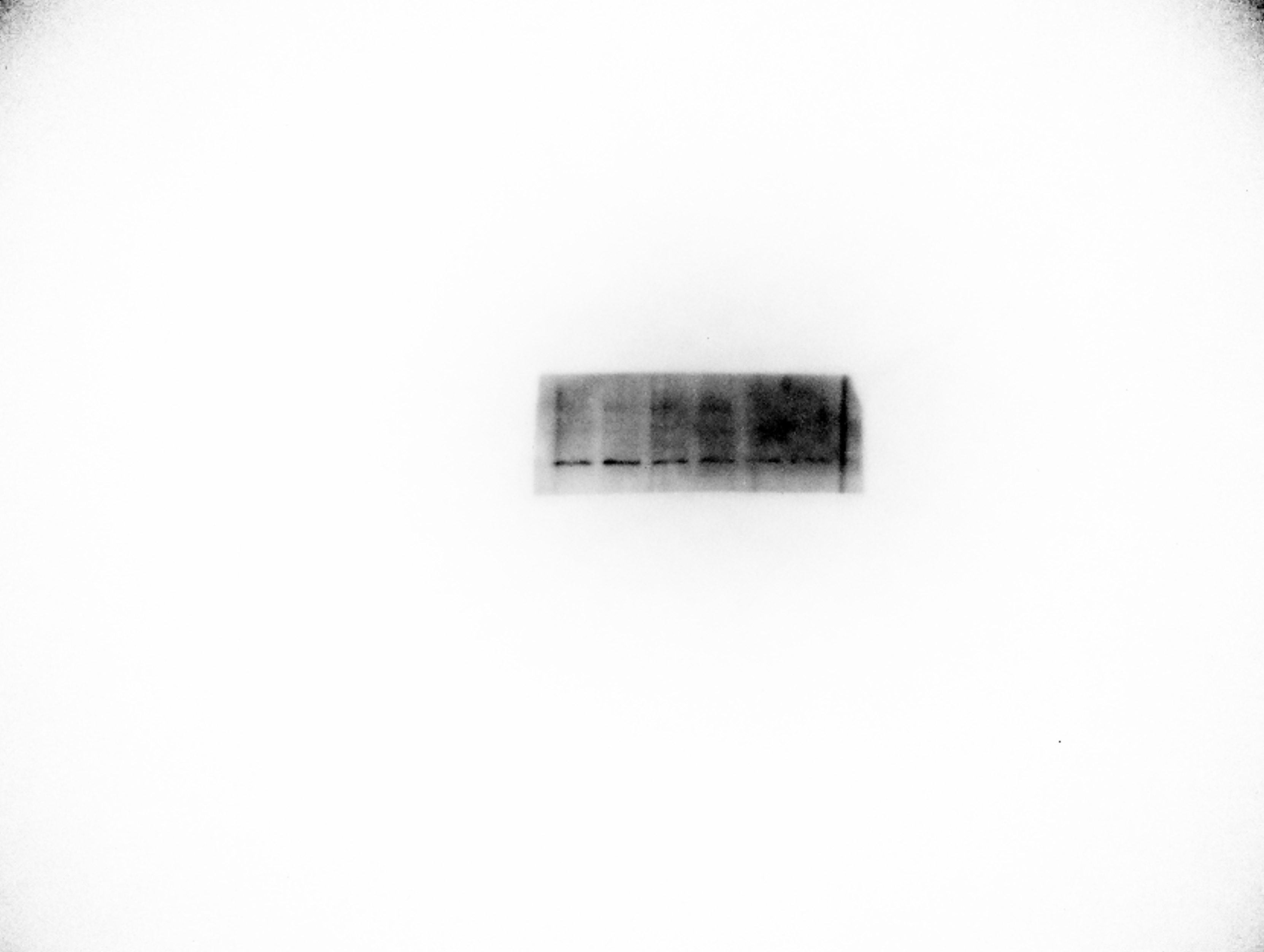

Supplement: Supplementary file 1 [file Datasheet1.zip › original raw files of Figure1/Figure 1G/membrane2-METTL14.tif]

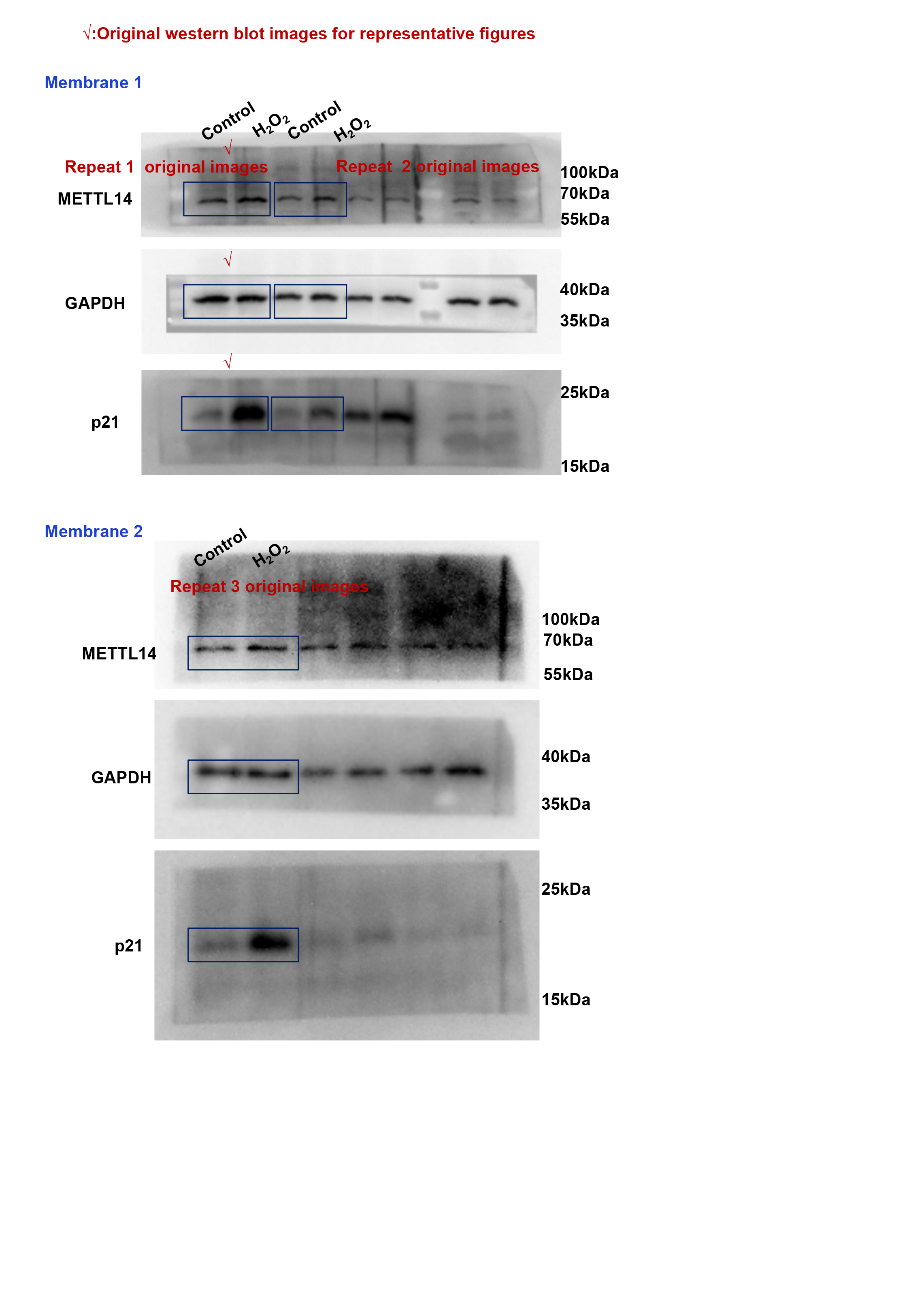

Supplement: Supplementary file 1 [file Datasheet1.zip › original raw files of Figure1/Figure 1G/Integration and annotation of the original images.tif]

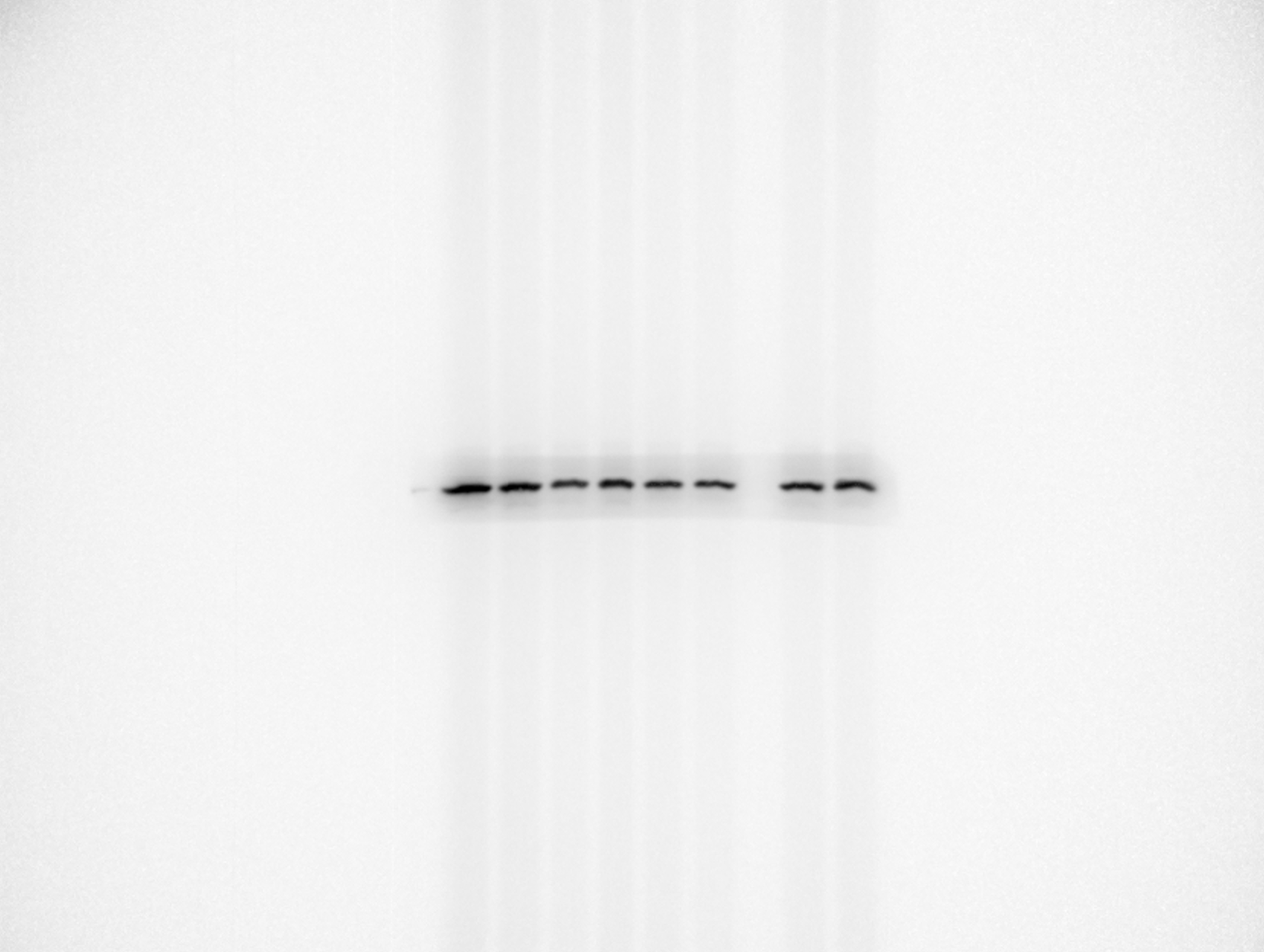

Supplement: Supplementary file 1 [file Datasheet1.zip › original raw files of Figure1/Figure 1G/membrane1-GAPDH.tif]

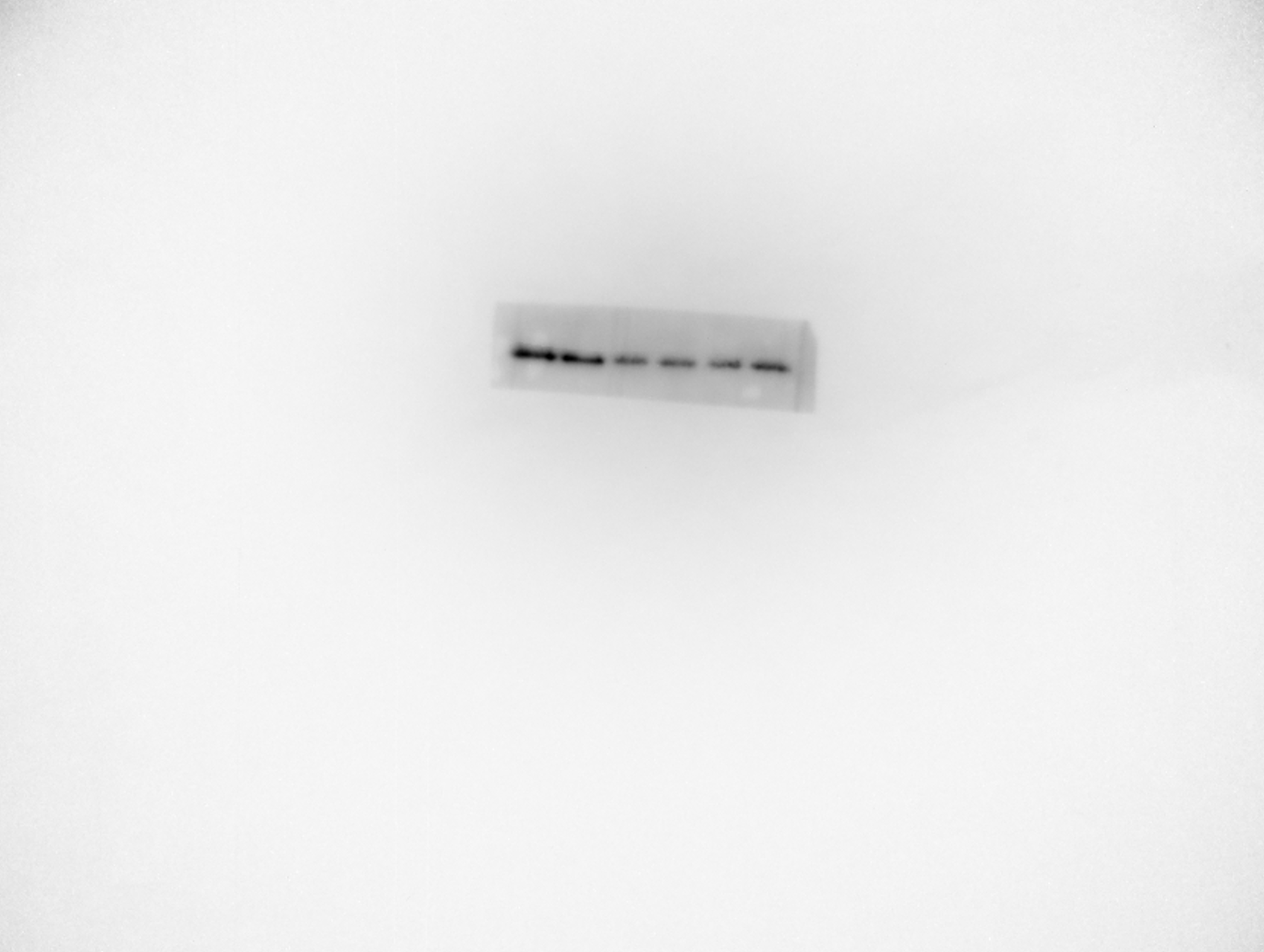

Supplement: Supplementary file 1 [file Datasheet1.zip › original raw files of Figure1/Figure 1G/membrane2-GAPDH.tif]

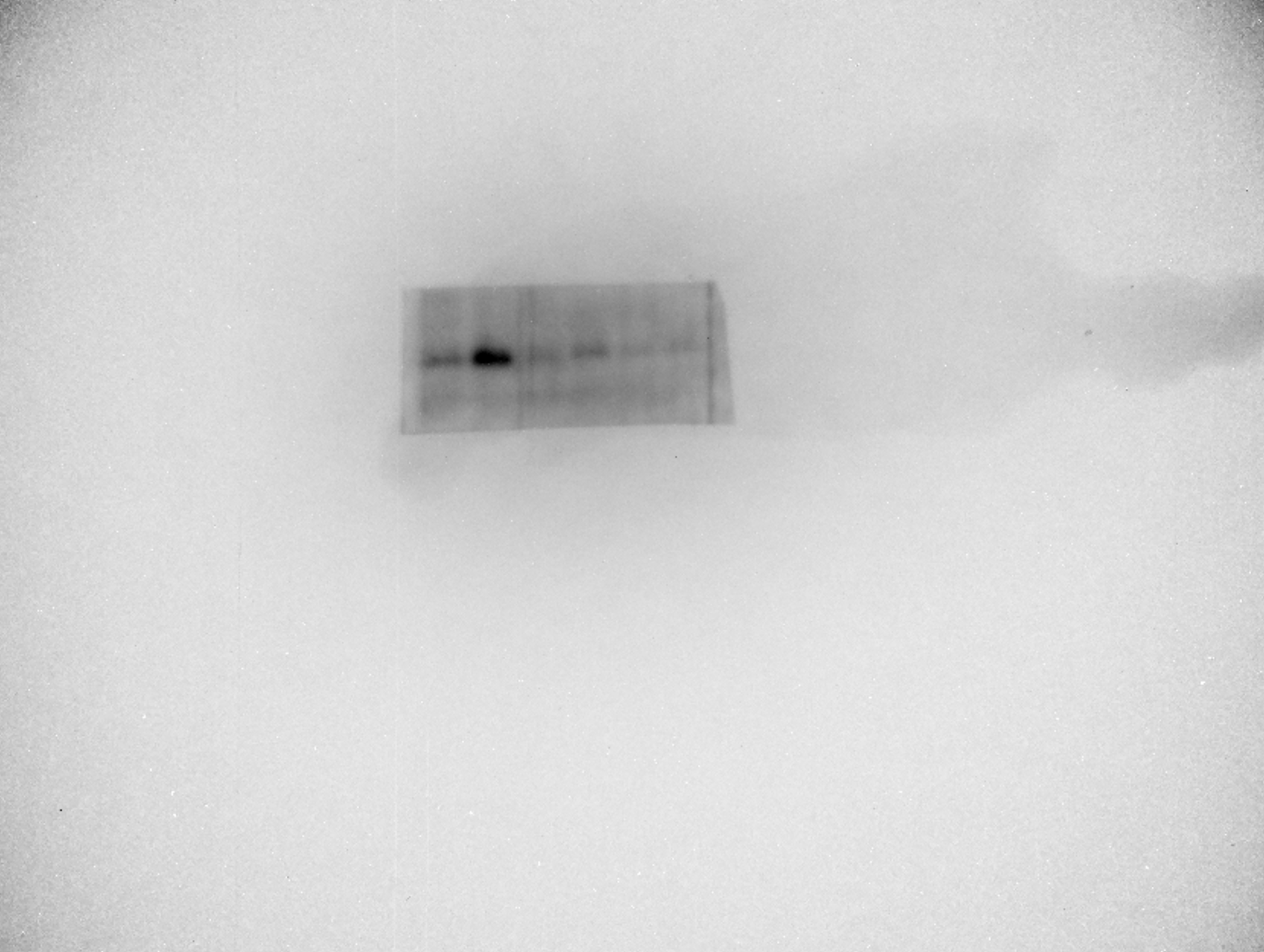

Supplement: Supplementary file 1 [file Datasheet1.zip › original raw files of Figure1/Figure 1G/membrane2-p21.tif]

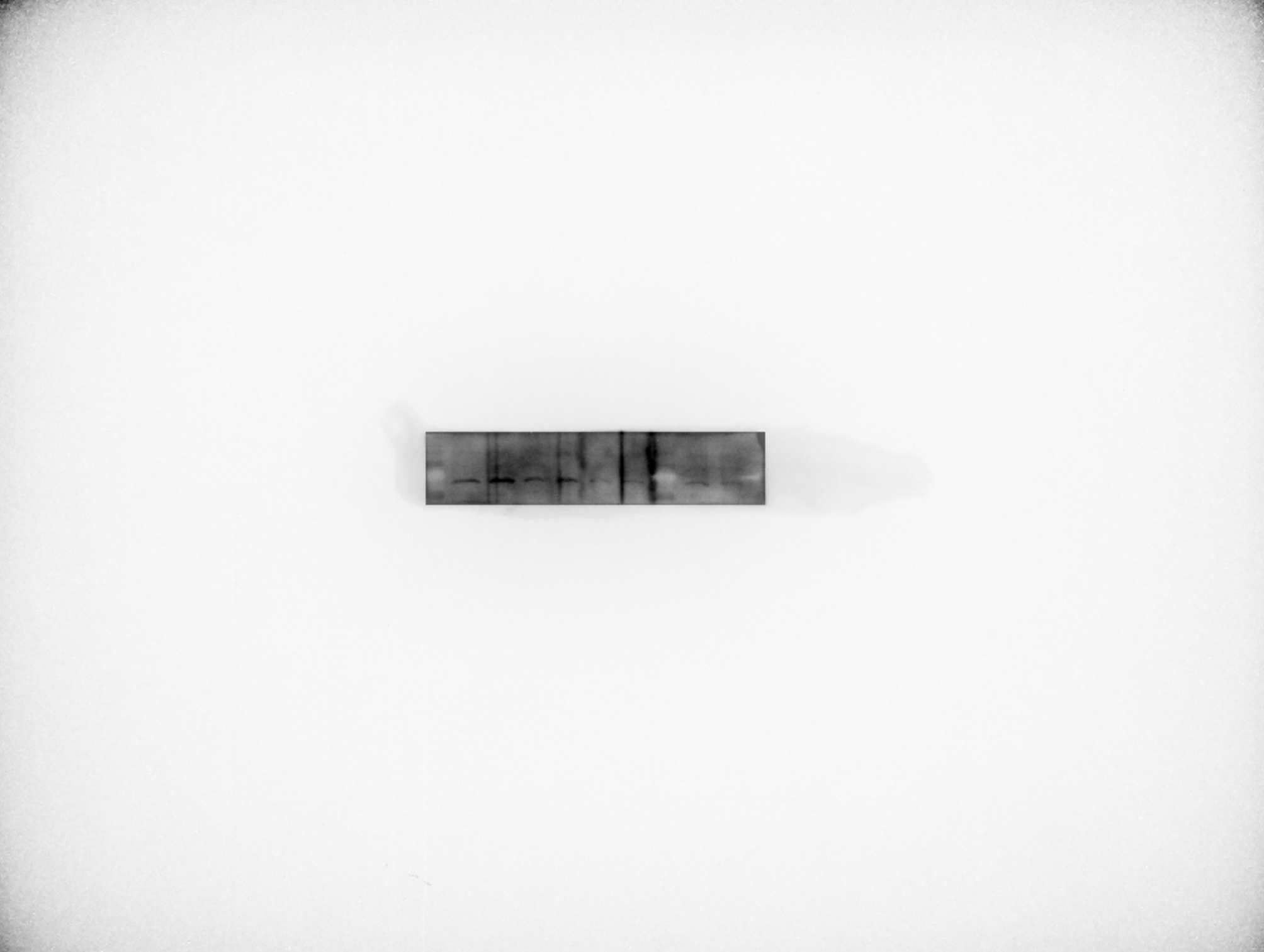

Supplement: Supplementary file 1 [file Datasheet1.zip › original raw files of Figure1/Figure 1G/membrane1-METTL14.tif]

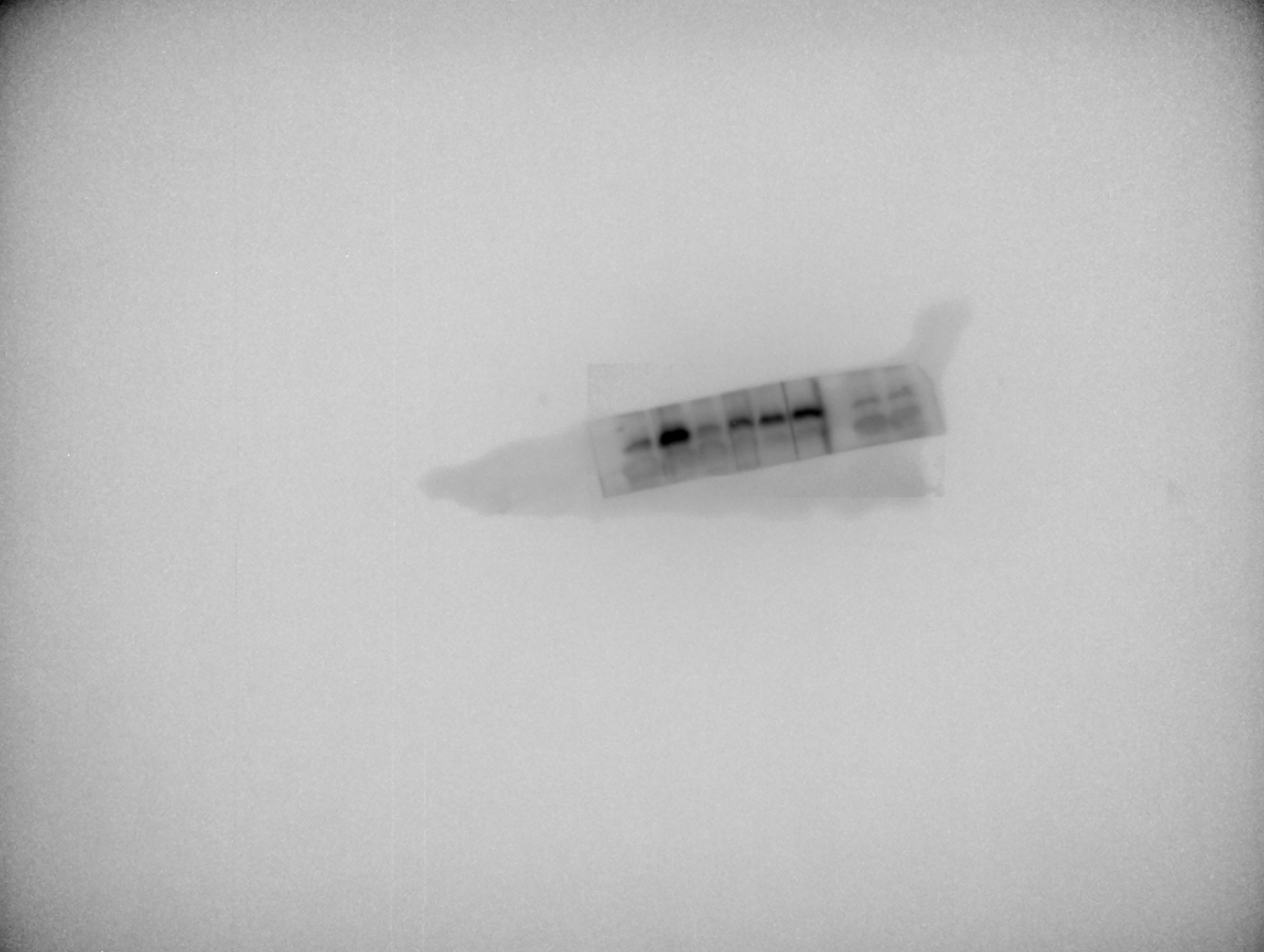

Supplement: Supplementary file 1 [file Datasheet1.zip › original raw files of Figure1/Figure 1G/membrane1-p21.tif]

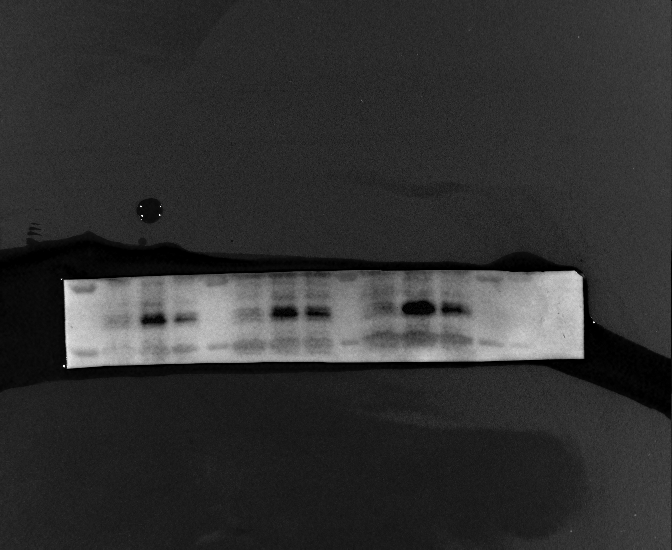

Supplement: Supplementary file 1 [file Datasheet1.zip › original raw files of Figure1/Figure 1I/membrane3-p21.tif]

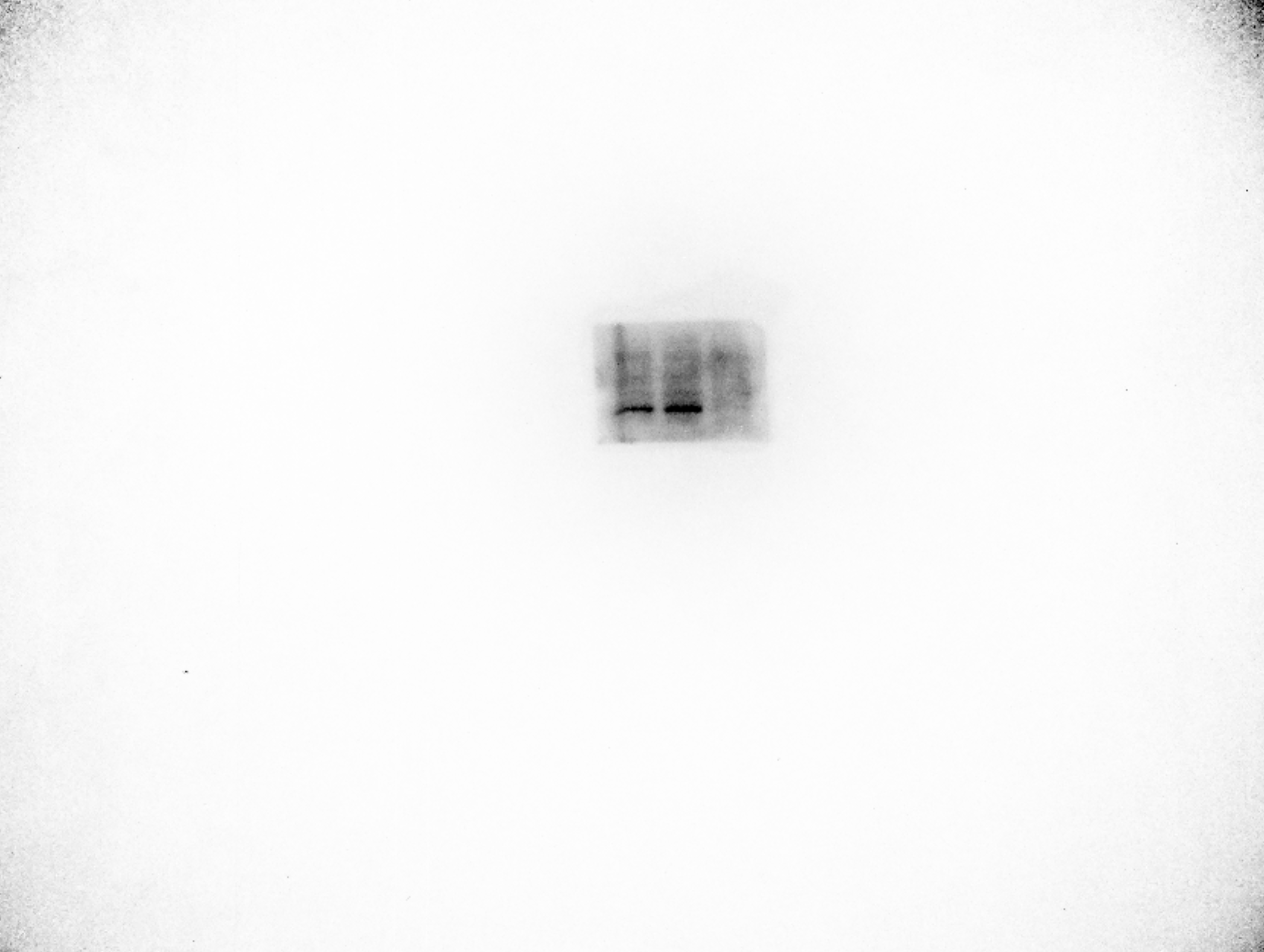

Supplement: Supplementary file 1 [file Datasheet1.zip › original raw files of Figure1/Figure 1I/membrane1-METTL14.tif]

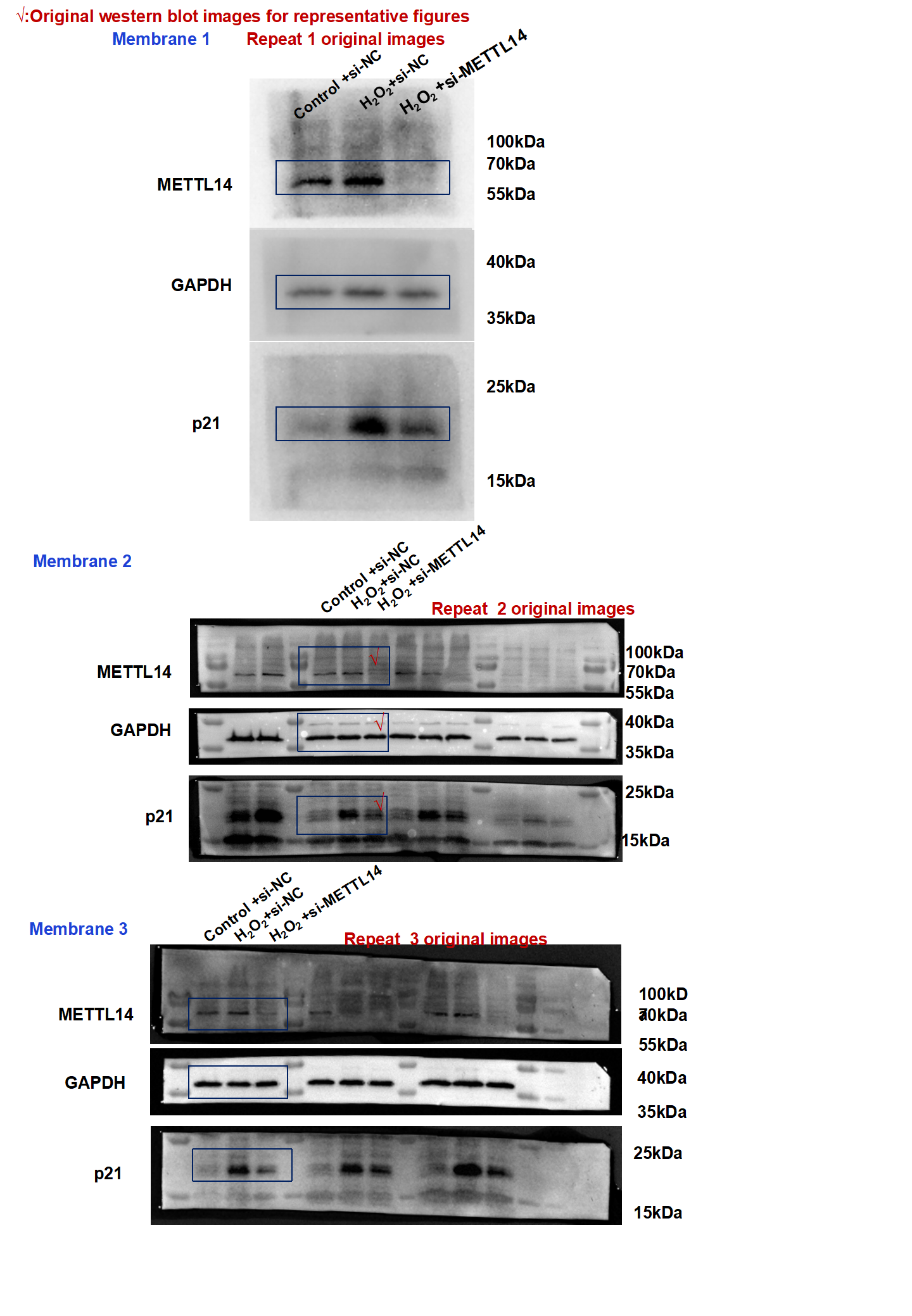

Supplement: Supplementary file 1 [file Datasheet1.zip › original raw files of Figure1/Figure 1I/Integration and annotation of the original images.tif]

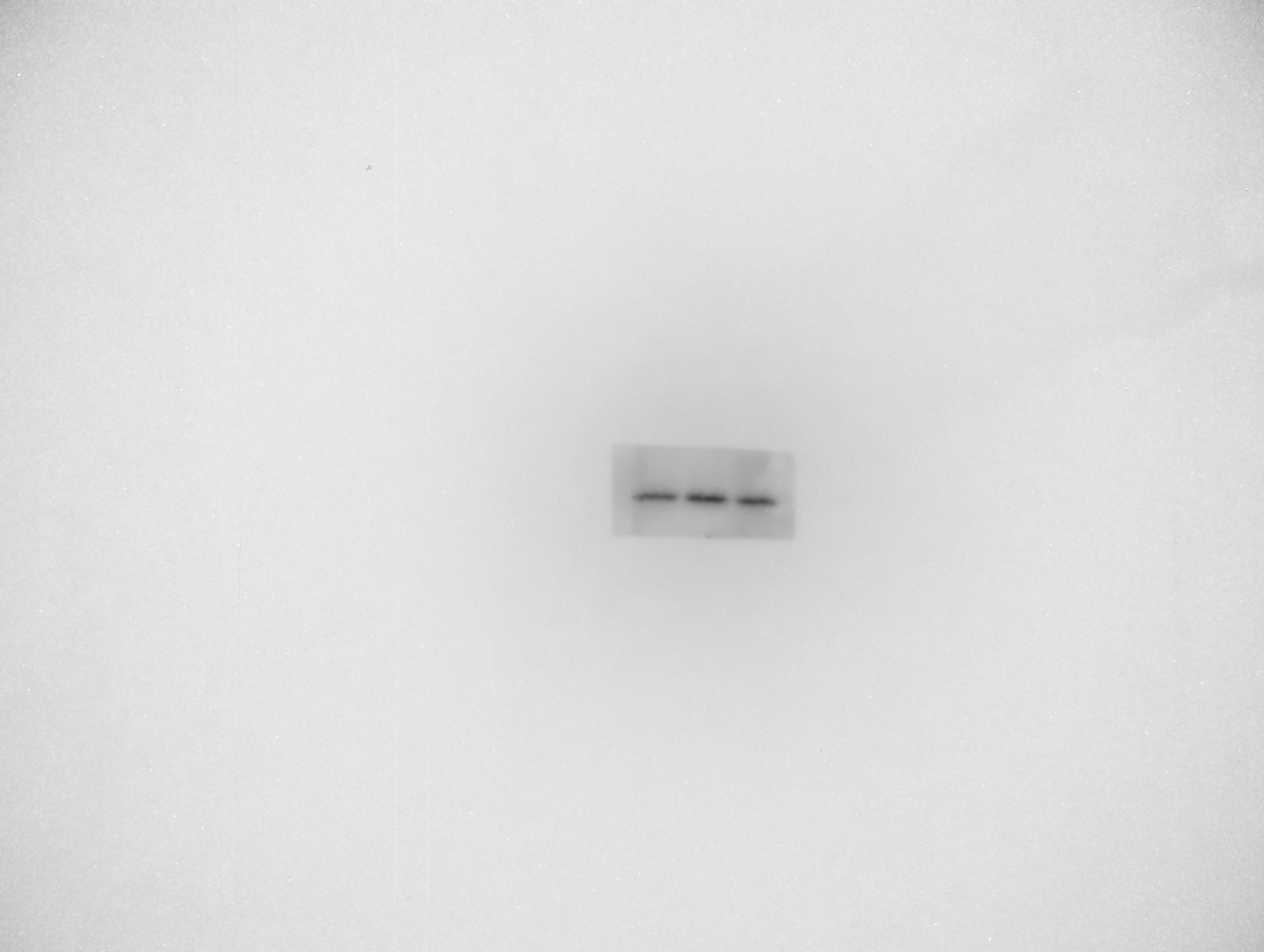

Supplement: Supplementary file 1 [file Datasheet1.zip › original raw files of Figure1/Figure 1I/membrane1-GAPDH.tif]

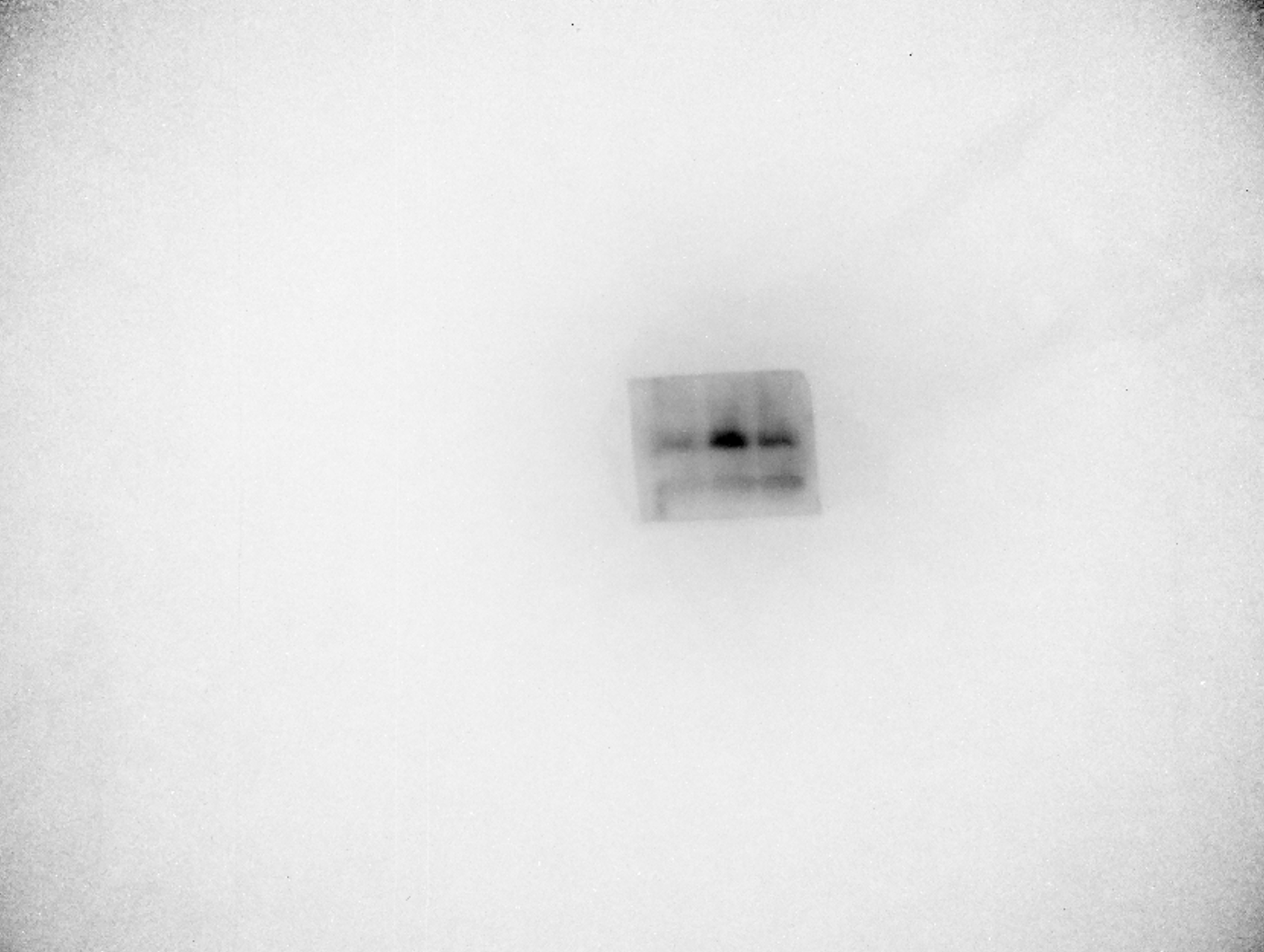

Supplement: Supplementary file 1 [file Datasheet1.zip › original raw files of Figure1/Figure 1I/membrane1-p21.tif]

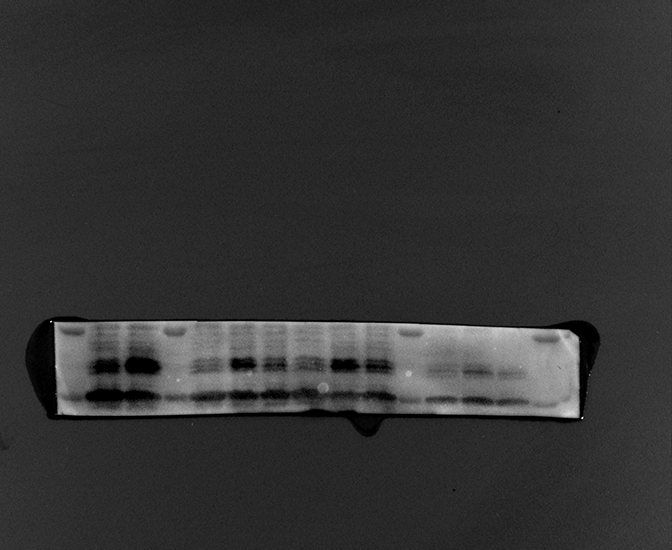

Supplement: Supplementary file 1 [file Datasheet1.zip › original raw files of Figure1/Figure 1I/membrane2-p21.tif]

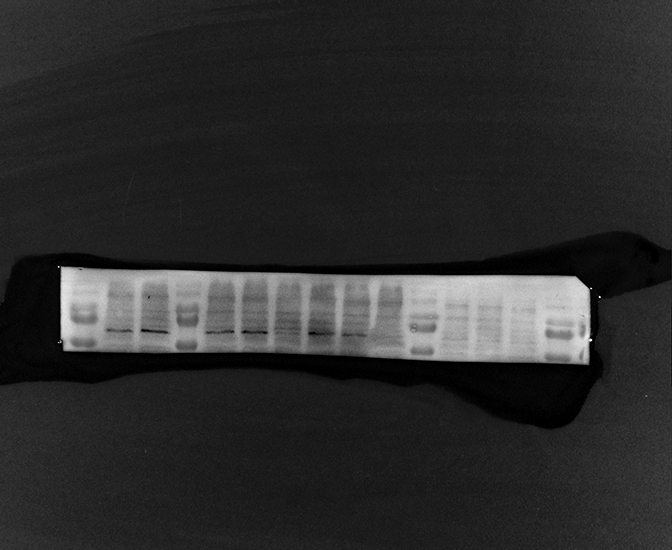

Supplement: Supplementary file 1 [file Datasheet1.zip › original raw files of Figure1/Figure 1I/membrane2-METTL14.tif]

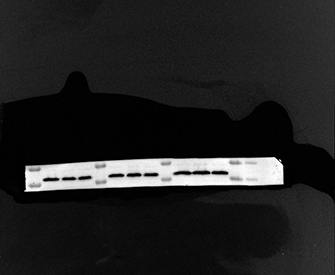

Supplement: Supplementary file 1 [file Datasheet1.zip › original raw files of Figure1/Figure 1I/membrane3-GAPDH.tif]

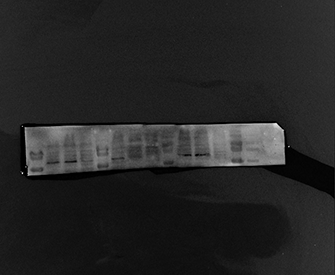

Supplement: Supplementary file 1 [file Datasheet1.zip › original raw files of Figure1/Figure 1I/membrane3-METTL14.tif]

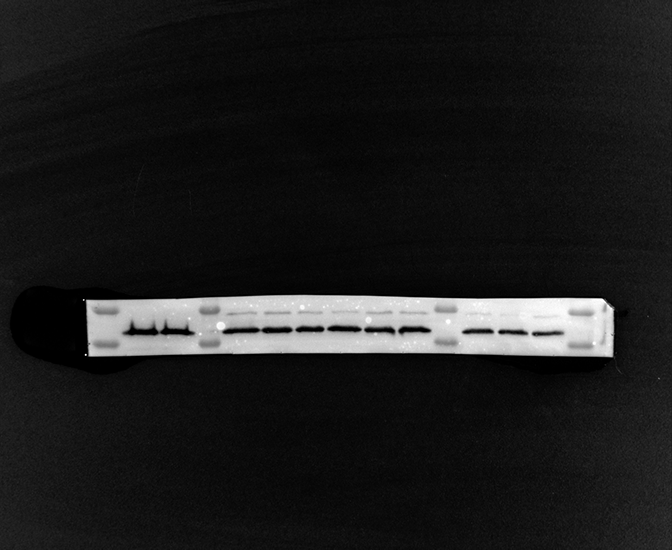

Supplement: Supplementary file 1 [file Datasheet1.zip › original raw files of Figure1/Figure 1I/membrane2-GAPDH.tif]

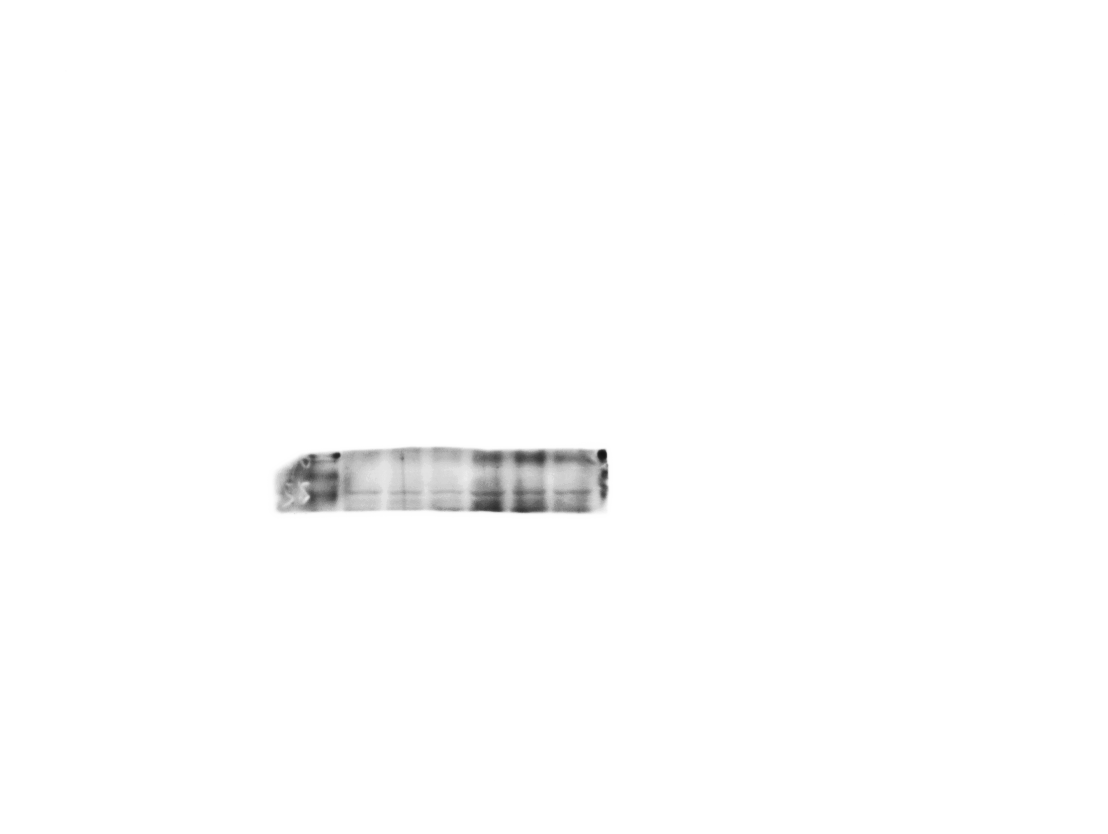

Supplement: Supplementary file 2 [file Datasheet2.zip › original raw files of Supplementary Figure 1B/membrane3-P53.tif]

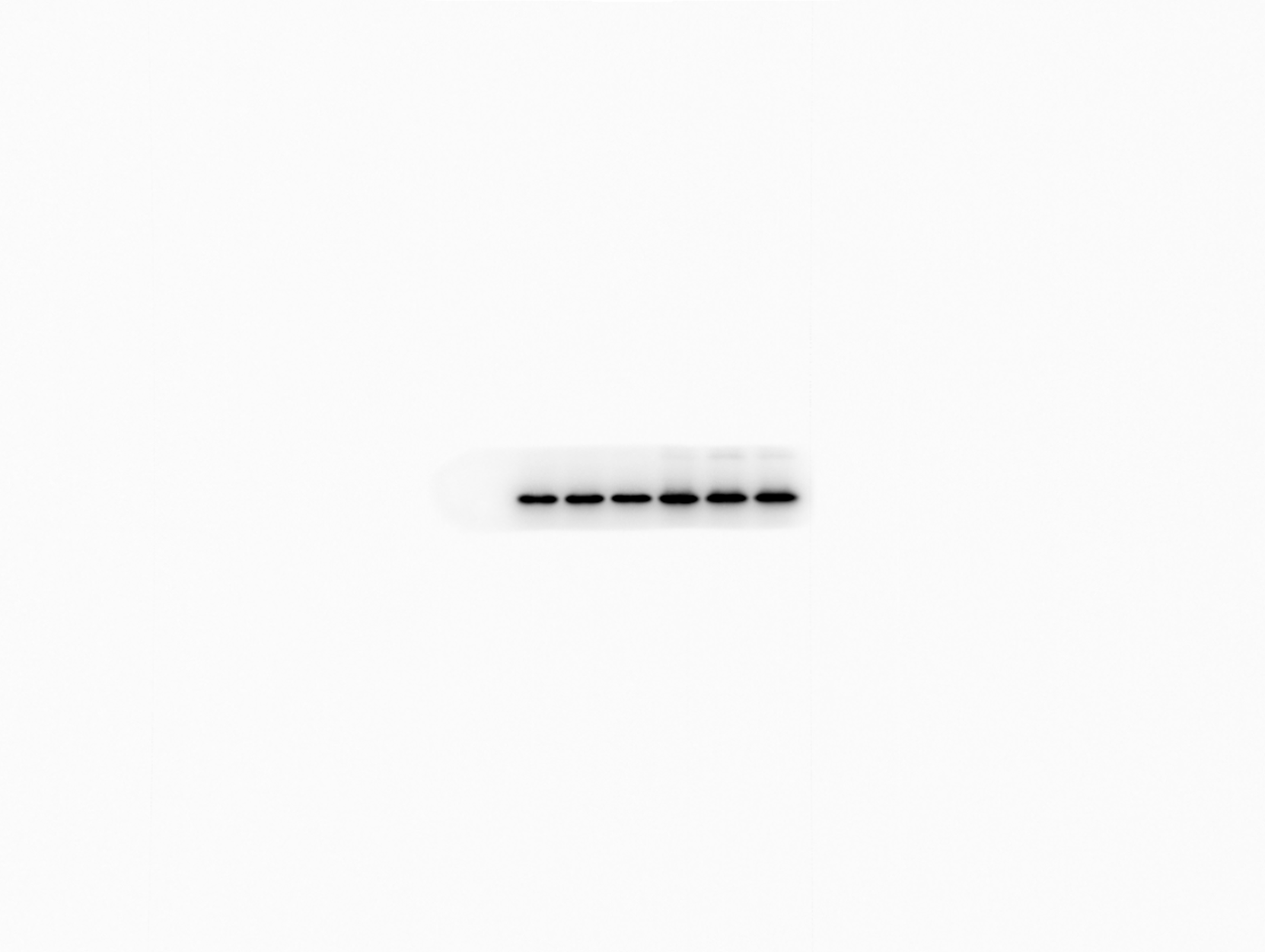

Supplement: Supplementary file 2 [file Datasheet2.zip › original raw files of Supplementary Figure 1B/membrane1-GAPDH.tif]

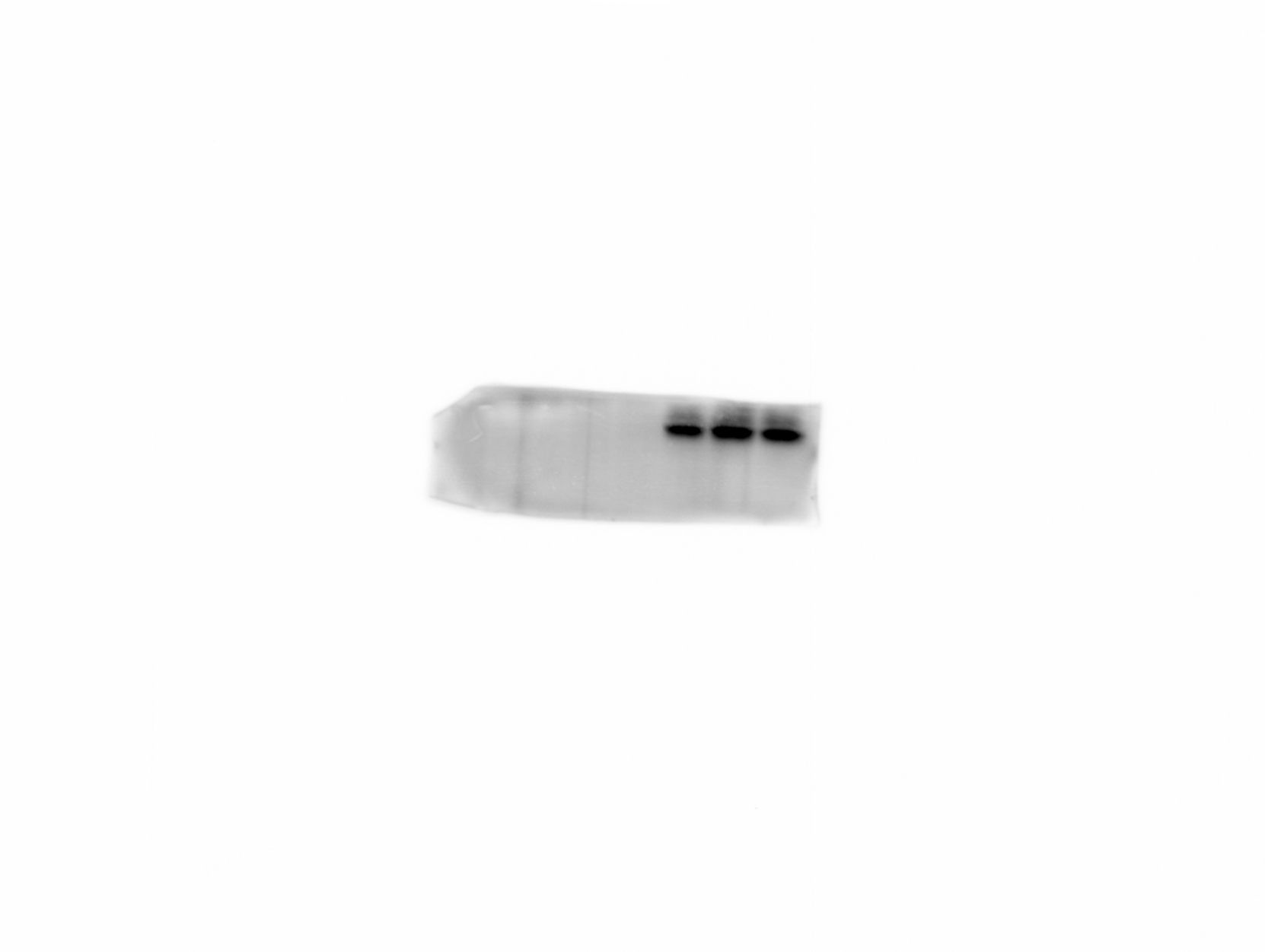

Supplement: Supplementary file 2 [file Datasheet2.zip › original raw files of Supplementary Figure 1B/membrane1-γH2Ax.tif]

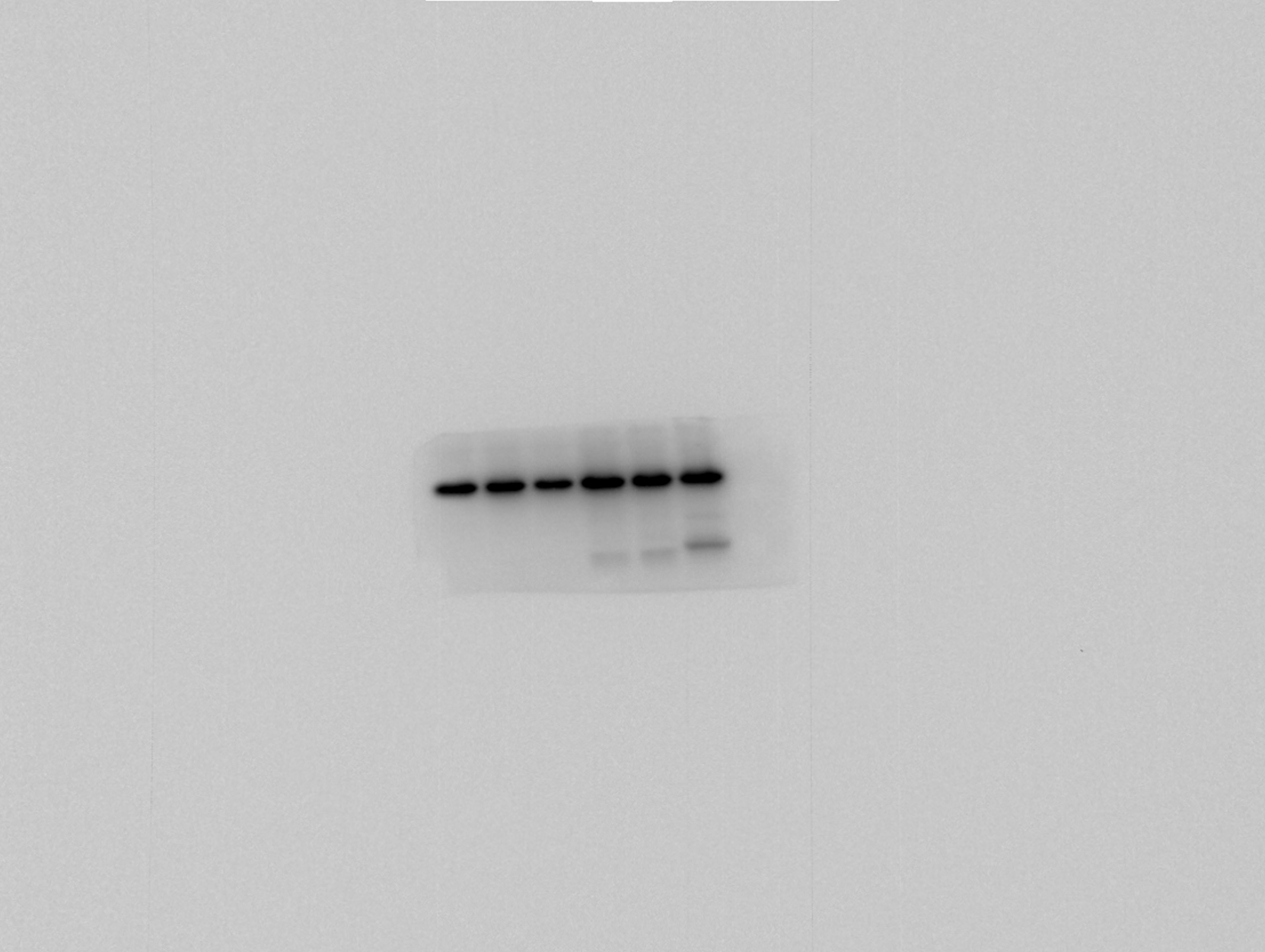

Supplement: Supplementary file 2 [file Datasheet2.zip › original raw files of Supplementary Figure 1B/membrane2-GAPDH.tif]

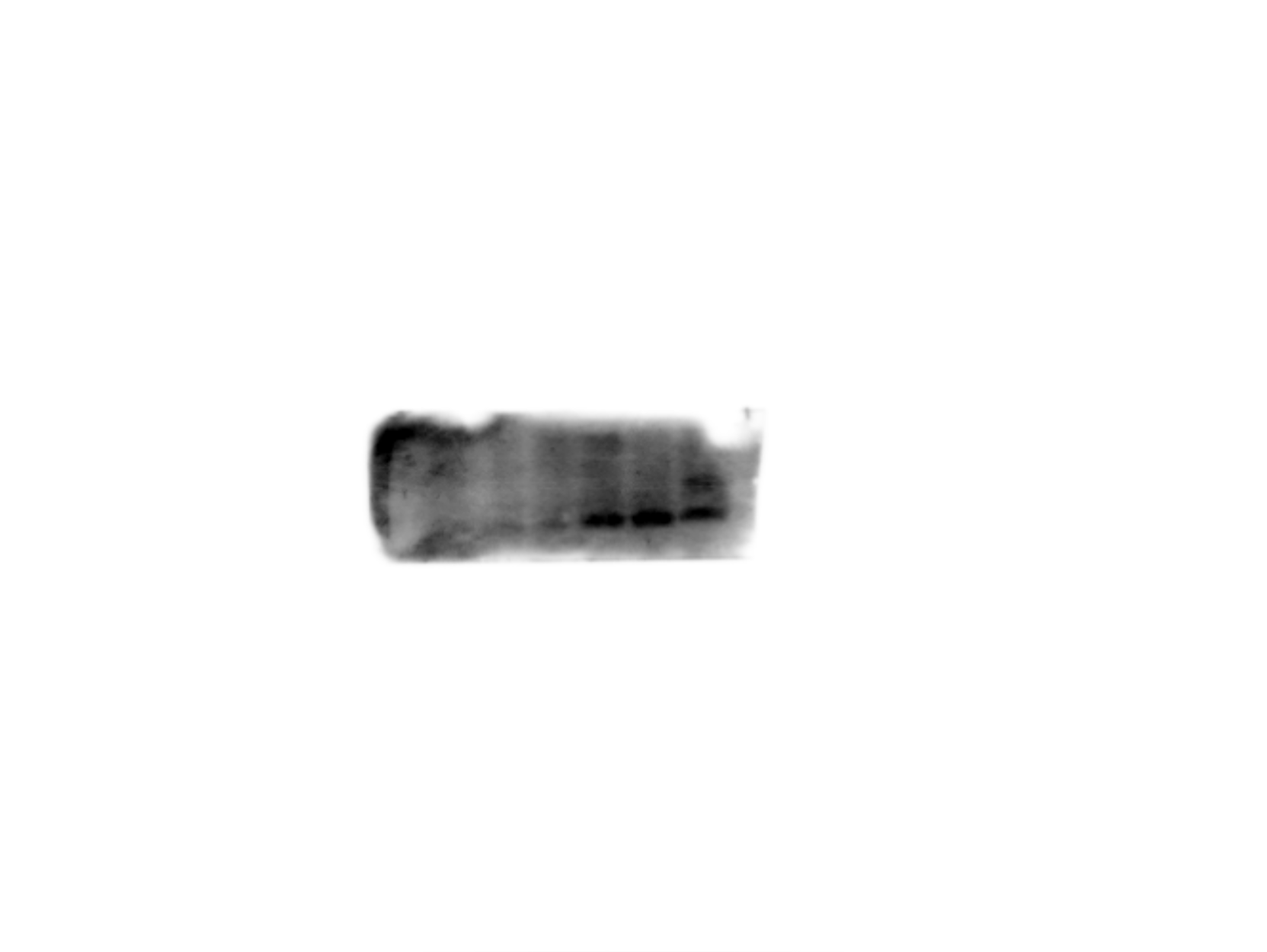

Supplement: Supplementary file 2 [file Datasheet2.zip › original raw files of Supplementary Figure 1B/membrane2-P16.tif]

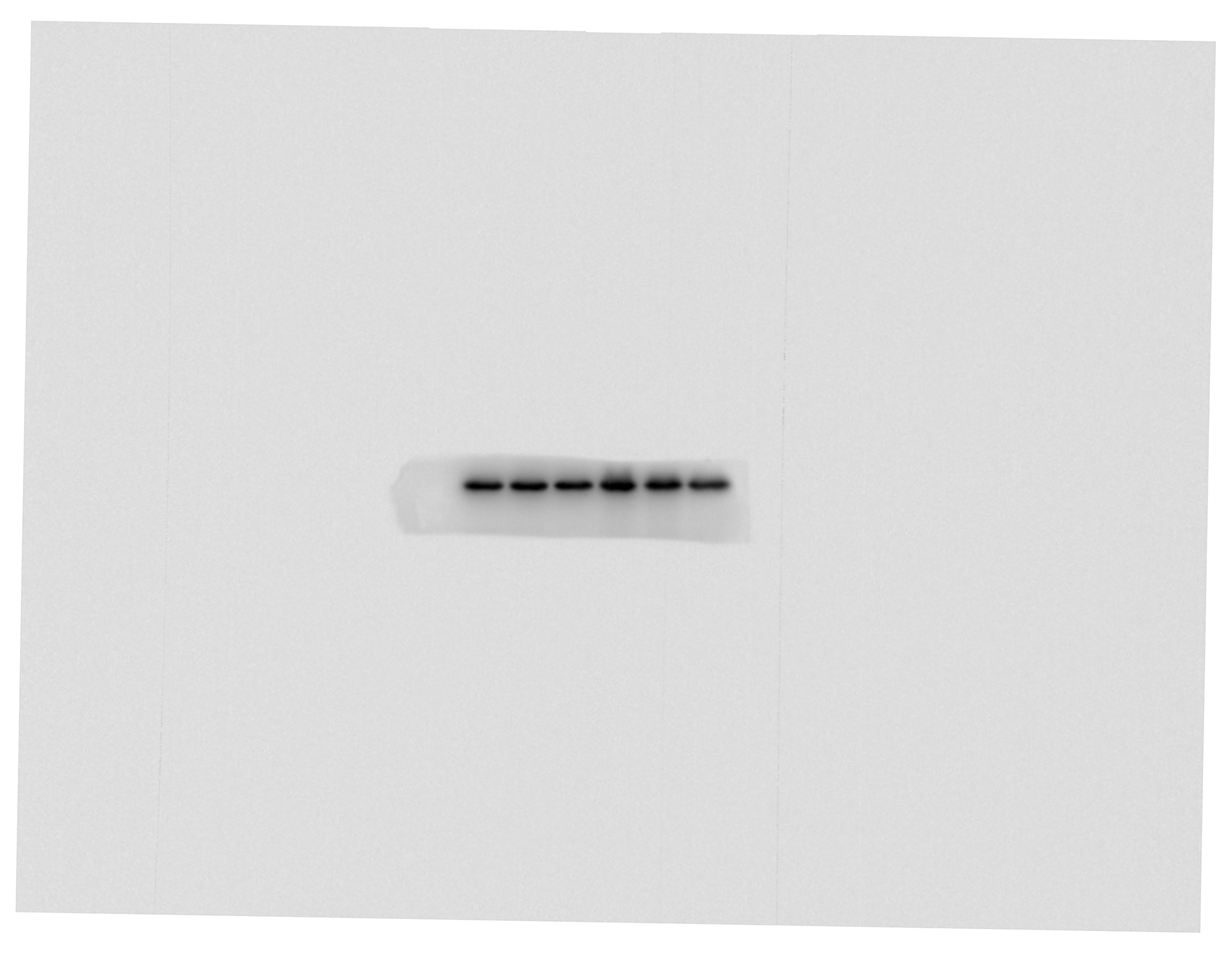

Supplement: Supplementary file 2 [file Datasheet2.zip › original raw files of Supplementary Figure 1B/membrane3-GAPDH.tif]

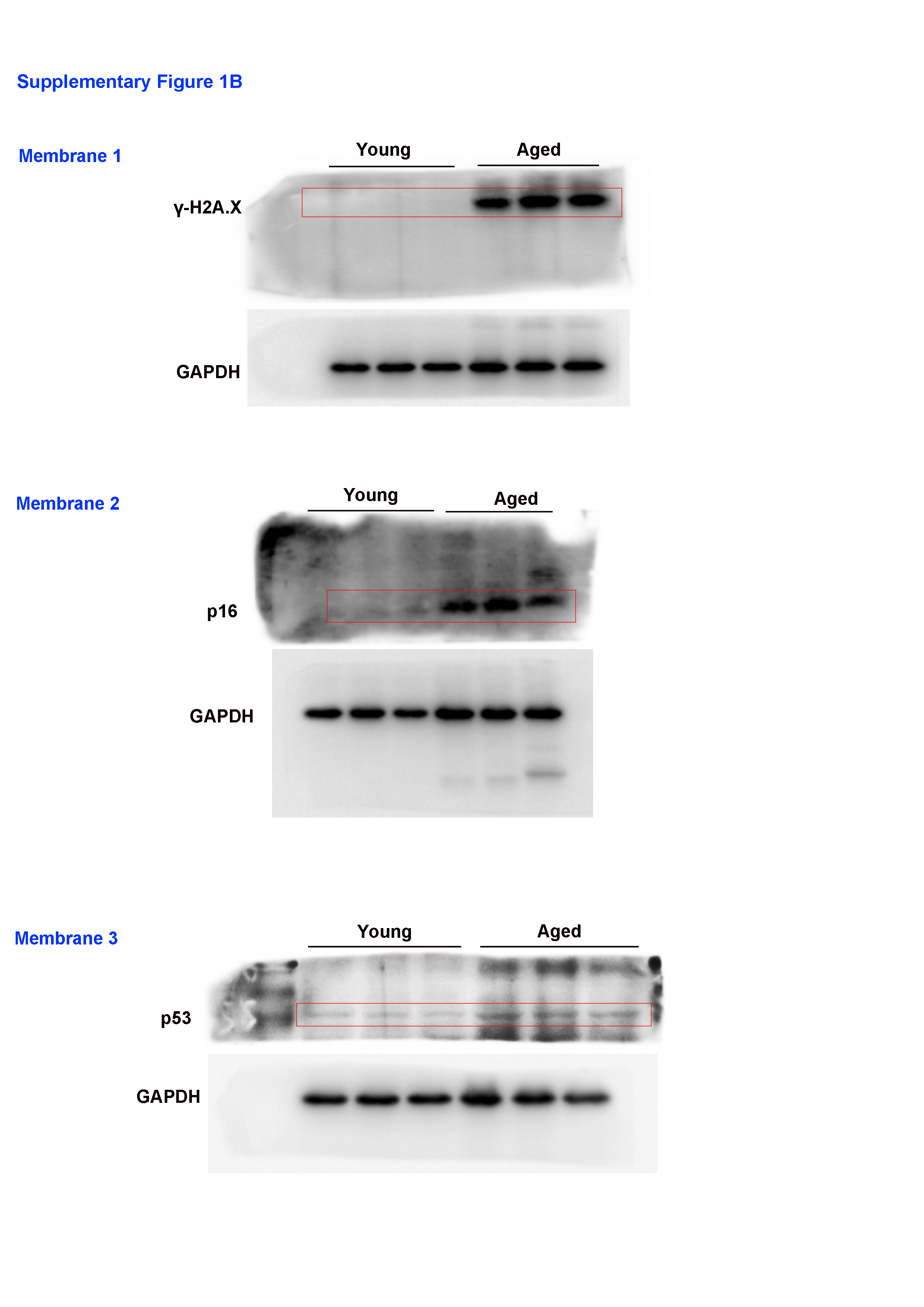

Supplement: Supplementary file 2 [file Datasheet2.zip › original raw files of Supplementary Figure 1B/Integration and annotation of the original images.tif]

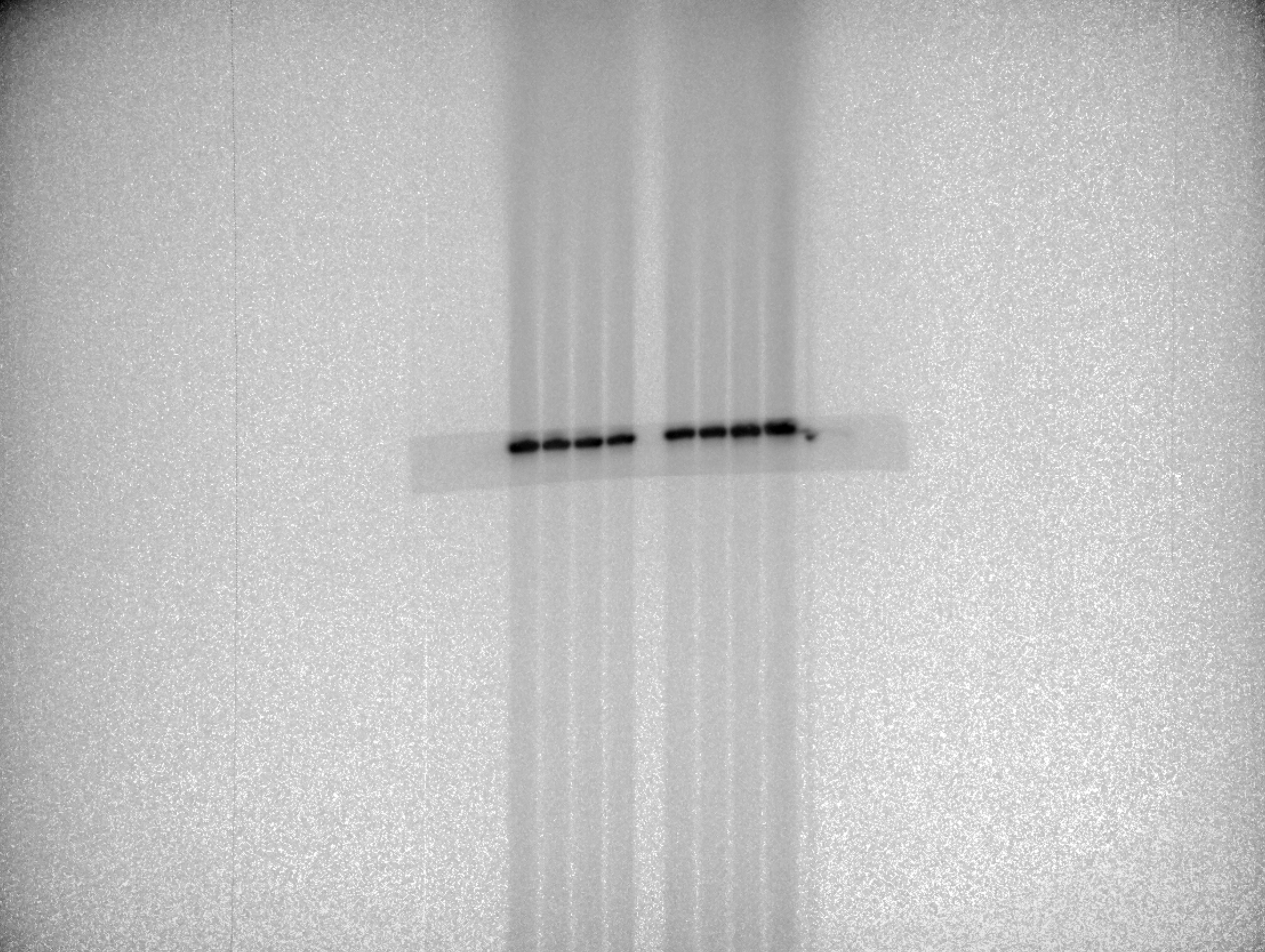

Supplement: Supplementary file 3 [file Datasheet3.zip › original raw files of Supplementary Figure 2A/membrane1-GAPDH.tif]

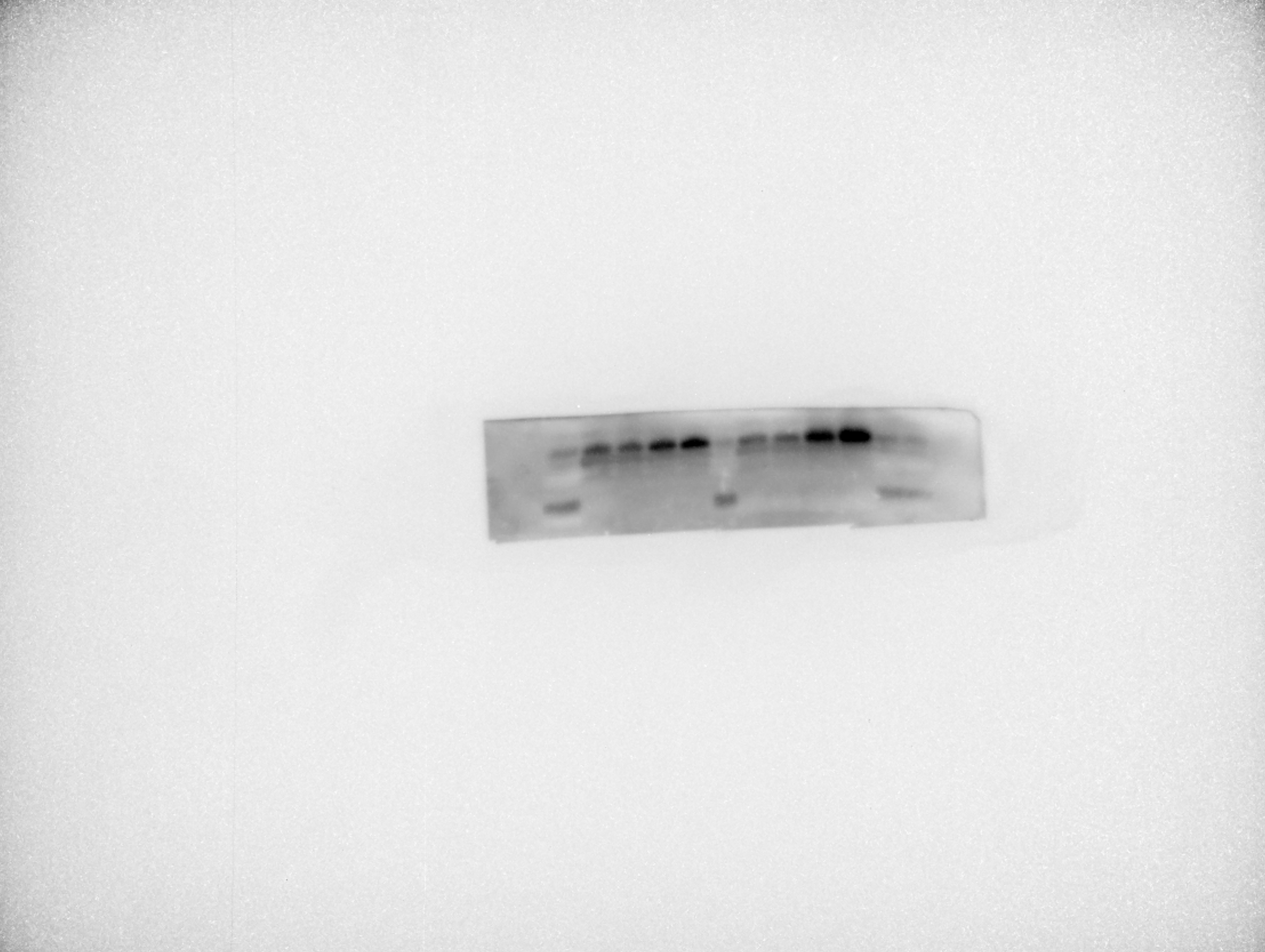

Supplement: Supplementary file 3 [file Datasheet3.zip › original raw files of Supplementary Figure 2A/membrane1-p21.tif]

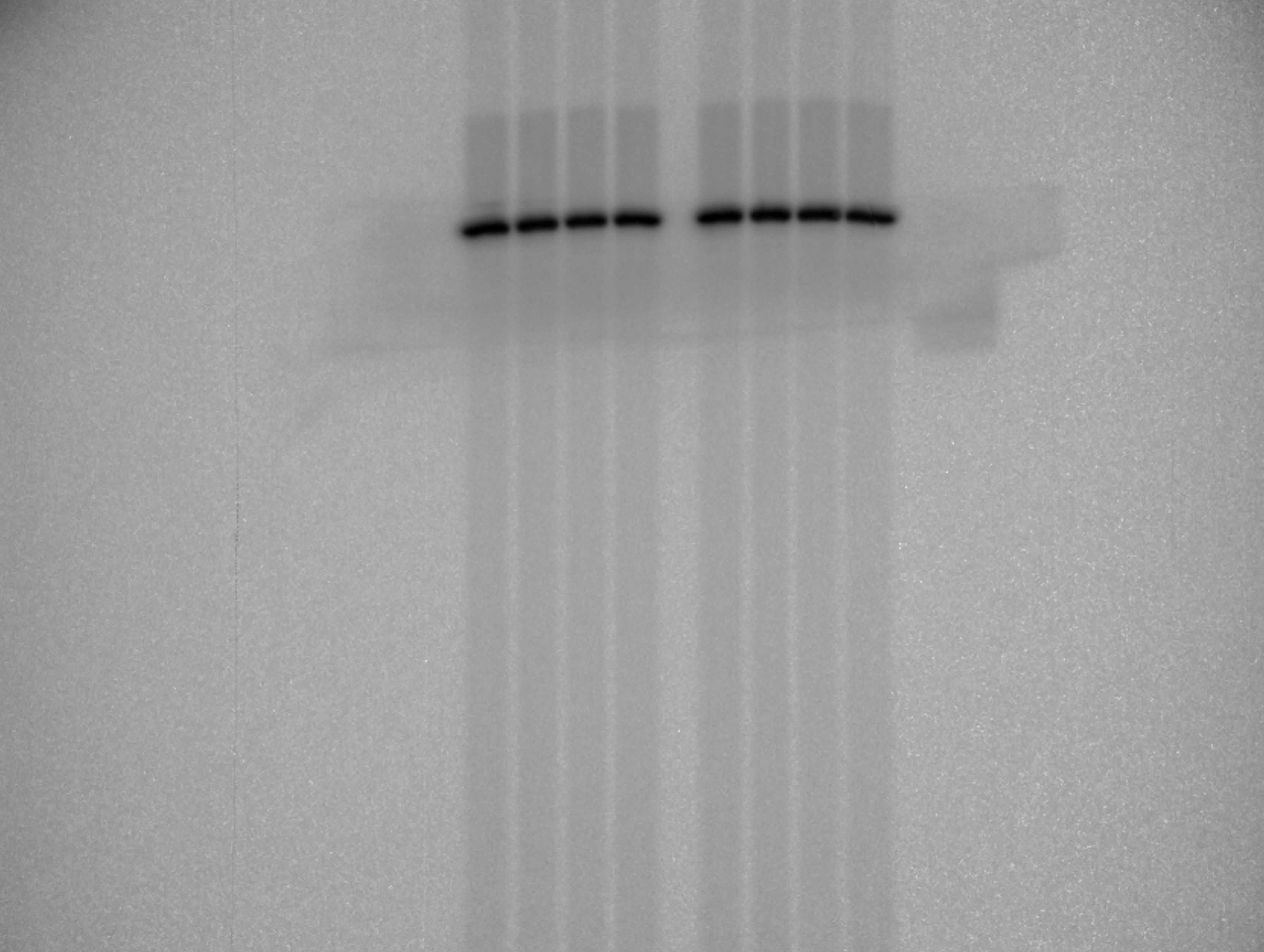

Supplement: Supplementary file 3 [file Datasheet3.zip › original raw files of Supplementary Figure 2A/membrane2-GAPDH.tif]

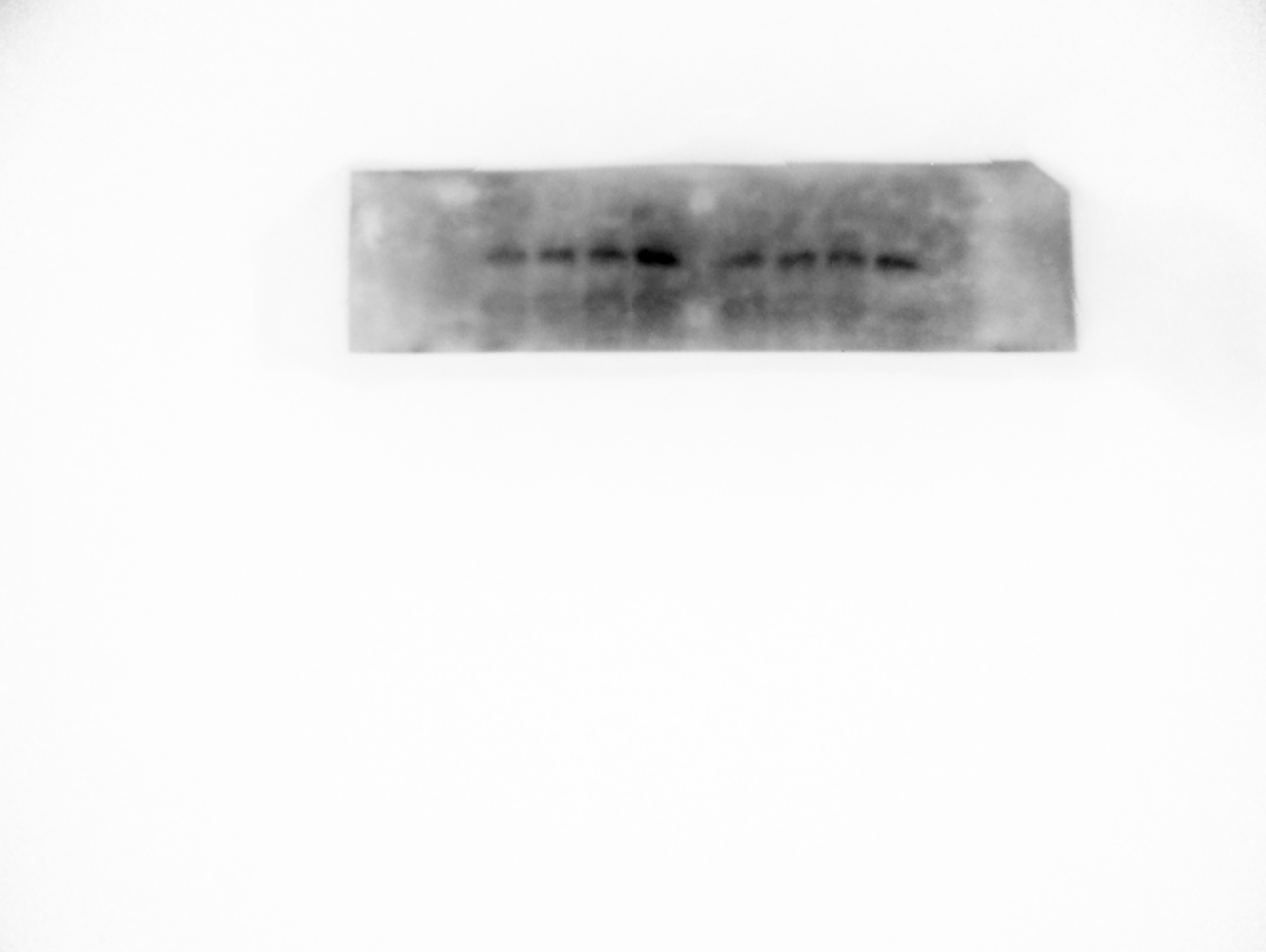

Supplement: Supplementary file 3 [file Datasheet3.zip › original raw files of Supplementary Figure 2A/membrane2-p21.tif]

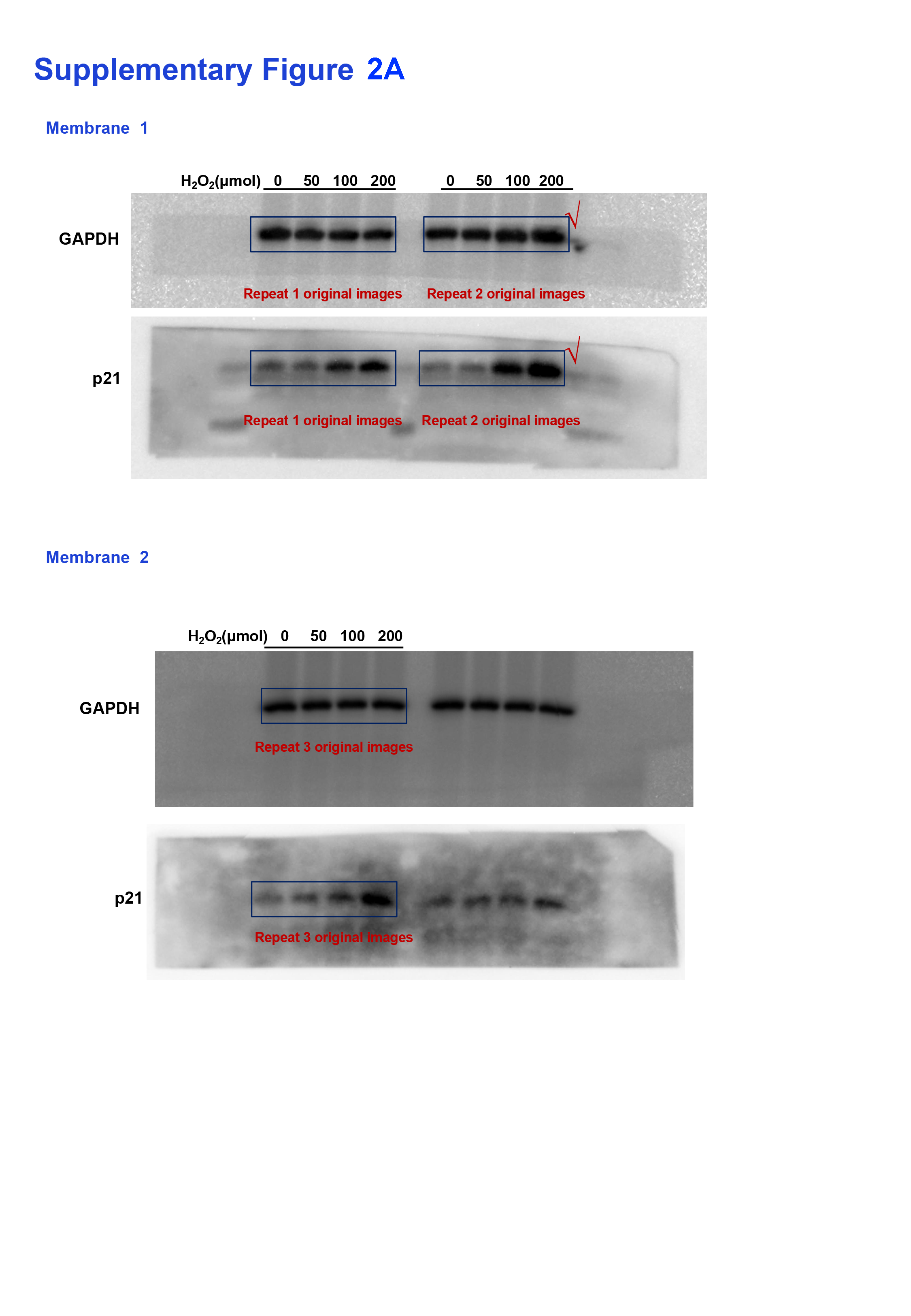

Supplement: Supplementary file 3 [file Datasheet3.zip › original raw files of Supplementary Figure 2A/Integration and annotation of the original images副本.tif]
