# Supplementary material for: N6-methyladenosine RNA landscape in the aged mouse hearts
Source: Front Cardiovasc Med. 2025 Jun 18;12:1563364. doi: 10.3389/fcvm.2025.1563364 (PMC12213823; doi:10.3389/fcvm.2025.1563364)
Supplement: Supplementary file 4 [file Datasheet4.pdf]

Supplementary Material

1 Supplementary Figures and Tables

1.1 Supplementary Figures

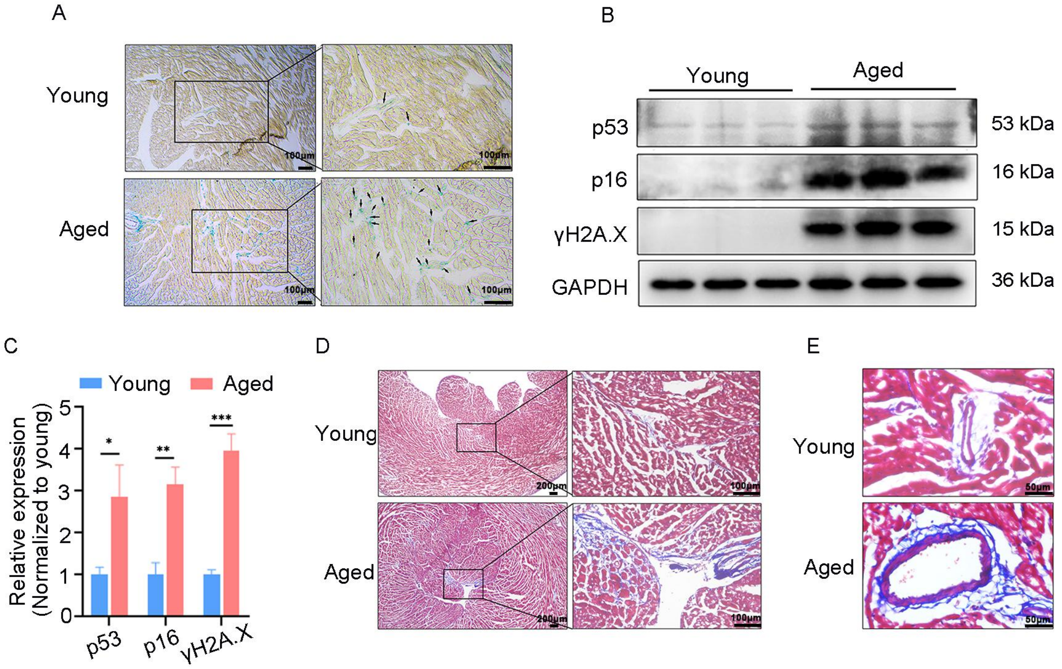

Supplementary Figure 1. Characterization of cardiac senescence and fibrosis in aged mice

(A) Senescent cells were detected by SA-β-gal staining in young and aged mouse heart. n=3 mice per group. (B, C) Protein expression levels and the corresponding densitometric analyses of p53, p16, and γ-H2A.X in heart tissue from young and aged mice. n=3 mice per group. GAPDH was detected as the loading control. $*P < 0.05$, $**P < 0.01$, $***P < 0.001$ by Student's t-test. (D, E) Representative photomicrographs of Masson's trichrome staining performed on heart sections to assess interstitial fibrosis (D), and perivascular collagen volume area (E) in young and aged mice. n=3 mice per group.

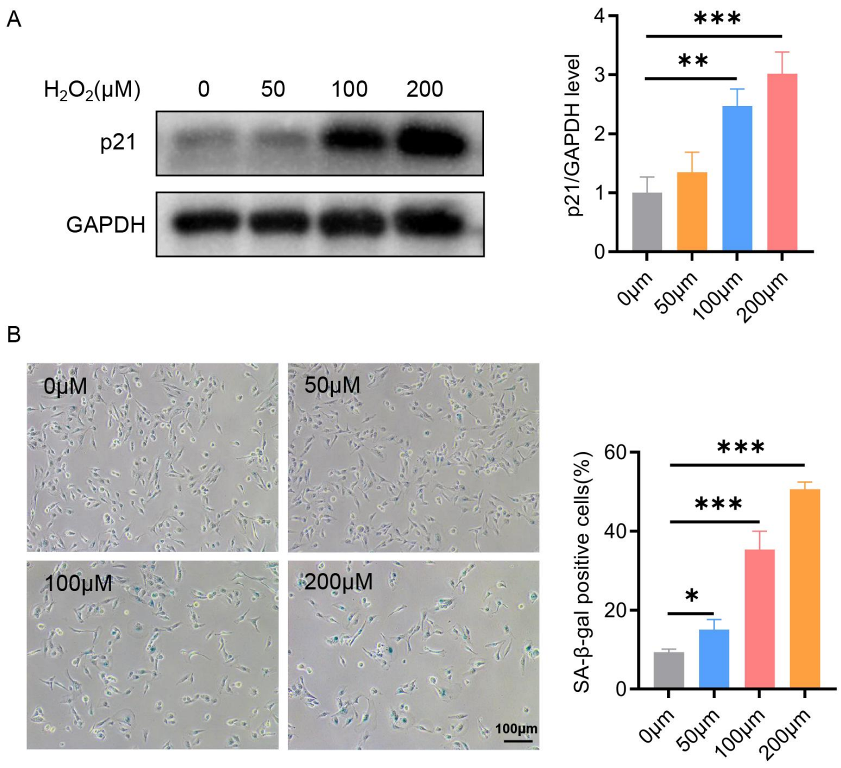

Supplementary Figure 2. Detection in AC16 Cells Treated with H_2O_2

(A) After AC16 cells were treated with H_2O_2, the protein levels of p21 were detected by Western blot, with GAPDH detected as the loading control. (B) SA - β - gal staining was performed.

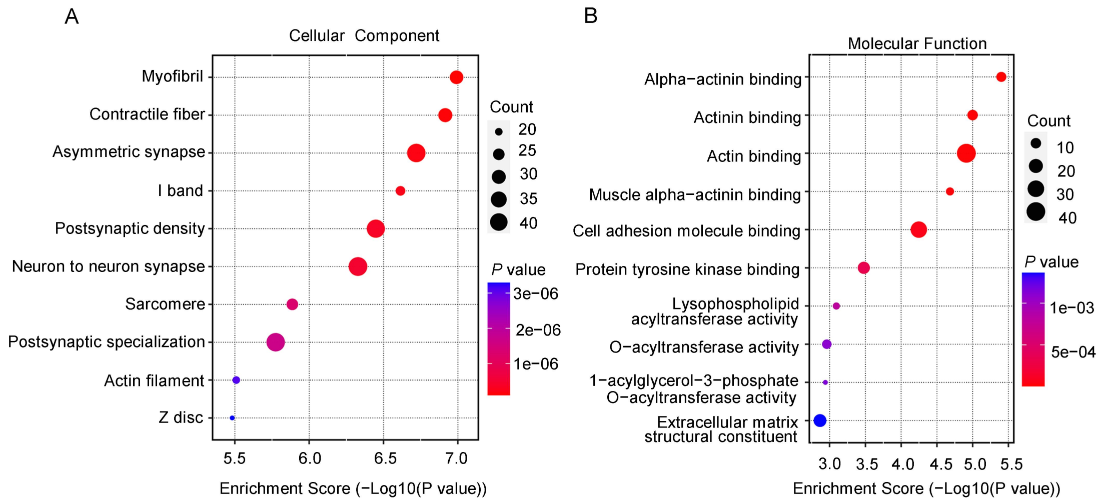

Supplementary Figure 3. GO enrichment analysis of differentially expressed genes.

(A) The top 10 GO cellular component enrichment analysis of differentially expressed genes. (B) The top 10 GO molecular function enrichment analysis of differentially expressed genes.

1.2 Supplementary Tables

Table S1. The primer sequences for qRT-PCR

Species	Gene	forward/reverse	Sequence (5’ to 3’)
Human	ACTIN	forward	AGATGACCCAGATCATGTTTGAG
Human	ACTIN	reverse	AGGGCATACCCCTCGTAGAT
Human	EFEMP1	forward	GACGCACAACTGTAGAGCAGAC
Human	EFEMP1	reverse	GAGCCTGGTGTATTCACGCATC
Mouse	Actin	forward	GTGACGTTGACATCCGTAAAGA
Mouse	Actin	reverse	GCCGGACTCATCGTACTCC
Mouse	Efemp1	forward	CACACCTGGTTCCTTCTACTGC
Mouse	Efemp1	reverse	AGCACACTGGTTGCTGGCATCA
